# Shining a Light on Peptide and Protein Synthesis: Light-Emitting-Diode-Driven Desulfurization of Cysteine to Alanine with Rose Bengal

**DOI:** 10.1021/acs.orglett.4c04671

**Published:** 2025-01-23

**Authors:** Mateusz Waliczek, Piotr Stefanowicz

**Affiliations:** Faculty of Chemistry, University of Wrocław, Joliot-Curie 14 Street, 50-383 Wrocław, Poland

## Abstract

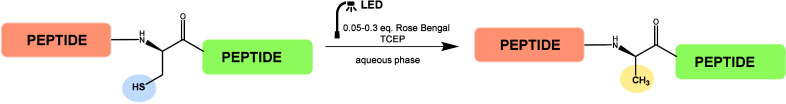

Studies presenting visible-light-induced desulfurization of peptides containing a cysteine residue have been carried out. This transformation driven by light-emitting-diode-type light proceeds with high efficiency in an aqueous solution at room temperature and involves the use of a catalytic amount of photosensitizer, Rose Bengal. The procedure has been tested on model synthetic peptides, lysozyme C and α-crystallin, and successfully applied to a one-pot native chemical ligation (NCL)–desulfurization protocol.

The chemical synthesis of peptides has been developing for many decades and has intensified with the introduction of the solid-phase peptide synthesis (SPPS) method.^1^ This method has made it possible to obtain tailor-made peptides along with a myriad of chemical modifications, which distinguish it from biotechnological methods that rely on natural modifications. However, the synthesis of large polypeptides or small proteins on a solid support is widely regarded as a challenge. This is due to the coiling of the long peptide chain on the solid support, which leads to a dramatic decrease in acylation efficiency and the formation of byproducts. To overcome this problem, techniques have been developed to condense the shorter segments obtained on a solid support. The real breakthrough turned out to be the development of chemical native ligation (NCL) by Kent et al.^2^ in the 1990s. This condensation involves the chemoselective reaction of an unprotected peptide containing a C-terminal thioester with a peptide containing a N-terminal cysteine in an aqueous solution.^3,4^ In the following years, the method was refined in terms of obtaining thioesters, e.g., thioester surrogates, yields, and reaction kinetics, e.g., replacing thioesters with selenoesters, and then extended to peptide sequences that do not contain cysteine residues because this amino acid is quite rare.^5−8^ Desulfurization of cysteine to alanine is still a pivotal tool today for accessing polypeptides and proteins that do not contain cysteine. Since then, several desulfurization methods have been developed (Figure 1). Some of the first described attempts to convert cysteine to alanine involved the use of Ranel nickel or palladium and H_2_, but this method is currently not popular due to its many limitations and has been replaced by newer methods.^9,10^ It was not until the method developed by Wan and Danishevsky that it gained prominence and was particularly popular among NCL-type ligation researchers.^11^ A typical protocol to enable desulfurization involves the use of TCEP, a radical initiator (e.g., VA-044 and V-50), and a large excess of external thiol. In addition, the reaction system requires heating to several degrees to trigger the reaction, and average reaction times require the sample to be left overnight.^12^ Sun et al.^13^ recently presented an interesting desulfurization method involving NaBEt_4_ and TCEP. While most of the previously described methods run in 8–16 h, this method proved to be revolutionary in terms of reaction kinetics and enables efficient Cys → Ala conversion in less than 1 min. The main drawback of the method, however, is the physicochemical properties of this reagent, described as extremely pyrophoric and unstable in air.

**Figure 1:**
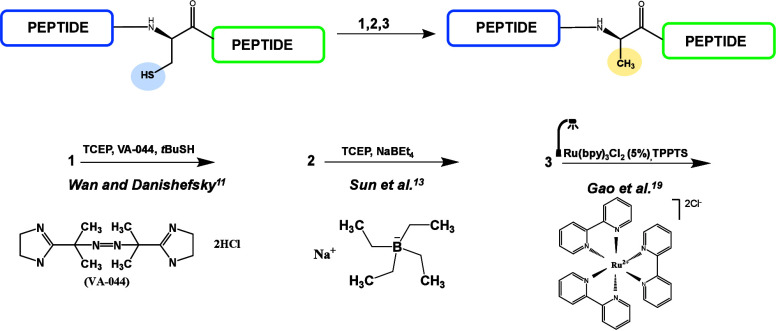
Diagram of examples of cysteine desulfurization methods.

Reactions using visible light have gained in importance in recent years.^14−18^ Gao et al.^19^ reported visible-light-induced desulfurization of cysteinyl peptide using a Ru(bpy)_3_^2+^ catalyst and 3,3′,3″-phosphinidynetris(benzenesulfonic acid) trisodium salt (TPPTS) as a phosphine source. Irradiation of peptides containing a cysteine residue with a household light bulb for a few to several hours under these conditions led to desulfurization in good yields. However, the authors observed that the use of TCEP, a common phosphine in peptide chemistry, did not result in an efficient reaction product. Visible-light-induced reactions can be mediated by the use of a photosensitizer, e.g., Rose Bengal (RB).^20−22^ A field of science that uses various photosensitizers (e.g., flavins and fluorescein) to catalyze organic reactions has developed rapidly in recent years.^23−25^

We herein present a new method for light-emitting diode (LED)-induced desulfurization of cysteinyl peptides and proteins using widely used and easily accessible TCEP and the photosensitizer RB. On the basis of the knowledge of desulfurization from a mechanistic point of view, in our study, we investigated specific desulfurization of Cys-containing peptides using visible light and the photosensitizer RB. We started our research with the synthesis of the model peptide H-Thr-Gly-Cys-Ala-Phe-Lys-NH_2_ (see Figures S1–S3 of the Supporting Information) on the solid support. For this purpose, a commonly used Fmoc-based protocol was applied. With the purified model in hand, we tested the reaction using an excess of the water-soluble phosphine TCEP, 0.2 equiv of RB, and a large excess (350 equiv) of sodium 2-mercaptoethanesulfonate (MESNa) as a hydrogen source. The resulting mixture was irradiated in a high-performance liquid chromatography (HPLC)-type glass vial with a household LED lamp (6500 K) for 3 h. Thus, we assumed the possibility of generating thiyl radicals as a result of RB excited by visible light. Then, a small volume of the mixture was taken, and after dilution with water, the sample was analyzed by liquid chromatography–mass spectrometry (LC–MS) (see Figures S4–S6 of the Supporting Information). As expected, we observed a signal from the desired product on the chromatogram, that is, the peptide in which the cysteine residue was converted to alanine. However, the conversion efficiency was unsatisfactory, and the sample was dominated by an unreacted substrate. Due to the presence of the product in the analyzed mixture, the reaction was optimized. In the first step, the effect of the amount of excess external thiol on the course of photochemical transformation was studied (see Figures S7–S11 of the Supporting Information). Surprisingly, it was found that, as the amount of MESNa was reduced, the conversion efficiency increased. Finally, the study showed that the reaction proceeded with high efficiency in the absence of MESNa, suggesting that this reactant hindered the transformation and that the thiol group was not a donor of hydrogen required for alanine formation. The desulfurization was optimized for the amount of TCEP phosphine needed in the next step (see Figures S12–S17 of the Supporting Information). Interestingly, even a 10-fold reduction in the amount of phosphine still led to efficient desulfurization. Desulfurization protocols known in the literature typically involve the use of 0.1–0.5 M TCEP. In this case, even the 0.01 M concentration (Figure 2) used was equally effective. Subsequent experiments involved testing the required amount of the RB photosensitizer. In previously tested samples, 0.2 equiv of RB were applied to the peptide. A test was performed using 0.2, 0.1, and 0.05 equiv of RB. LC–MS analysis of the samples taken showed an almost identical chromatographic image for all mixtures (see Figures S18 and S19 of the Supporting Information). The reactions shown above were carried out in an aqueous solution at pH 5, which is the most commonly recommended in the desulfurization protocols described thus far. Therefore, the final optimization step was to run the reactions at different pH parameters. Visible-light-induced transformation of cysteine was carried out at pH 4, 5, 6, and 7 (see Figures S20–S23 of the Supporting Information). For this model peptide, the reaction proceeded efficiently over the pH range tested. Another optimized parameter was the sample irradiation time. For this purpose, the sample was taken and analyzed with LC–MS at selected intervals (Figures S24–S27 of the Supporting Information). The results obtained indicate that the optimal exposure time is 3 h. During this time, more than 90% of the substrate undergoes Cys → Ala conversion, while shortening the photochemical reaction leads to a decrease in the reaction yield. To test the feasibility of the method on other models, the optimized desulfurization conditions were then applied to the following sequences: H-Ala-Phe-Cys-NH_2_ (Figures S29–S45 of the Supporting Information), H-Thr-Cys-Phe-Ala-Glu-Gly-Lys-OH (Figures S46–S52 of the Supporting Information), H-Cys-Glu-Leu-Phe-Glu-Gln-Leu-Gly-Glu-Tyr-Lys-OH (Figures S43–S59 of the Supporting Information), and H-Cys-Ile-Leu-Lys-Glu-Pro-Val-His-Gly-Val-NH_2_ (Figures S60–S66 of the Supporting Information). As expected, the irradiation led to the formation of the desired products with a yield of >90%.

**Figure 2:**
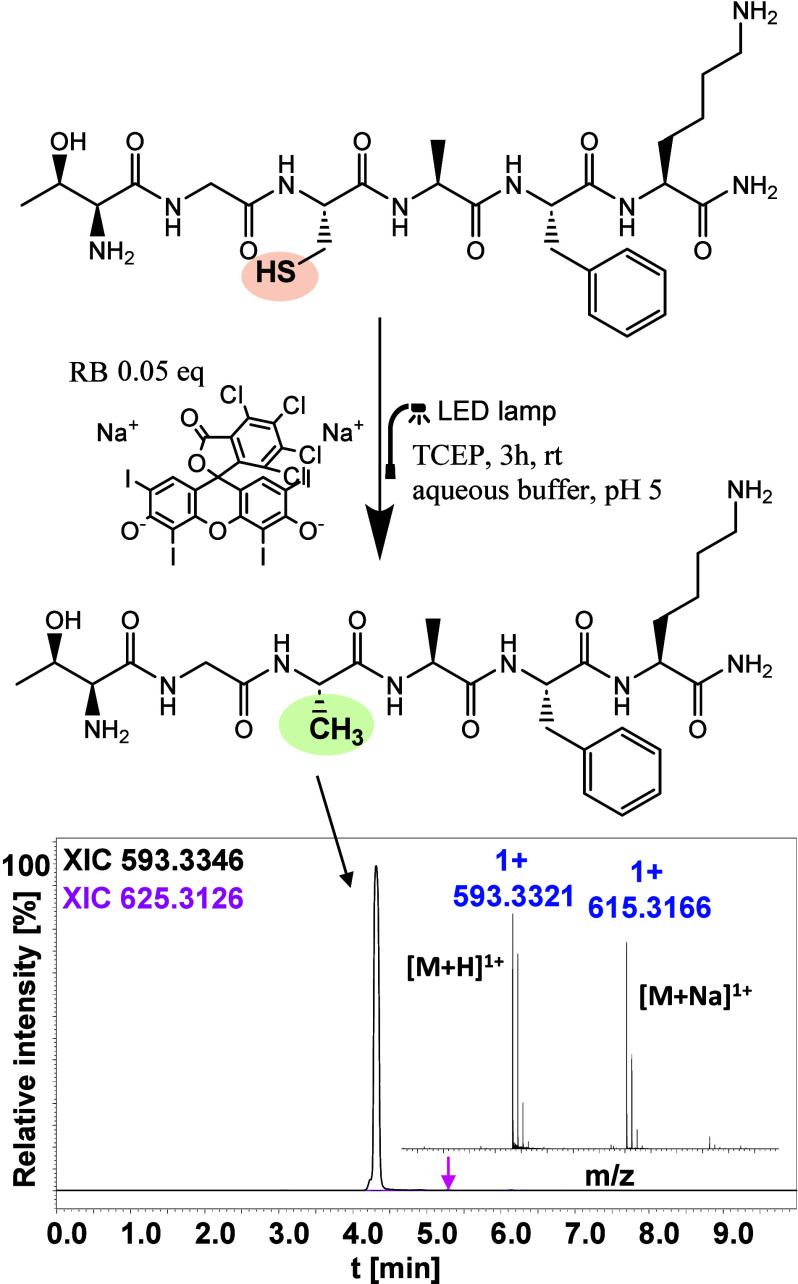
Scheme of visible-light-induced desulfurization of model peptide H-Thr-Gly-Cys-Ala-Phe-Lys-NH_2_. LC–MS [extracted ion chromatogram (XIC)] chromatogram obtained for the mixture after desulfurization and the electrospray ionization mass spectrometry (ESI–MS) spectrum of the reaction product.

As mentioned in the introduction, NCL has been an extremely important method for obtaining polypeptides or smaller proteins for many years, and in combination with desulfurization, it allows access to a wide range of desired sequences. The next step was to demonstrate one-pot NCL and visible-light-induced desulfurization. We started the experiment with the SPPS synthesis of two peptide fragments. The first sequence contained a C-terminal hydrazide, while the second contained a N-terminal cysteine residue. The selenoester formation–ligation procedure was performed according to the method described by Li et al.^26^ This method involves the one-pot conversion of peptide hydrazide into a selenoester, which is ligated to the Cys-containing second fragment in a second step. Excess diphenyl diselenide (DPDS) is then extracted with diethyl ether. This treatment is necessary because of the scavenging properties of selenium compounds and the associated elimination of thiyl radicals during the reaction. After the addition of TCEP and a photosensitizer, the mixture was exposed to a LED lamp for 3 h. Due to the possible presence of a small amount of unremoved DPDS, the RB equivalent was increased to 0.3 equiv. The results of the LC–MS analysis are shown in Figure 3 (for more details, see Figures S67–S77 of the Supporting Information). Chromatogram analysis indicates that the ligated peptide was converted very efficiently (93%) to an alanine-containing analogue. The structure of the obtained sequence was further confirmed by tandem mass spectrometry (MS/MS) fragmentation. In addition, noteworthy is the presence of tryptophan in the sequence of the peptide, which remained intact throughout the NCL–desulfurization procedure. This shows that the presented method can also be applied to sequences containing more sensitive amino acids.

**Figure 3:**
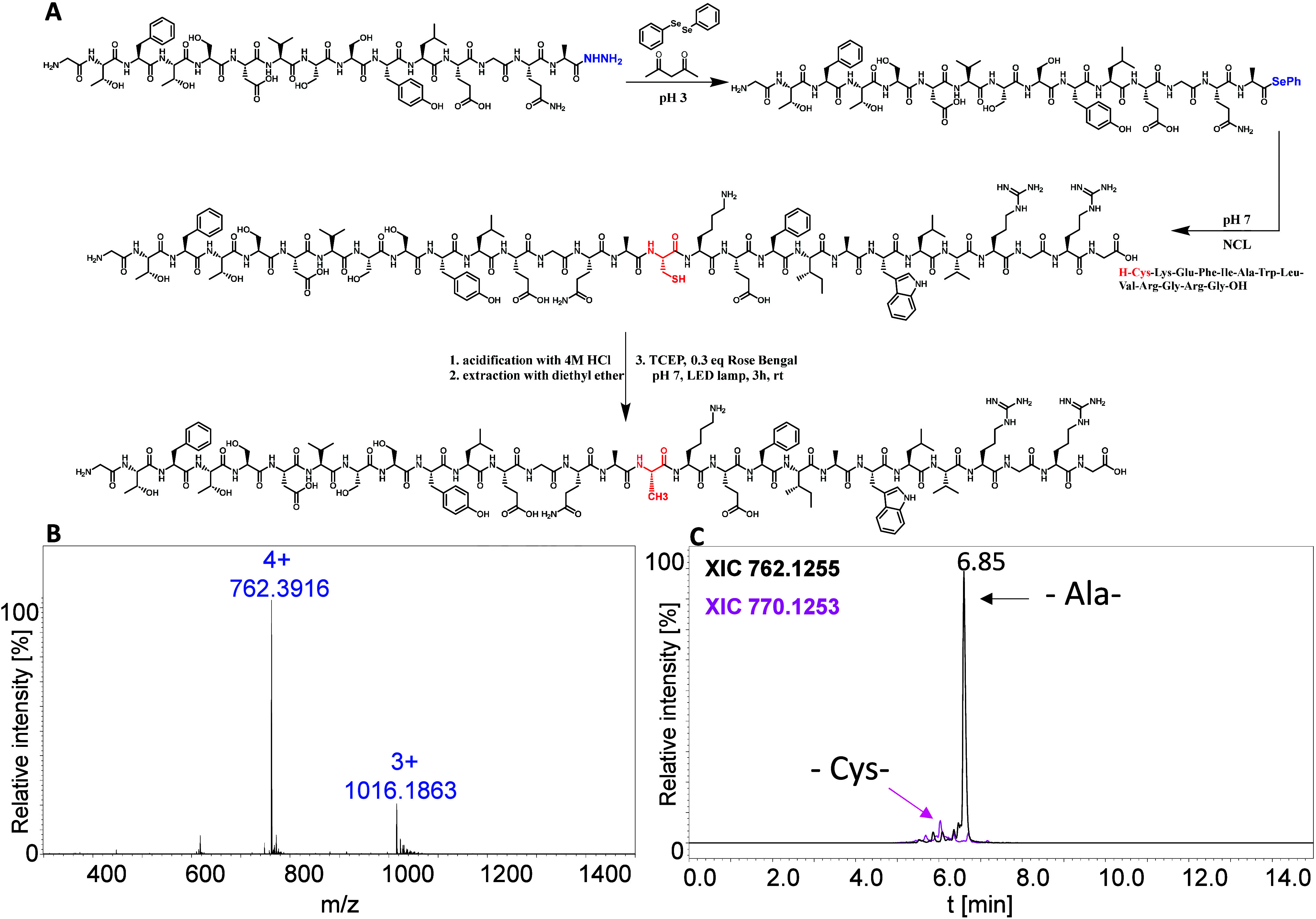
(A) Schematic representation of one-pot NCL–desulfurization, (B) ESI–MS spectrum of the reaction product, and (C) LC–MS (XIC) chromatogram obtained for the mixture after the desulfurization protocol.

A typical desulfurization mechanism involves the abstraction of hydrogen with the generation of the thiyl radical, which reacts with phosphine, generating, in turn, the phosphoranyl radical. The decomposition of this radical results in the formation of an alanyl radical and a phosphine sulfide. The final step involves hydrogen abstraction by the alanyl radical with the formation of native alanine (usually from an external thiol). Experiments carried out during optimization showed that no external thiol was required. While this information obtained is positive in that it simplifies the method, it raises the question of where hydrogen attached to the thiyl radical in the final step comes from. Hence, to better understand the mechanism of the reaction, a desulfurization experiment of model peptide H-Cys-Glu-Leu-Phe-Glu-Gln-Leu-Gly-Glu-Tyr-Lys-OH was carried out in D_2_O. After the reaction, the peptide was captured using C18 OMIX, eluted, then dissolved in a mixture of H_2_O/ACN + 0.1% HCOOH, and analyzed by LC–MS. Analysis of the MS data revealed the presence of a heavy atom in the reaction product, associated with the insertion of one deuterium into the methyl group of alanine (see Figures S78 and S79 of the Supporting Information). RB was definitively rejected as a source of hydrogen because it was used in sub-stoichiometric quantities. We, therefore, assume that the reaction is a chain reaction, involving the formation of a thiyl radical with RB, the reaction of the radical with TCEP, and the formation of an alanyl radical, which, in turn, removes hydrogen from the sulfhydryl group of the next peptide molecule.

On the basis of the previous study, we also explored visible-light-induced desulfurization on proteins: α-crystalline and lysozyme C. Using the developed protocol, desulfurization of 1 mg of proteins was carried out. To determine the exact course of this modification, we subjected the post-reaction mixture to enzymatic digestion with trypsin. Due to the presence of several cysteine residues in the molecule, the amount of RB was increased to 0.3 equiv. The resulting protein digest was subjected to ultra-high-performance liquid chromatography–tandem mass spectrometry (UHPLC–MS/MS) analysis (see Figures S80–S82 of the Supporting Information), and the results obtained were bioinformatically analyzed using PEAKS Studio X+ software. Figure 4 shows the data obtained, which indicate that the α-crystallin containing one cysteine was quantitatively transformed into an analogue with alanine. On the other hand, in the lysozyme containing eight cysteines, seven residues of this amino acid were identified and modified with excellent efficiency (95–100%). The one cysteine residue located at the end of the lysozyme C sequence was not identified probably due to the high hydrophilicity of the tetrapeptide sequence, which was eluted with the dead volume of the column. However, on the basis of the data obtained, it can be assumed that this cysteine was also desulfurized.

**Figure 4:**
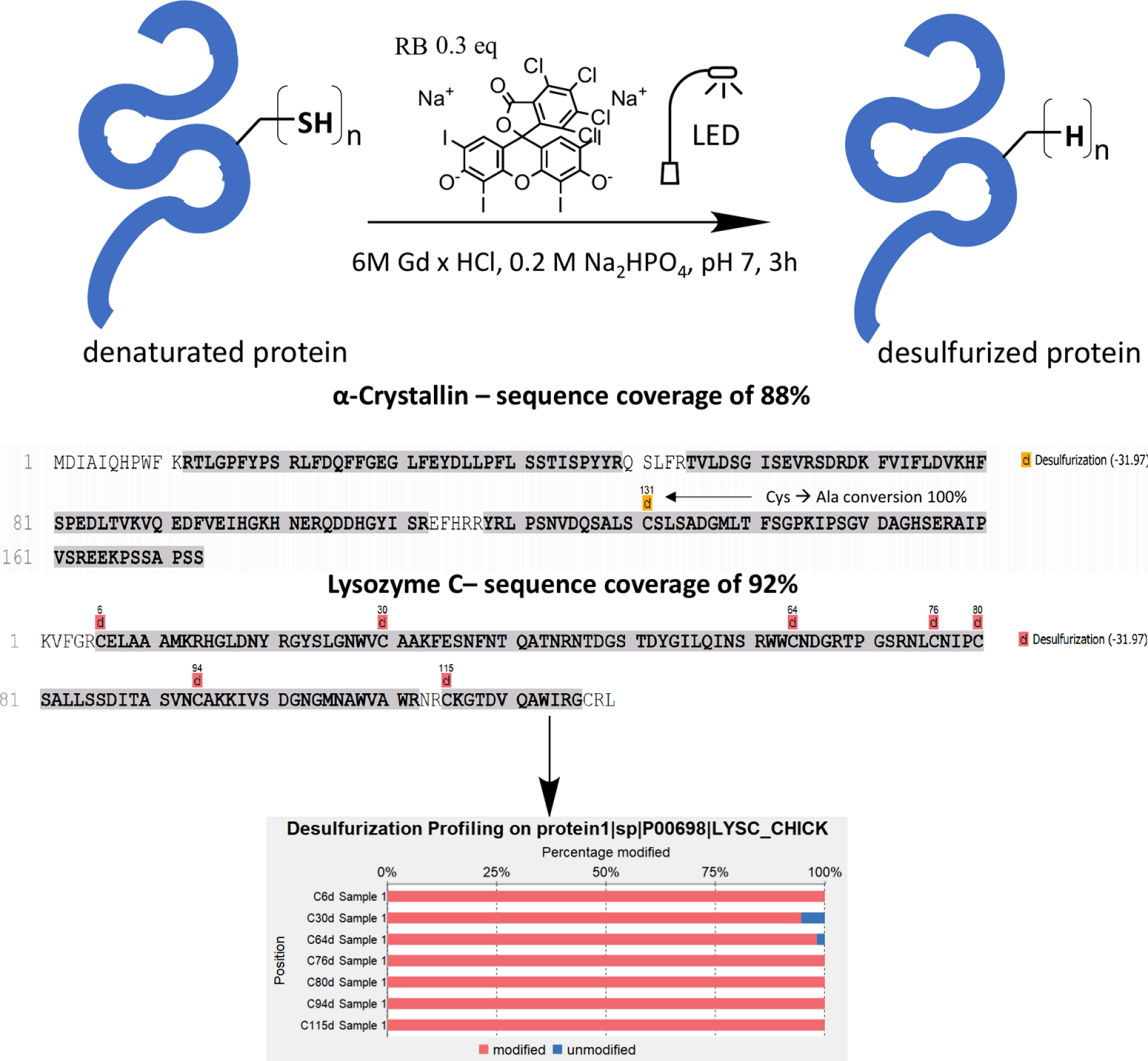
Schematic representation of protein desulfurization, sequence coverage of α-crystallin tryptic digest with desulfurized cysteine residue highlighted, sequence coverage of lysozyme C tryptic digest with desulfurized cysteine residues highlighted, and table showing the percentage efficiency of desulfurization of lysozyme C depending upon the position in the sequence.

In conclusion, we have developed an efficient method for visible-light-induced desulfurization of cysteinyl peptides and proteins, which proceeds with a yield of >95%. The reaction is selective for cysteine and can be successfully used in a NCL–desulfurization protocol. Importantly, the developed cost-effective method requires much smaller amounts of phosphine TCEP than previously developed methods and a sub-stoichiometric amount of the readily available photosensitizer RB. In addition, the reaction proceeds with reasonable kinetics in 3 h and does not require the addition of an external thiol.

## Data Availability

The data underlaying this study are available in the published article and its online Supporting Information.

## Supporting Information Available

Detailed experimental procedures, HPLC and LC–MS chromatograms, and ESI–MS spectra (PDF)
