# Supplementary material for: Shining a Light on Peptide and Protein Synthesis: Light-Emitting-Diode-Driven Desulfurization of Cysteine to Alanine with Rose Bengal
Source: Org Lett. 2025 Jan 23;27(5):1159–63. doi: 10.1021/acs.orglett.4c04671 (PMC11811997; doi:10.1021/acs.orglett.4c04671)
Supplement: Supplementary file 1 — ol4c04671_si_001.pdf [file ol4c04671_si_001.pdf]

Shining a Light on Peptide and Protein Synthesis: LED-Driven Desulfurization of Cysteine to Alanine with Rose Bengal

Mateusz Waliczek* and Piotr Stefanowicz

Faculty of Chemistry, University of Wrocław, Joliot-Curie 14 Street, 50-383 Wrocław, Poland

CONTENTS

1. Reagents and Materials
2. HPLC analysis
3. LC-MS analysis
4. Mass spectrometry analysis
5. Purification of peptides
6. NMR
7. Preparation of peptides
   7.1 Preparation of model peptide H-Thr-Gly-Cys-Ala-Phe-Lys-NH_2
   7.2 Preparation of tetrapeptide H-Ala-Phe-Cys-NH_2
   7.3 Preparation of peptide *H-Glu-Thr-Cys-Phe-Ala-Glu-Glu-Gly-Lys-OH*
   7.4 Preparation of peptide H-Cys-Glu-Leu-Phe-Glu-Gln-Leu-Gly-Glu-Tyr-Lys-OH
   7.5 Preparation of peptide H-Cys-Ile-Leu-Lys-Glu-Pro-Val-His-Gly-Val-NH_2
   7.6 Preparation of peptide H-Cys-Lys-Glu-Phe-Ile-Ala-Trp-Leu-Val-Arg-Gly-Arg-Gly-OH
   7.7 *Preparation of peptide with C-terminal hydrazide H-Gly-Thr-Phe-Thr-Ser-Asp-Val-Ser-Ser-Tyr-Leu-Glu-Gly-Gln-Ala-NHNH$_2$*
8. General procedure for desulfurization of cysteinyl peptides
9. One-pot native chemical ligation (NCL)-desulfurization
10. Desulfurization of peptide H-Thr-Gly-Cys-Ala-Phe-Lys-NH_2 in D_2O.
11. The procedure for desulfurization of proteins
12. Protein tryptic digestion

13. Bioinformatics analysis
14. Results
14.1 Synthesis of model peptide H-Thr-Gly-Cys-Ala-Phe-Lys-NH_2
14.2 Optimization of desulfurization – addition of external thiol MESNa
14.3 Optimization of desulfurization – addition of TCEP
14.4 Optimization of desulfurization – addition of Rose Bengal
14.5 Optimization of desulfurization – change of pH
14.6 Optimization of desulfurization – time of exposure to visible light
14.7 Desulfurization of tetrapeptide H-Thr-Gly-Cys-Ala-Phe-Lys-NH_2
14.8 Desulfurization of tetrapeptide H-Ala-Phe-Cys-Gly-NH_2
14.9 Desulfurization of peptides H-Thr-Cys-Phe-Ala-Glu-Glu-Gly-Lys-OH
14.10 Desulfurization of peptides H-Cys-Glu-Leu-Phe-Glu-Gln-Leu-Gly-Glu-Tyr-Lys-OH
14.11 Desulfurization of peptide H-Cys-Ile-Leu-Lys-Glu-Pro-Val-His-Gly-Val-NH_2
14.12 One-pot native chemical ligation (NCL)-desulfurization
15. Mechanistic study of desulfurization
16. Desulfurization of proteins

1. Reagents and Materials

All commercially available reagents were used without further purification and water was deionized by reversed osmotic system (Hydrolab). Solvents for synthesis dimethylformamide – DMF, dichloromethane – DCM, methanol – MeOH, tetrahydrofurane – THF, diethyl ether – Et_2O, *N*-Diisopropylethylamine – DIPEA, Piperidine – PIP, HCOOH, trifluoroacetic acid – TFA, , triisopropylsilane – TIS, 1,2-ethanedithiol – EDT in analytical grade were obtained from Sigma-Aldrich and acetic anhydride from Lachner. Fmoc-amino acid derivatives for peptide synthesis were purchased from PeptideWeb (Peptydy.pl). Coupling reagent: PyBOP – benzotriazol-1-yl-oxytripyrrolidinophosphonium hexafluorophosphate, the resins for SPPS: H-Rink amide ChemMatrix® resin (0.40–0.60 mmol/g) and Trityl-OH ChemMatrix® resin (0.40–0.60 mmol/g) was purchased from Sigma-Aldrich and, tris(2-carboxyethyl)phosphine – TCEP and Rose Bengal were

purchased from Sigma-Aldrich, disodium phosphate was purchased from Sigma Aldrich. NaOH from Stanlab, in analytical grade HCl from Sigma-Aldrich. Lysozyme C from chicken egg, α-crystallin from bovine lens, TPCK-treated trypsin, chymotrypsin were purchased from Sigma-Aldrich. Solvents for LC-MS: MeCN, H_2O, and HCOOH in HPLC grade were purchased from Sigma-Aldrich and MeOH from J. T. Baker.

2. *HPLC analysis*

The HPLC analyzes were carried out using the Shimadzu Nexera X2 UPLC system equipped with UV control using a PDA detector (chromatograms recorded in the range of 190-380 nm). The LC system was operated with a mobile phase consisting of solvent A: 0.1% formic acid in H_2O and solvent B: 0.1% formic acid in MeCN. The separation conditions were as follows: The gradient conditions (B %) were from 0 to 30% B within 15 min - gradient 1. For the synthetic peptides obtained for native chemical ligation (NCL), the following separation conditions were used: 10 to 80% B within 15 min - gradient 2. The flow rate was 0.3 mL/min and the injection volume was 1 μL. For longer peptide the following gradient conditions from 0 to 50% B within 15 min were used- gradient 3. The separation was performed on an Phenomenex Aeris Peptide XB-C18 column (1000 mm × 2.1 mm) with 3.6 μm bead diameter. Peptide samples were dissolved in 400 μL of water : acetonitrile mixture (95:5).

3. *LC-MS analysis*

The LC-MS analyses of the peptides obtained were performed on a Shimadzu 9030, a hybrid system consisting of a quadrupole and a time-of-flight mass analyzer. This instrument is also equipped with an electrospray ion source (ESI). The potential between the spray needle and the orifice was set at 4.5 kV. Spectra were acquired in the m/z range of 200-1000. Fragmentation was performed by collision-induced dissociation (CID) using argon as the collision gas. The MS2 spectra were recorded with the following parameters: an accurate m/z value, a mass range of 200-1000 m/z, an isolation window of 5 Da, ion accumulation of 20 ms, and the collision energy was optimized individually for each ion between 20 and 30 eV. The LC system (Nexera X2 UPLC system) was operated with a mobile phase consisting of solvent A: 0.1% formic acid in H_2O and solvent B: 0.1% formic acid in MeCN. The separation conditions were as follows: The gradient conditions (B %) were from 0 to 30% B within 15 min - gradient 1. For the mixtures obtained from native

chemical ligation (NCL), the following separation conditions were used: 10 to 80% B within 15 min - gradient 2. The following separation conditions were used for protein digests: 0 to 50% B within 55 minutes - gradient 3. The flow rate (all synthetic peptides) was 0.3 mL/min and the injection volume was 1 µL, while the flow rate of 0.2 was used for protein digests. The separation was performed on a Phenomenex Aeris Peptide XB-C18 column (1000 mm × 2.1 mm) with 3.6 µm bead diameter (for model peptides) and 1.7 µm (for protein digests). Peptide samples were dissolved in 400 µL of H_2O : ACN (95:5). UV control using a PDA detector (190-380 nm) was performed in concert with MS analysis.

4. *Mass spectrometry analysis*

Mass spectra were measured in the Mass Spectrometry Laboratory of the Department of Chemistry, University of Wroclaw. A Brucker qTOF Compact instrument was used. The instrument is equipped with an electrospray ion source (ESI) and a hybrid analyzer consisting of a quadrupole coupled with a time-of-flight (TOF) analyzer. Its resolution is approximately 30,000 FWHM. Each measurement was preceded by a calibration procedure using the quadrature method. Fragmentation experiments were performed in a CID-type collision chamber using argon, and the collision energy was optimized in the range of 20-30 eV each time. The samples analyzed were prepared by dissolving approximately 100 μg of product in 1 ml of ACN/H_2O (50 : 50) + 0.1% HCOOH mixture. The solvents were of "LC-MS" purity. The measuring range was 200-3000 m/z. Spectra were collected in positive ion mode.

5. *Purification of peptides.*

All the products obtained on solid support were purified after cleavage purified by preparative reversed-phase HPLC on a Tosoh Bioscience TSKgel ODS-120T (21,5 mm × 30 cm, 10 µm, 120 Å (C18)) column, using the following solvent systems: S1 0.1% aqueous TFA, S2 80% acetonitrile + 0.1% TFA, linear gradient from 5 to 70% of B for 50 min, flow rate 7.0 ml/min, UV detection at 210 nm. The resulting fractions were collected and subjected to a lyophilization process. The identities of the products were confirmed by MS analysis using the above-described Bruker Compact mass spectrometer equipped with an electrospray (ESI) ionization source. The purity of peptides was analyzed using a Nexera

(Shimadzu) HPLC system with a UV detection (PDA - 210 nm) and an Aeris Peptide XB-C18 column (100 mm × 2.1 mm) 3.6 μm bead diameter.

6. NMR

^{1}H NMR and ^{13}C NMR spectra were recorded on a high field Bruker 500 MHz spectrometer equipped with a broadband inverse gradient probe head. Spectra were referenced to the residual solvent signal (methanol-d_4– 4.87 ppm). Structural assignments were made with additional information from COSY and HSQC experiments. The deuterated solvent was purchased from Sigma-Aldrich.

7. Preparation of model peptides:

7.1 Preparation of model peptide H-Thr-Gly-Cys-Ala-Phe-Lys-NH_2

The synthesis of peptide H-Thr-Gly-Cys-Ala-Phe-Lys-NH_2 was performed on solid support according to the standard Fmoc strategy and using Chemmatrix Rink Amide Resin. After swelling a portion of resin in DMF (30 min) Fmoc-protected amino acid (3 eq) was coupled using PyBOP (3 eq) and DIPEA (6 eq) in a syringe reactor for 20 min assisted by ultrasounds and followed by the filtration and 5 times resin washing with DMF. For Fmoc deprotection 25% PIP/DMF was added and the syringe was placed on an ultrasound bath for 3 min. Afterward, the resin was filtered and washed 7 times with DMF. In order to check the completeness of couplings, the Kaiser test was carried out. The above-described steps were repeated with subsequent amino acid residues until the desired sequence was obtained. Finally, the resin was shrunk with the following combination of solvents: DMF/DCM, DCM, DCM/THF, THF, THF/Et_2O, Et_2O, and then dried in a desiccator overnight. The peptide was cleaved from the resin with a standard TFA/H_2O/TIS/EDT mixture (92.5:2.5:2.5:2.5) mixture within 2 hours. In the next step, the crude peptide was precipitated with cold diethyl ether and centrifuged, and purified by preparative-scale RP-HPLC.

HR-ESI-MS calcd *m/z* 625.3126, found 625.3114 $[M+H]^{1+}$; **ESI-MS/MS** (CE 25eV): b_3 (calcd *m/z* 262.0856, found 262.0962), b_4 (calcd *m/z* 333.1227, found 333.1290), b_5 (calcd *m/z* 480.1911, found 480.1929), y_2 (calcd *m/z* 293.1972, found 293.2053), y_3 (calcd

m/z 364.2343, found 364.1378), a_5 (calcd *m/z* 452.1962, found 452.1987), **HPLC**: R_t= 5.35 min (gradient 1), **yield:** 75%, 30 mg

7.2 Preparation of tetrapeptide H-Ala-Phe-Cys-NH$_2$

The peptide *H-Ala-Phe-Cys-Gly-NH$_2$* was synthesized on solid support according to the standard Fmoc strategy and using Chemmatrix Rink Amide resin. The resin was swollen in DMF for 30 min. For coupling of consecutive amino acid residues, the following procedure was used: Fmoc-protected amino acid (3 eq), PyBOP (3 eq), and DIPEA (6 eq) were dissolved in DMF and placed in a syringe reactor for 20 min assisted by ultrasound, followed by filtration and 5x resin washing with DMF. For Fmoc deprotection, 25% PIP/DMF was added and the syringe was placed in an ultrasonic bath for 3 minutes. The resin was then filtered and washed 7 times with DMF. The Kaiser test was performed to check the completeness of the couplings. The above steps were repeated with subsequent amino acid residues until the desired sequence was obtained. Finally, the resin was shrunk with the following combination of solvents: DMF/DCM, DCM, DCM/THF, THF, THF/Et_2O, Et_2O and then dried overnight in a desiccator. The peptide was eluted from the resin with a standard TFA/H_2O/TIS/EDT mixture (92.5:2.5:2.5:2.5) within 2 hours. The crude peptide was then precipitated with cold diethyl ether and centrifuged and purified by preparative-scale RP-HPLC.

HR-ESI-MS calcd *m/z* 339.1485, found 339.1504 $[M+H]^{1+}$; **ESI-MS/MS** (CE 10eV): $[M+H-NH_3]^{1+}$(calcd *m/z* 322.1225, found 322.1242), y_2 (calcd *m/z* 268.1114, found 268.1136), z_2 (calcd *m/z* 251.085, found 251.0889), **HPLC**: R_t= 6.54 min (gradient 1), **^{1}H NMR** (500 MHz, methanol-d$_4$) δ 7.36 – 7.24 (m, 5H), 4.73 (dd, *J* = 8.7, 6.5 Hz, 1H), 4.50 (dd, *J* = 7.0, 5.2 Hz, 1H), 3.93 (q, *J* = 7.1 Hz, 1H), 3.22 (dd, *J* = 13.9, 6.5 Hz, 1H), 3.03 (dd, *J* = 13.9, 8.7 Hz, 1H), 2.93 (dd, *J* = 13.9, 5.2 Hz, 1H), 2.84 (dd, *J* = 13.9, 7.1 Hz, 1H), 1.54 (d, *J* = 7.1 Hz, 3H)., **^{13}C NMR** (126 MHz, methanol-d$_4$) δ 174.0, 173.1, 171.1, 138.1, 130.3, 129.7, 128.1, 56.6, 56.6, 50.1, 38.4, 26.9, 17.6., **yield:** 85%, 21 mg

7.3 Preparation of peptide H-Thr-Cys-Phe-Ala-Glu-Glu-Gly-Lys-OH

The peptide H-Thr-Cys-Phe-Ala-Glu-Glu-Gly-Lys-OH was synthesized on solid support according to the standard Fmoc strategy and using Chemmatrix Trityl-OH resin.

The resin was swollen in DCM for 30 min followed by the addition of freshly prepared 10% $SOCl_2$/DCM. The syringe was left on the rotator overnight. The next day the resin was washed with 3x DCM, 2x 5%DIPEA/DCM, and then left on the rotator in DMF for 20 min. After this time, the resin was loaded with the mixture of Fmoc-Lys(Boc)-OH (3 eq) and DIPEA (6 eq) for 2 hours. For Fmoc deprotection, 25% PIP/DMF was added and the syringe was placed in an ultrasonic bath for 3 minutes. For coupling of additional amino acid residues, the following procedure was used: Fmoc-protected amino acid (3 eq), PyBOP (3 eq), and DIPEA (6 eq) were dissolved in DMF and placed in a syringe reactor for 20 min assisted by ultrasound, followed by filtration and 5x resin washing with DMF. The resin was then filtered and washed 7 times with DMF. The Kaiser test was performed to check the completeness of the couplings. The above steps were repeated with subsequent amino acid residues until the desired sequence was obtained. Finally, the resin was shrunk with the following combination of solvents: DMF/DCM, DCM, DCM/THF, THF, THF/Et_2O, Et_2O and then dried overnight in a desiccator. The peptide was eluted from the resin with a standard TFA/H_2O/TIS/EDT mixture (92.5:2.5:2.5:2.5) within 2 hours. The crude peptide was then precipitated with cold diethyl ether and centrifuged and purified by preparative-scale RP-HPLC.

HR-ESI-MS calcd *m/z* 884.3818, found 884.3468 $[M+H]^{1+}$; **ESI-MS/MS** (CE 35eV): y_3-H_2O (calcd *m/z* 315.1663, found 315.1632), y_3 (calcd *m/z* 333.1769, found 333.1723), b_5-H_2O (calcd *m/z* 534.2017, found 534.2155), b_5 (calcd *m/z* 552.2123, found 552.2256), b_6-H_2O (calcd *m/z* 663.2984, found 663.2519), b_6 (calcd *m/z* 681.2549, found 681.2603), b_6 (calcd *m/z* 867.3553, found 867.3241) **HPLC**: R_t= 7.64 min (gradient 1), **yield:** 72%, 17 mg

7.4 Preparation of peptide H-Cys-Glu-Leu-Phe-Glu-Gln-Leu-Gly-Glu-Tyr-Lys-OH

The peptide *H-Cys-Glu-Leu-Phe-Glu-Gln-Leu-Gly-Glu-Tyr-Lys-OH* was synthesized on solid support according to the standard Fmoc strategy and using Chemmatrix Trityl-OH resin. The resin was swollen in DCM for 30 min followed by the addition of freshly prepared 10% $SOCl_2$/DCM. The syringe was left on the rotator overnight. The next day the resin was washed with 3x DCM, 2x 5%DIPEA/DCM, and then left on the rotator in DMF for 20 min. After this time, the resin was loaded with the mixture of Fmoc-Lys(Boc)-OH (3 eq) and DIPEA (6 eq) for 2 hours. For Fmoc deprotection, 25% PIP/DMF was added and the syringe was placed in an ultrasonic bath for 3 minutes. For coupling of additional amino

acid residues, the following procedure was used: Fmoc-protected amino acid (3 eq), PyBOP (3 eq), and DIPEA (6 eq) were dissolved in DMF and placed in a syringe reactor for 20 min assisted by ultrasound, followed by filtration and 5x resin washing with DMF. The resin was then filtered and washed 7 times with DMF. The Kaiser test was performed to check the completeness of the couplings. The above steps were repeated with subsequent amino acid residues until the desired sequence was obtained. Finally, the resin was shrunk with the following combination of solvents: DMF/DCM, DCM, DCM/THF, THF, THF/Et_2O, Et_2O and then dried overnight in a desiccator. The peptide was eluted from the resin with a standard TFA/H_2O/TIS/EDT mixture (92.5:2.5:2.5:2.5) within 2 hours. The crude peptide was then precipitated with cold diethyl ether, centrifuged, and purified by preparative-scale RP-HPLC.

HR-ESI-MS calcd *m/z* 679.8185, found 679.8137 $[M+2H]^{2+}$; **ESI-MS/MS** (CE 25eV): y_9 (calcd *m/z* 1126.5779, found 1126.5665), y_8 (calcd *m/z* 1113.4938, found 1113.4838), y_7(calcd *m/z* 866.4254, found 866.4166), y_6(calcd *m/z* 737.3828, 737.3744), y_5(calcd *m/z* 609.3243, found 609.6157), y_4(calcd *m/z* 496.2402, found 496.2305), y_3(calcd *m/z* 439.2181, found 439.2073), y_2(calcd *m/z* 310.1761, found 310.1593), b_6(calcd *m/z* 750.3127, found 750.3039), b_3(calcd *m/z* 346.1431, found 346.1282), **HPLC**: R_t= 10.79 min (gradient 3), **yield:** 70%, 14 mg

7.5 Preparation of peptide H-Cys-Ile-Leu-Lys-Glu-Pro-Val-His-Gly-Val-NH_2

The peptide *H-Cys-Ile-Leu-Lys-Glu-Pro-Val-His-Gly-Val-NH_2* was synthesized on solid support according to the standard Fmoc strategy and using Chemmatrix Rink Amide resin. The resin was swollen in DMF for 30 min. For coupling of consecutive amino acid residues, the following procedure was used: Fmoc-protected amino acid (3 eq), PyBOP (3 eq), and DIPEA (6 eq) were dissolved in DMF and placed in a syringe reactor for 20 min assisted by ultrasound, followed by filtration and 5x resin washing with DMF. For Fmoc deprotection, 25% PIP/DMF was added and the syringe was placed in an ultrasonic bath for 3 minutes. The resin was then filtered and washed 7 times with DMF. The Kaiser test was performed to check the completeness of the couplings. The above steps were repeated with subsequent amino acid residues until the desired sequence was obtained. Finally, the resin was shrunk with the following solvents: DMF/DCM, DCM, DCM/THF, THF, THF/Et_2O, Et_2O and then dried overnight in a desiccator. The peptide was eluted from the resin with a standard TFA/H_2O/TIS/EDT mixture (92.5:2.5:2.5:2.5) within 2

hours. The crude peptide was then precipitated with cold diethyl ether, centrifuged, and purified by preparative-scale RP-HPLC.

HR-ESI-MS calcd *m/z* 547.3130, found 547.3127 $[M+2H]^{2+}$; **ESI-MS/MS** (CE 25eV): y_8 (calcd *m/z* 877.5254, found 877.5241), y_7 (calcd *m/z* 764.4413, found 764.4393), y_6 (calcd *m/z* 636.3464, found 636.3434), y_5 (calcd *m/z* 507.3038, found 507.2986), y_3 (calcd *m/z* 311.1826, found 311.1688), $b_{9(2+)}$ (calcd *m/z* 920.2655, found 489.2601), b_8 (calcd *m/z* 920.5022, found 920.5003), b_7 (calcd *m/z* 783.4433, found 783.4412), b_6 (calcd *m/z* 684.3749, found 684.3739), b_5 (calcd *m/z* 587.3221, found 587.3186), **HPLC**: R_t= 10.10 min (gradient 3), **yield:** 67%, 13 mg

7.6 Preparation of peptide H-Cys-Lys-Glu-Phe-Ile-Ala-Trp-Leu-Val-Arg-Gly-Arg-Gly-OH

The synthesis of the peptide H-Cys-Lys-Glu-Phe-Ile-Ala-Trp-Leu-Val-Arg-Gly-Arg-Gly-OH was performed on solid support according to the standard Fmoc strategy and using Chemmatrix Trityl-OH resin. The resin was swollen in DCM for 30 min followed by the addition of freshly prepared 10% $SOCl_2$/DCM. The syringe was left on the rotator overnight. The next day the resin was washed with 3x DCM, 2x 5%DIPEA/DCM, and then left on the rotator in DMF for 20 min. After this time, the resin was loaded with the mixture of Fmoc-Lys(Boc)-OH (3 eq) and DIPEA (6 eq) for 2 hours. For Fmoc deprotection, 25% PIP/DMF was added and the syringe was placed in an ultrasonic bath for 3 minutes. For coupling of additional amino acid residues, the following procedure was used: Fmoc-protected amino acid (3 eq), PyBOP (3 eq), and DIPEA (6 eq) were dissolved in DMF and placed in a syringe reactor for 20 min assisted by ultrasound, followed by filtration and 5x resin washing with DMF. The resin was then filtered and washed 7 times with DMF. The Kaiser test was performed to check the completeness of the couplings. The above steps were repeated with subsequent amino acid residues until the desired sequence was obtained. Finally, the resin was shrunk with the following combination of solvents: DMF/DCM, DCM, DCM/THF, THF, THF/Et_2O, Et_2O and then dried overnight in a desiccator. The peptide was eluted from the resin with a standard TFA/H_2O/TIS/EDT mixture (92.5:2.5:2.5:2.5) mixture within 2 hours. The crude peptide was then precipitated with cold diethyl ether, centrifuged, and purified by preparative-scale RP-HPLC.

HR-ESI-MS calcd *m/z* 767.9192, found 767.9143 $[M+2H]^{2+}$; calcd *m/z* 512.2819, found 512.2776 $[M+3H]^{3+}$; **ESI-MS/MS** (*m/z* 512.2776, CE 25eV): b_3-H_2O (calcd *m/z* 343.1435, found 343.1492), y_4-NH_3 (calcd *m/z* 428.2364, found 428.2399), y_5 (calcd *m/z* 544.3314, found 544.3324), y_5-NH_3 (calcd *m/z* 527.3049, found 527.3054), y_6-NH_3 (calcd *m/z* 640.3889, found 640.3882), y_7 (calcd *m/z* 843.4948, found 843.4932), y_7-NH_3 (calcd *m/z* 826.4682, found 826.4666), y_8 (calcd *m/z* 914.5319, found 914.5301), y_8-NH_3 (calcd *m/z* 897.5053, found 897.5038), y_8(2+) (calcd *m/z* 457.7696, found 457.7721), y_9 (calcd *m/z* 1027.6160, found 1027.6152), y_9-NH_3 (calcd *m/z* 1010.5894, found 1010.5878), y_9(2+) (calcd *m/z* 514.3116, found 514.3127), y_{10}(2+) (calcd *m/z* 587.8458, found 587.8457), y_{11}(2+) (calcd *m/z* 652.3671, found 652.3664), **HPLC**: R_t= 5.73 min (gradient 2), **yield:** 60%, 23 mg

7.7 Preparation of peptide with C-terminal hydrazide H-Gly-Thr-Phe-Thr-Ser-Asp-Val-Ser-Ser-Tyr-Leu-Glu-Gly-Gln-Ala-$NHNH_2$

The synthesis of the peptide with C-terminal hydrazide was performed on solid support according to the standard Fmoc strategy and using Chemmatrix Trityl-OH resin. The resin was swollen in DCM for 30 min followed by the addition of freshly prepared 10% $SOCl_2$/DCM. The syringe was left on the rotator overnight. The next day the resin was washed with 3x DCM, 2x 5%DIPEA/DCM, and then left on the rotator in DMF for 20 minutes. After this time the resin was loaded with the mixture of 10% hydrazine in DMF for 2 hours. The resin was then washed with DMF (3x), 5% DIPEA/DMF (1x), and DMF (2x). In the next step, the resin was loaded with the mixture of Fmoc-Lys(Boc)-OH (3 eq) and DIPEA (6 eq) for 2 hours. For Fmoc deprotection, 25% PIP/DMF was added and the syringe was placed in an ultrasonic bath for 3 minutes. For coupling of additional amino acid residues, the following procedure was used: Fmoc-protected amino acid (3 eq), PyBOP (3 eq), and DIPEA (6 eq) were dissolved in DMF and placed in a syringe reactor for 20 min assisted by ultrasound, followed by filtration and 5x resin washing with DMF. The resin was then filtered and washed 7 times with DMF. The Kaiser test was performed to check the completeness of the couplings. The above steps were repeated with subsequent amino acid residues until the desired sequence was obtained. Finally, the resin was shrunk with the following combination of solvents: DMF/DCM, DCM, DCM/THF, THF, THF/Et_2O, Et_2O, and then dried overnight in a desiccator. The peptide was eluted from the resin with a standard TFA/H_2O/TIS mixture (95:2.5:2.5) within 2 hours. The crude

peptide was then precipitated with cold diethyl ether and centrifuged, and purified by preparative-scale RP-HPLC.

HR-ESI-MS calcd *m/z* 1575.7284, found 1575.7257 $[M+H]^{1+}$, *m/z* 788.3678, found 788.3657 $[M+2H]^{2+}$; calcd *m/z* 525.9143, found 525.9150 $[M+3H]^{3+}$; **ESI-MS/MS** (*m/z* 788.3660, CE 30eV, $[M+2H]^{2+}$): b_4 (calcd *m/z* 407.1925, found 407.1958), b_5 (calcd *m/z* 494.2245, found 494.2258), b_6 (calcd *m/z* 609.2515, found 609.2512), b_7 (calcd *m/z* 708.3199, found 708.3178), b_{10} (calcd *m/z* 1045.4473, found 1045.4443), b_{11} (calcd *m/z* 1158.5313, found 1158.5298), b_{12} (calcd *m/z* 1287.5739, found 1287.5731), b_{13} (calcd *m/z* 1344.5954, found 1344.5947), b_{14} (calcd *m/z* 1472.6540, found 1472.6536), y_3 (calcd *m/z* 289.1617, found 289.1688), y_5 (calcd *m/z* 531.2884, found 531.2886), y_8 (calcd *m/z* 868.4157, found 868.4134), y_9 (calcd *m/z* 967.4842, found 967.4819), y_{10} (calcd *m/z* 1082.5111, found 1082.5083); **HPLC**: R_t= 6.18 min (gradient 2), **yield:** 62%, 25 mg

8 General procedure for desulfurization of cysteinyl peptides

The peptide (0.2 mg) and TCEP x HCl (1mg) were placed in an HPLC glass vial (2 ml, transparent borosilicate glass) and dissolved in 200 µl of aqueous buffer consisting of 8M guanidine hydrochloride and 0.2M Na_2HPO_4 (the reverse osmosis system deionized water; Hydrolab, Poland). After the adjustment of pH to 5 with 4M HCl (or 4M NaOH if more TCEP was added), 0.05 - 0.1eq of Rose Bengal was added as an aqueous solution (freshly prepared stock solution 2 mg/ml can be used for around 1 week, but the long-term stability of the solution has not been tested). Then, the sample was irradiated using an office lamp (Kobi Light Sp.z.o.o, No.KX3087, 230V, 50Hz) with an LED bulb (Philips, 6500K, the spectrum presented below) for over 3h (The vial was placed horizontally and the bulb approximated at a distance of 0.5-1cm). During this time, a change in the color of the solution is usually observed. An aliquot of the obtained mixture was taken, diluted with water, and analyzed by LC-MS.

*Additional guidance: If the Cys → Ala transformation shows poor performance, then other pHs should be checked, in particular, pH 7. pH 5 is the optimum value that will work for most peptides, while the specificity of some peptide sequences requires a higher pH. A second factor affecting the success of the reaction is the absence of scavengers (desulfurization proceeds through radical reaction mechanism), especially of selenium compounds. In such a situation, they must either be extracted from the aqueous solution

(if relatively abundant) or the Rose Bengal equivalent must be increased to as much as 0.3-0.4 eq.

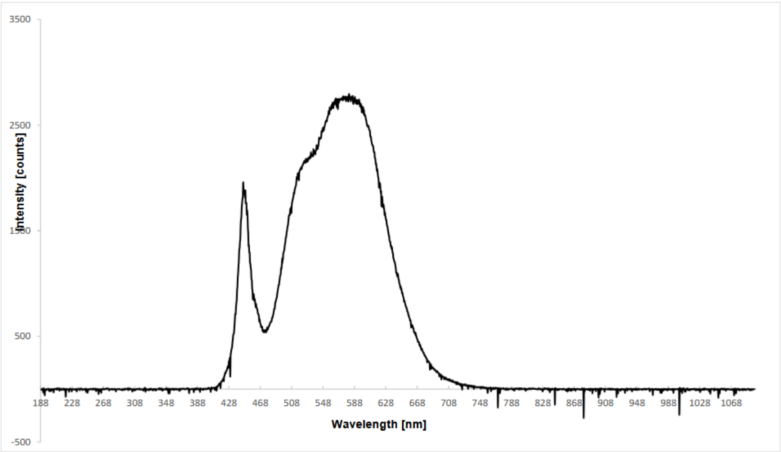

9. *One-pot native chemical ligation (NCL)-desulfurization*

Peptide with C-terminal hydrazide H-Gly-Thr-Phe-Thr-Ser-Asp-Val-Ser-Ser-Tyr-Leu-Glu-Gly-Gln-Ala-$NHNH_2$ (0.5 mg) and 2 mg of TCEP were placed in the Eppendorf tube and dissolved in 200 µl of aqueous buffer consisting of 8M guanidine hydrochloride and 0.2M Na_2HPO_4. The pH was adjusted to 3 with 4M HCl. Then, 0.9 mg of diphenyl diselenide (DPDS) was dissolved in 20 µl of NMP or DMF and added to the reaction mixture followed by the addition of 3 µl of acetylacetone. The obtained mixture was sonicated for 30 seconds. The Eppendorf tube was then placed on a heater (40 ^oC) for 2.5 hours. During this time, a C-terminal selenoester was formed. In the next step, the peptide H-Cys-Lys-Glu-Phe-Ile-Ala-Trp-Leu-Val-Arg-Gly-Arg-Gly-OH (0.56 mg) was added and the pH was adjusted to 7 with a 4M NaOH. The Eppendorf tube was again placed in a heater (40 ^oC) for 40 min. After this time, the mixture was acidified and extracted with diethyl ether (3x) to remove the excessive DPDS. Then 2 mg of TCEP was added, pH adjusted to 7, and 0.3 eq of Rose Bengal was added. The mixture was transferred to a glass HPLC vial and exposed to LED light for 3h. Aliquot of the sample was taken and diluted with 10% ACN in H_20 and analyzed by LCMS.

10. *Desulfurization of peptide H-Thr-Gly-Cys-Ala-Phe-Lys-NH_2 in D_2O.*

The peptide (0.2 mg) and TCEP x HCl (1mg) were placed in an HPLC glass vial and dissolved in 200 µl of D_2O containing 0.2M Na_2HPO_4. The pH value obtained was in the region of 6 and was not altered to avoid introducing additional amounts of non-isotopically labeled water. In the next step, 0.05 - 0.1eq of Rose Bengal was added as an aqueous solution in D_2O (2 mg/ml). Then, the sample was irradiated using an office lamp with an LED bulb (6500K) for over 3h. Finally, the mixture was desalted using OMIX C18 tipp and lyophilized. 30 min before LCMS analysis, the peptide was dissolved in water containing 0.1% HCOOH.

11. The procedure for desulfurization of proteins

The protein (1 mg) and TCEP x HCl (5 mg) were placed in an HPLC glass vial and dissolved in 200 µl of aqueous buffer consisting of 6M guanidine hydrochloride (Gd x HCl) and 0.2M Na_2HPO_4. After the adjustment of pH to 7 with 4M NaOH, the vial was placed on a heater (40 ^{o}C) for 45 min to denature the protein and reduce disulfide bridges. Then, 50 µl of Rose Bengal was added as an aqueous solution (2 mg/ml). The sample was irradiated using an office lamp with an LED bulb (6500K) for over 3h.

12. Protein tryptic digestion

50 µl of protein (lysozyme C, α-crystallin, papain) from the desulfurization experiment was taken and diluted to 300 µl with deionized water, thus the final concentration of Gd x HCl was 1M. After the adjustment of pH to 8, 8 µl (1 : 20) of trypsin solution (1 mg/ml) was added. The sample was incubated at 37 ^{o}C overnight. Finally, 5 µl of concentrated HCOOH was added to stop the digestion. The obtained samples were analyzed by RP-UPLC-MS.

13. Bioinformatics analysis

LC-MS/MS analyzes of the tryptic digests were performed on a Shimadzu 9030 (QTof) instrument using the automated fragmentation mode – DDA and collision energy of 20 eV ±5. The obtained results were subjected to bioinformatics analysis. We used LabSolution software to convert .lc data files into mzML format. The data were analyzed using the PEAKS search engine. For this purpose, we downloaded the FASTA file from the UniProt database for the analyzed proteins. The following search parameters were set:

precursor mass error tolerance ±0.1 Da; fragment ion tolerance ±0.2 Da; CID fragmentation, semi-specific digestion mode by trypsin, assuming up to 2 missed cleavages per peptide. Deamidation, oxidation of methionine, and desulfurization modifications were set as variable PTMs, and a maximum of 2 variable PTMs were allowed per peptide. A false discovery rate (FDR) of <2% at the spectral level was used to filter search results. In addition, the presence of at least 2 unique peptides was required for protein identification.

14. Results

14.1 Synthesis of model peptide H-Thr-Gly-Cys-Ala-Phe-Lys-NH_2

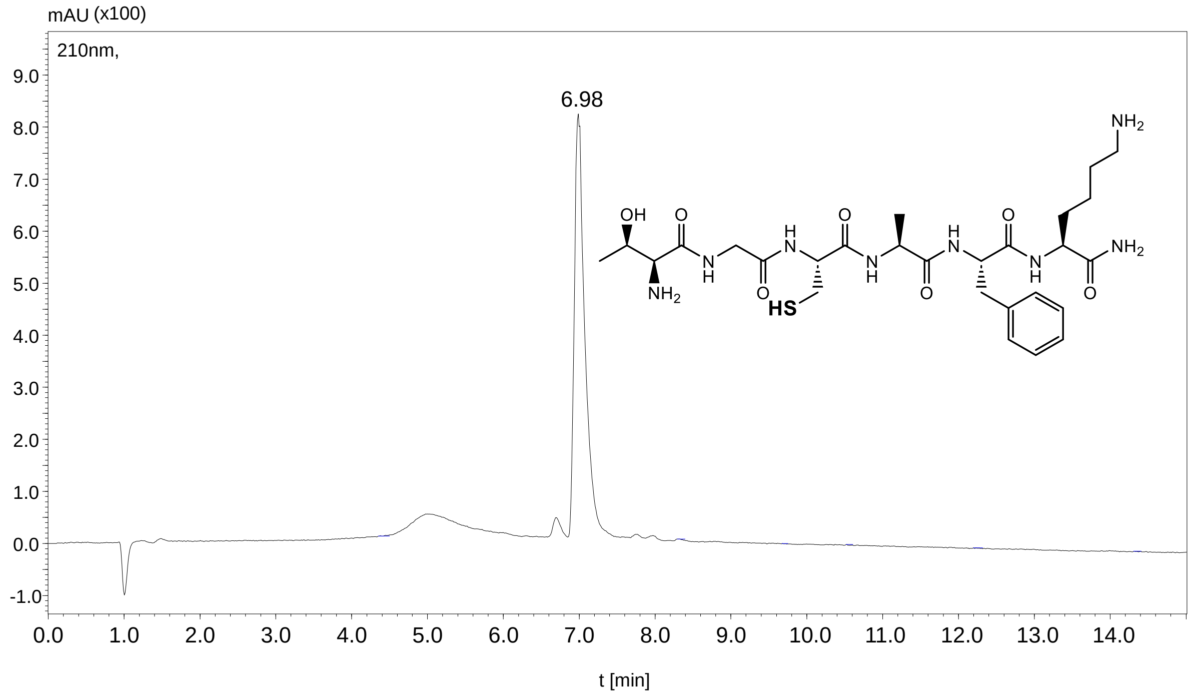

Fig S 1. HPLC chromatogram obtained for model peptide H-Thr-Gly-Cys-Ala-Phe-Lys-NH_2.

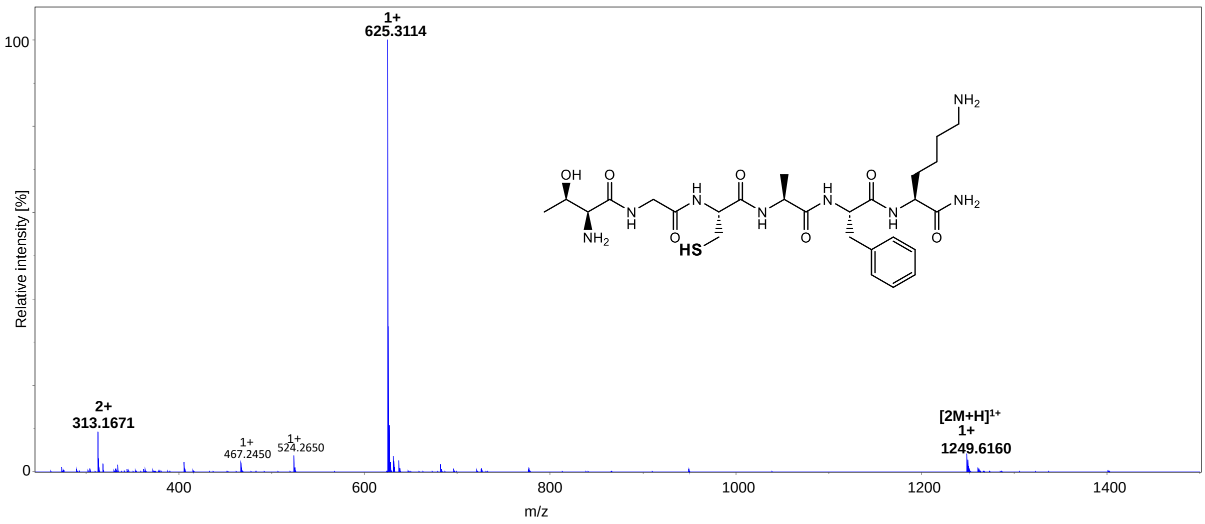

Fig S 2. ESI-MS spectrum obtained for model peptide H-Thr-Gly-Cys-Ala-Phe-Lys-NH2.

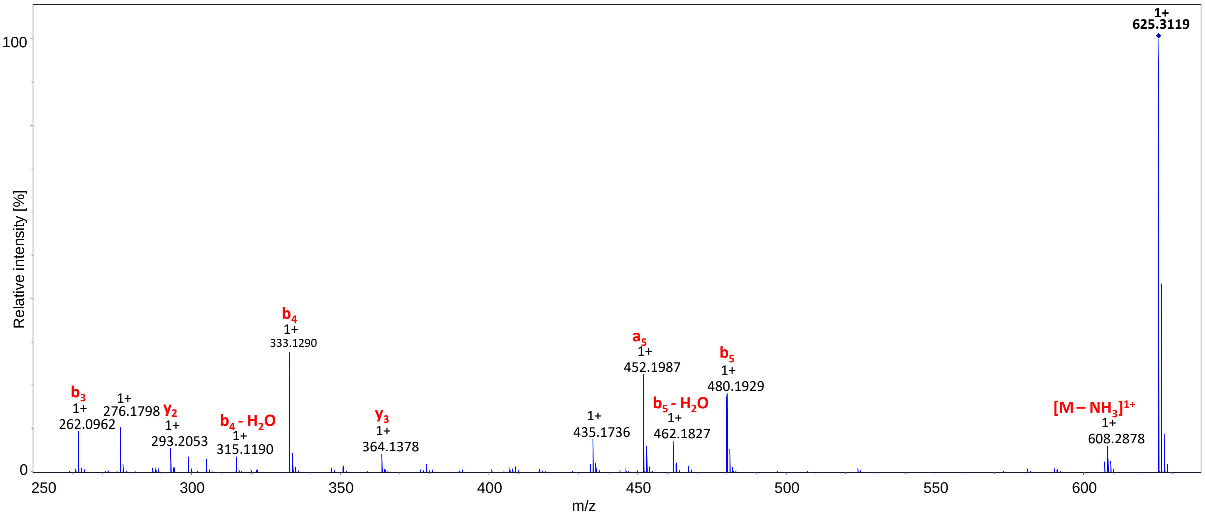

Fig S 3. ESI-MS/MS (CE 25eV) spectrum obtained for model peptide H-Thr-Gly-Cys-Ala-Phe-Lys-NH2.

14.2 Optimization of desulfurization – addition of external thiol MESNa

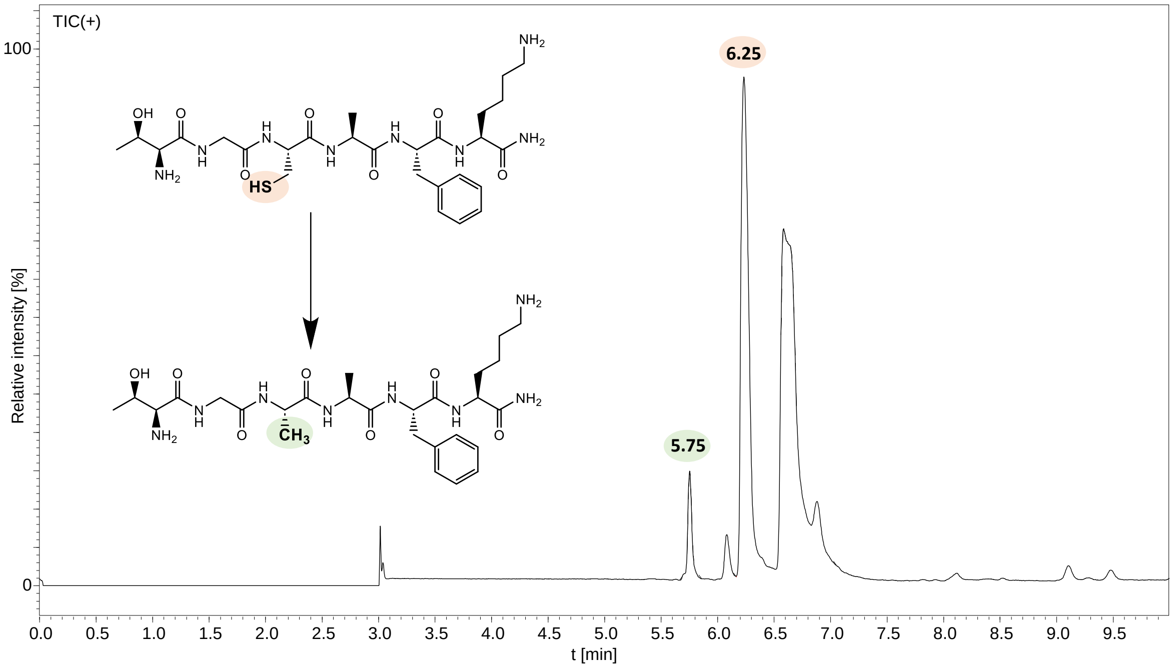

Fig S 4. LC-MS chromatogram (TIC) obtained after desulfurization of H-Thr-Gly-Cys-Ala-Phe-Lys-NH_2 (0.2 eq Rose Bengal, 5 mg TCEP, pH 5, 20 mg MESNa)

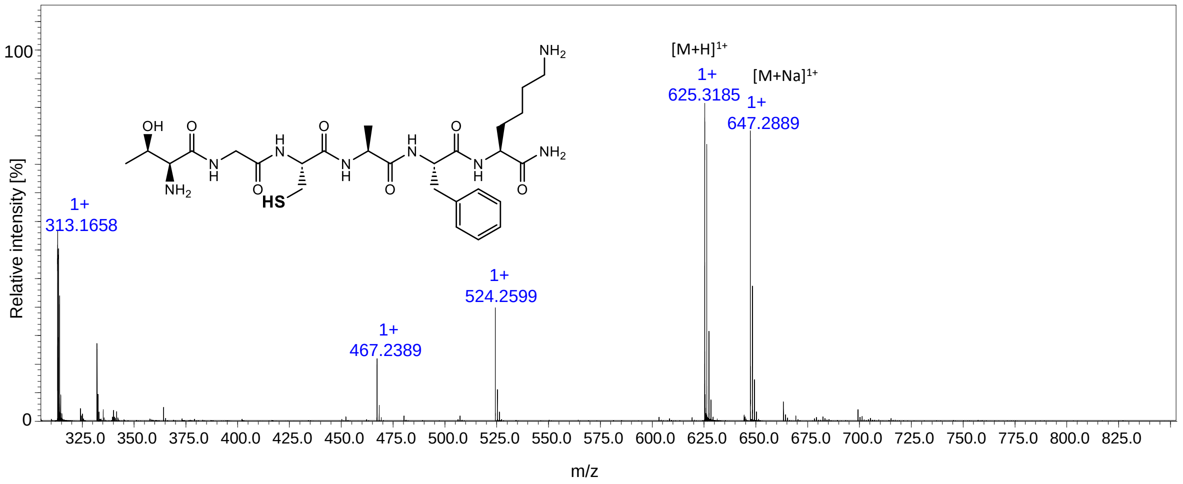

Fig S 5. LC-MS spectrum obtained for peptide H-Thr-Gly-Cys-Ala-Phe-Lys-NH_2 (substrate) at 6.25 min.

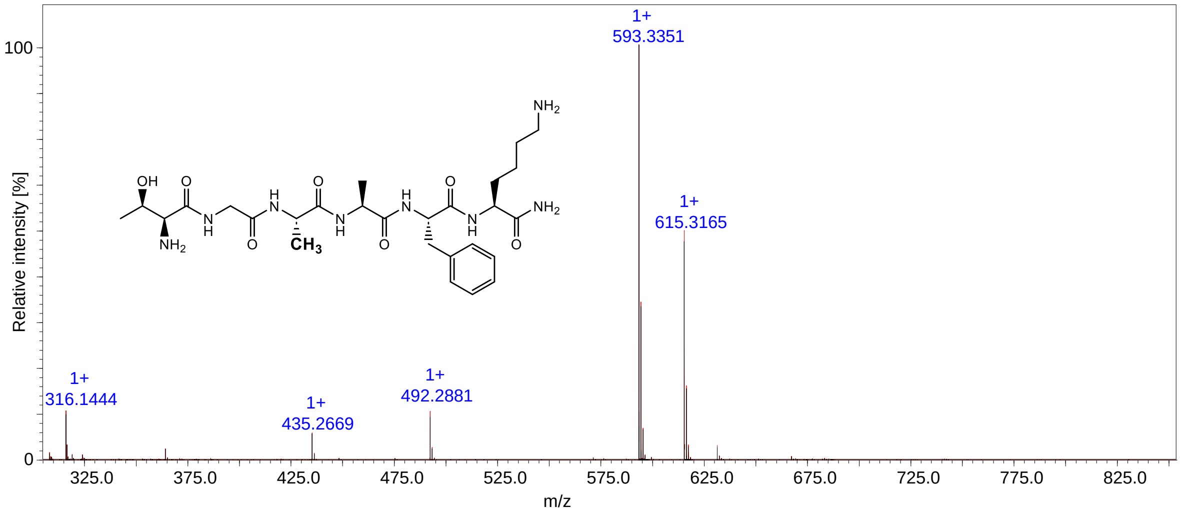

Fig S 6. LC-MS spectrum obtained for peptide H-Thr-Gly-Cys-Ala-Phe-Lys-NH_2 (desulfurization product) at 5.75 min.

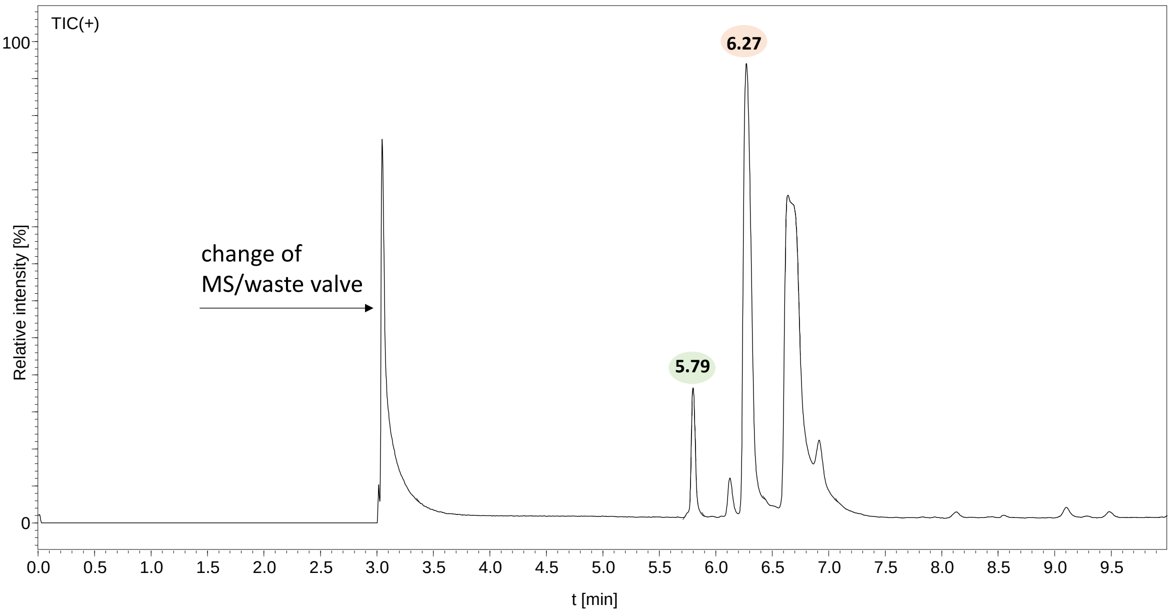

Fig S 7. LC-MS chromatogram (TIC) obtained after desulfurization of H-Thr-Gly-Cys-Ala-Phe-Lys-NH_2 (0.2 eq Rose Bengal, 5 mg TCEP, pH 5,15 mg MESNa).

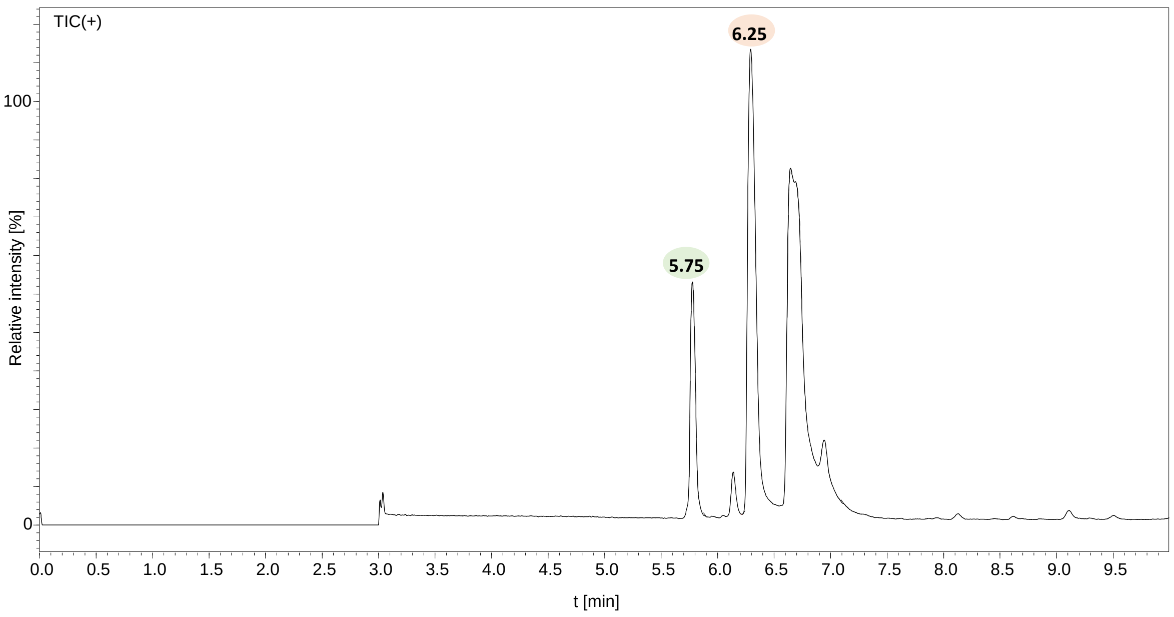

Fig S 8. LC-MS chromatogram (TIC) obtained after desulfurization of H-Thr-Gly-Cys-Ala-Phe-Lys-NH_2 (0.2 eq Rose Bengal, 5 mg TCEP, pH 5, 10 mg MESNa).

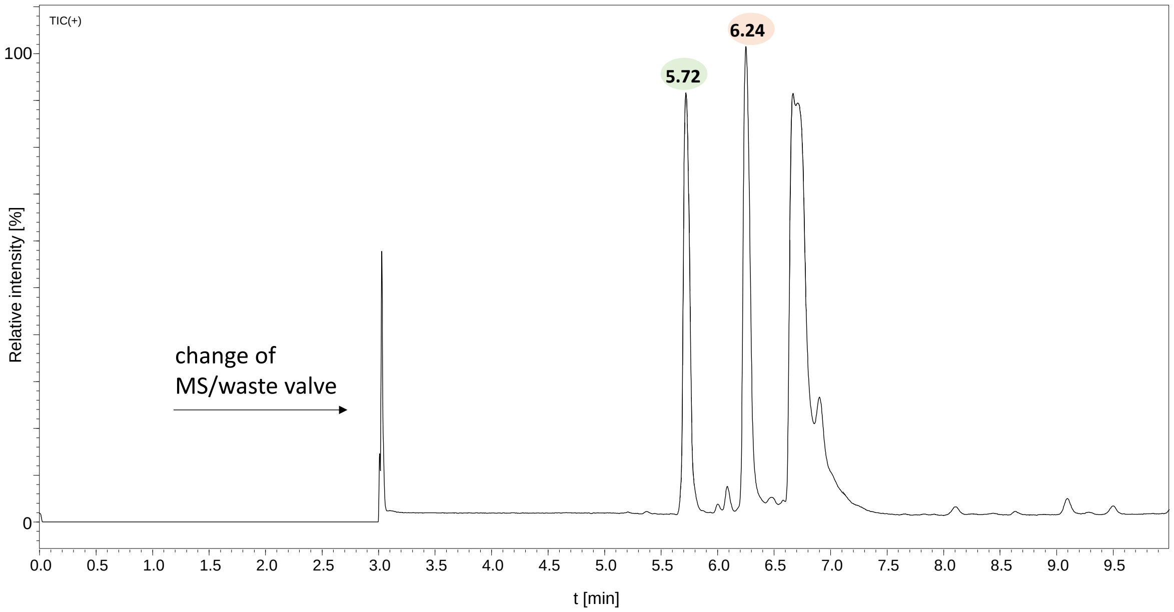

Fig S 9. LC-MS chromatogram (TIC) obtained after desulfurization of H-Thr-Gly-Cys-Ala-Phe-Lys-NH_2 (0.2 eq Rose Bengal, 5 mg TCEP, pH 5, 5 mg MESNa).

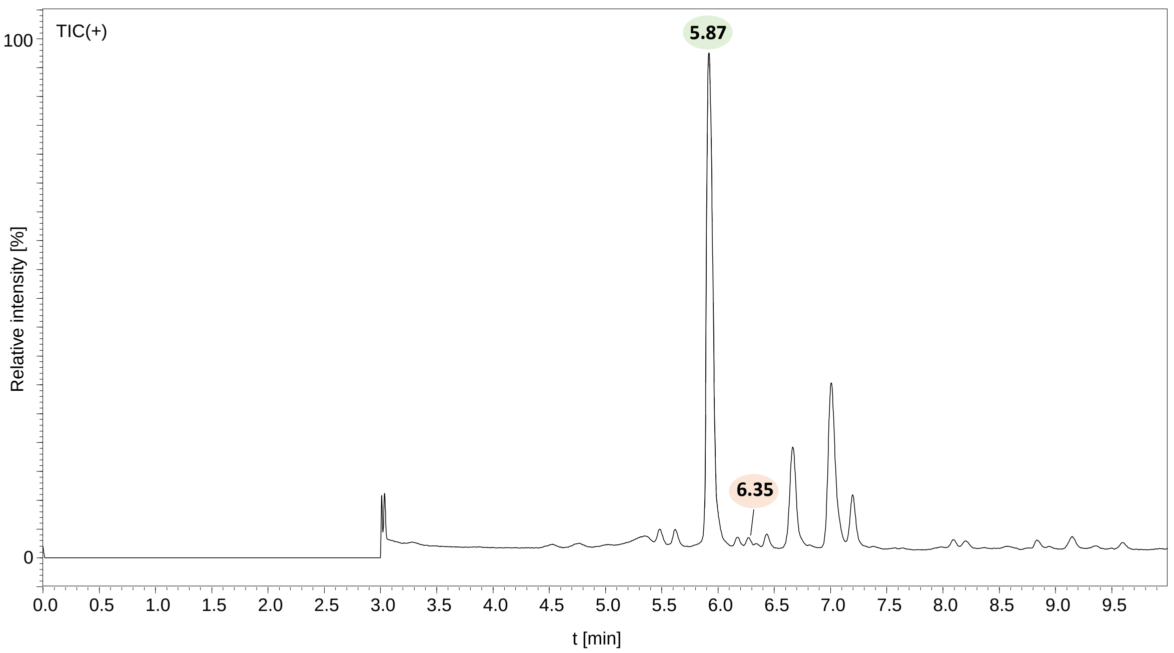

Fig S 10. LC-MS chromatogram (TIC) obtained after desulfurization of H-Thr-Gly-Cys-Ala-Phe-Lys-NH_2 (0.2 eq Rose Bengal, pH 5, 5 mg TCEP, without MESNa).

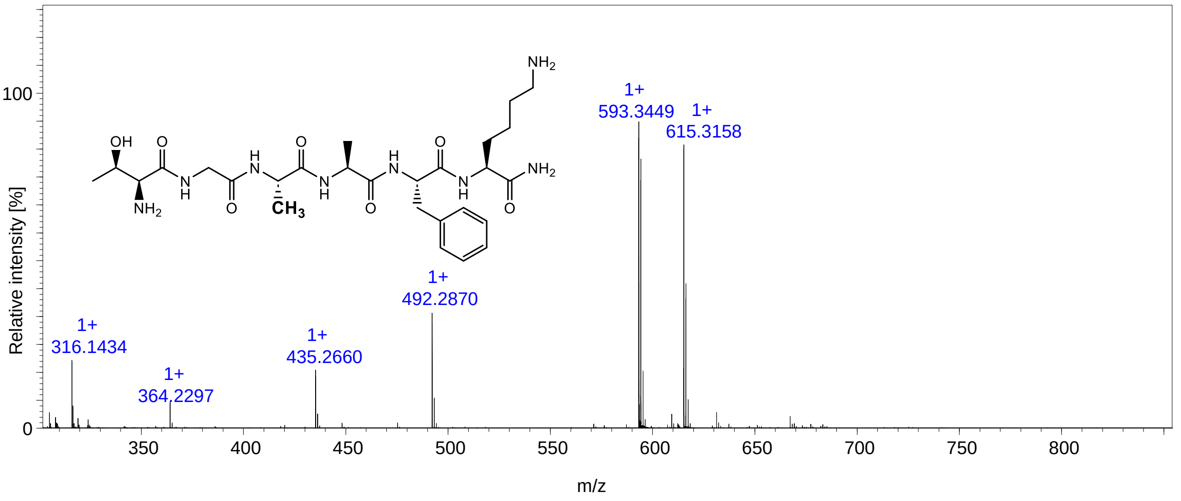

Fig S 11. LC-MS spectrum obtained for peptide H-Thr-Gly-Cys-Ala-Phe-Lys-NH_2 (desulfurization product) at 5.87 min (*m/z* 593.3449 $[M+H]^{1+}$; *m/z* 615.3158 $[M+Na]^{1+}$).

14.3 Optimization of desulfurization – addition of TCEP

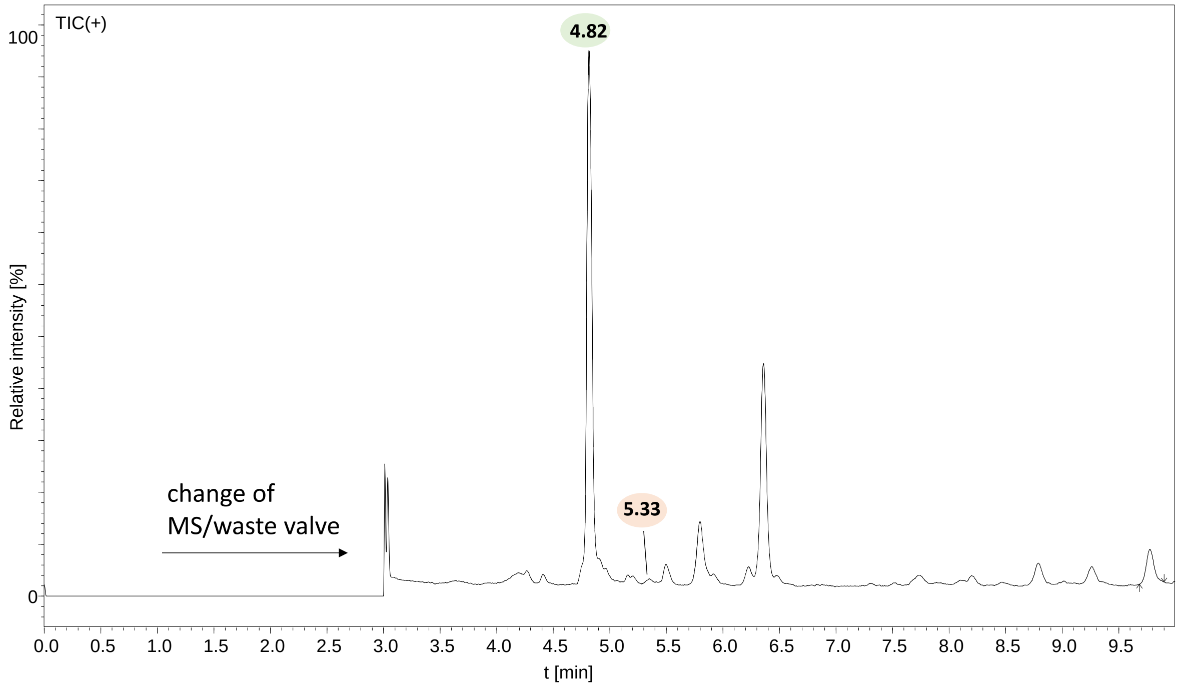

Fig S 12. LC-MS chromatogram (TIC) obtained after desulfurization of H-Thr-Gly-Cys-Ala-Phe-Lys-NH_2 (0.2 eq Rose Bengal, pH 5, 10 mg TCEP)

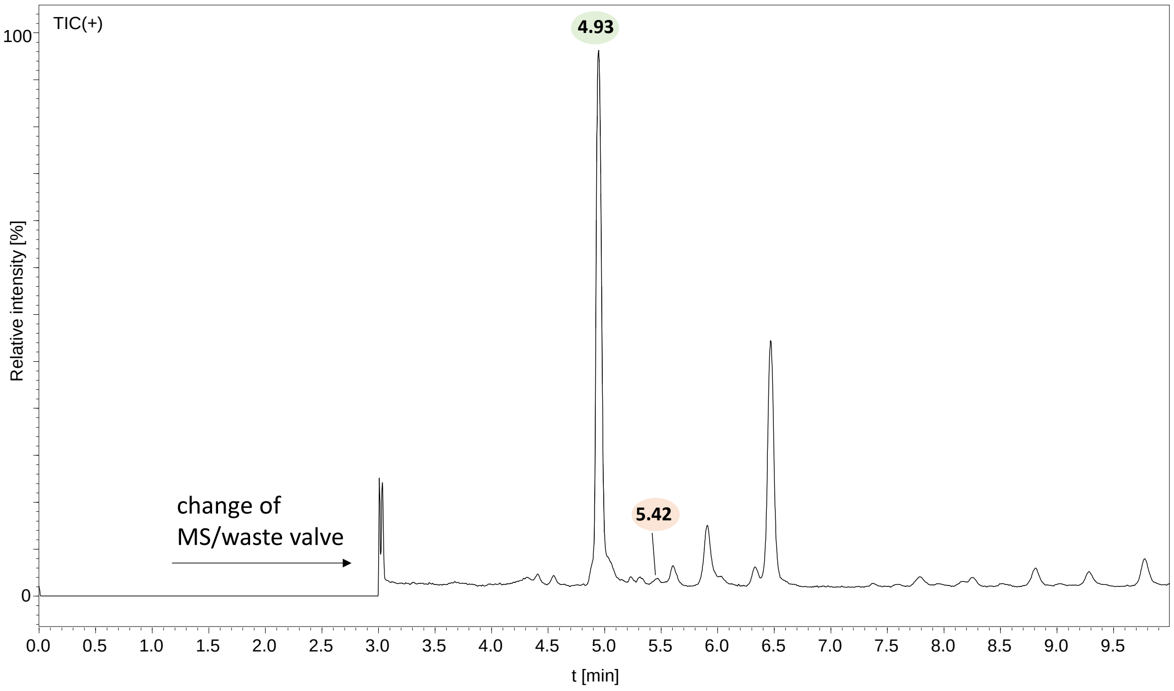

Fig S 13. LC-MS chromatogram (TIC) obtained after desulfurization of H-Thr-Gly-Cys-Ala-Phe-Lys-NH_2 (0.2 eq Rose Bengal, pH 5, 5 mg TCEP).

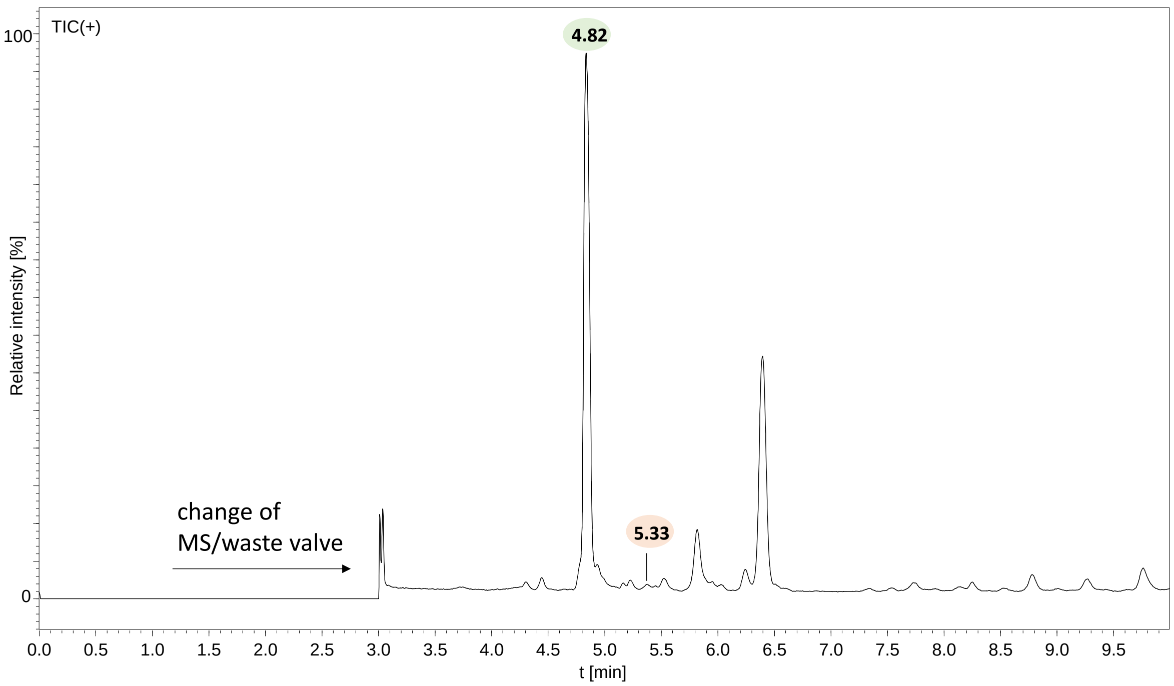

Fig S 14. LC-MS chromatogram (TIC) obtained after desulfurization of H-Thr-Gly-Cys-Ala-Phe-Lys-NH_2 (0.2 eq Rose Bengal, pH 5, 2 mg TCEP).

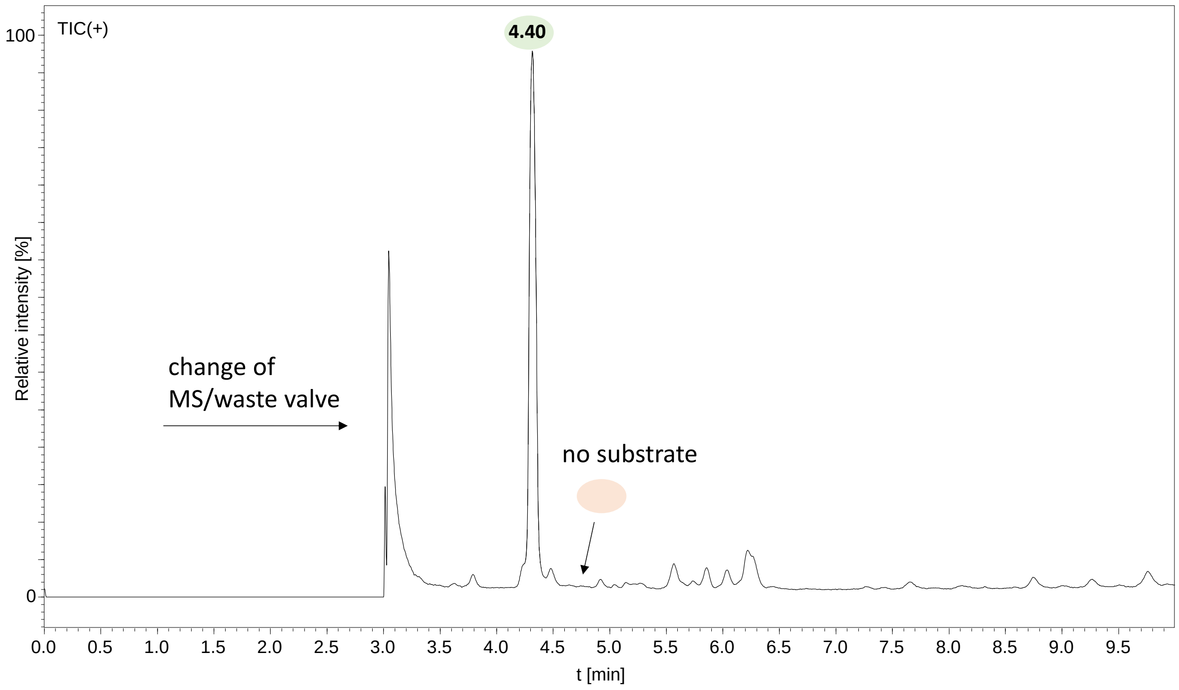

Fig S 15. LC-MS chromatogram (TIC) obtained after desulfurization of H-Thr-Gly-Cys-Ala-Phe-Lys-NH_2 (0.2 eq Rose Bengal, pH 5, 1 mg TCEP).

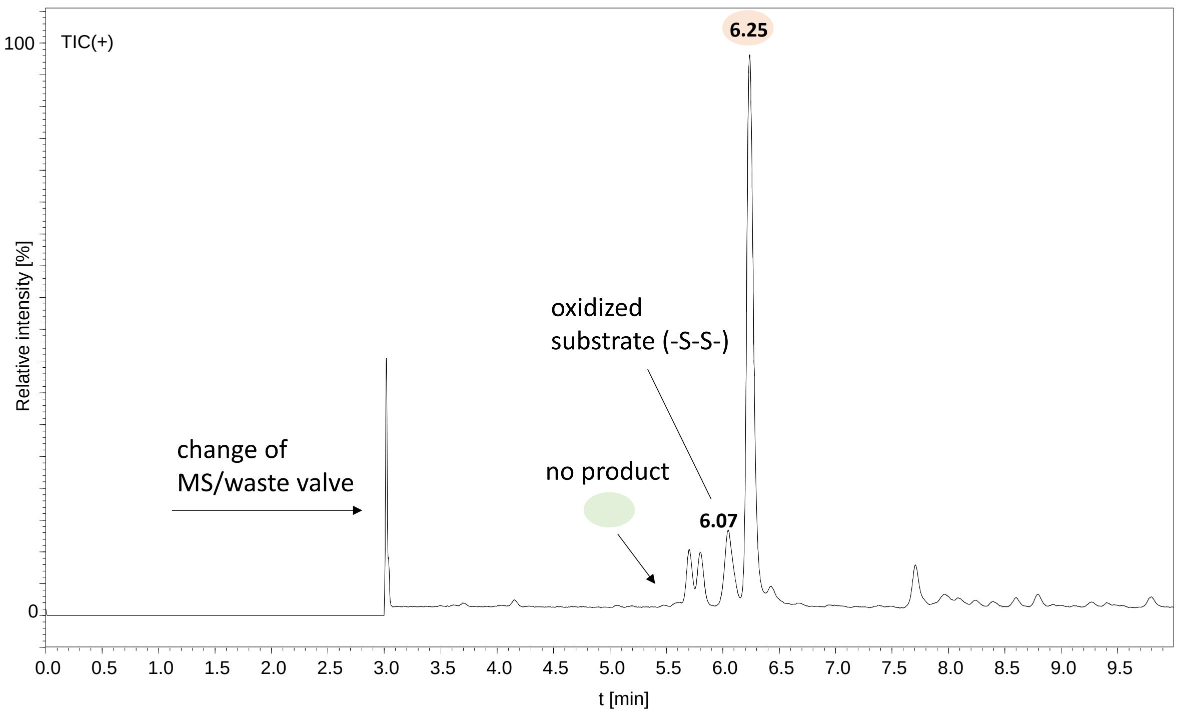

Fig S 16. LC-MS chromatogram (TIC) obtained after desulfurization of H-Thr-Gly-Cys-Ala-Phe-Lys-NH_2 (0.2 eq Rose Bengal, pH 5, without TCEP).

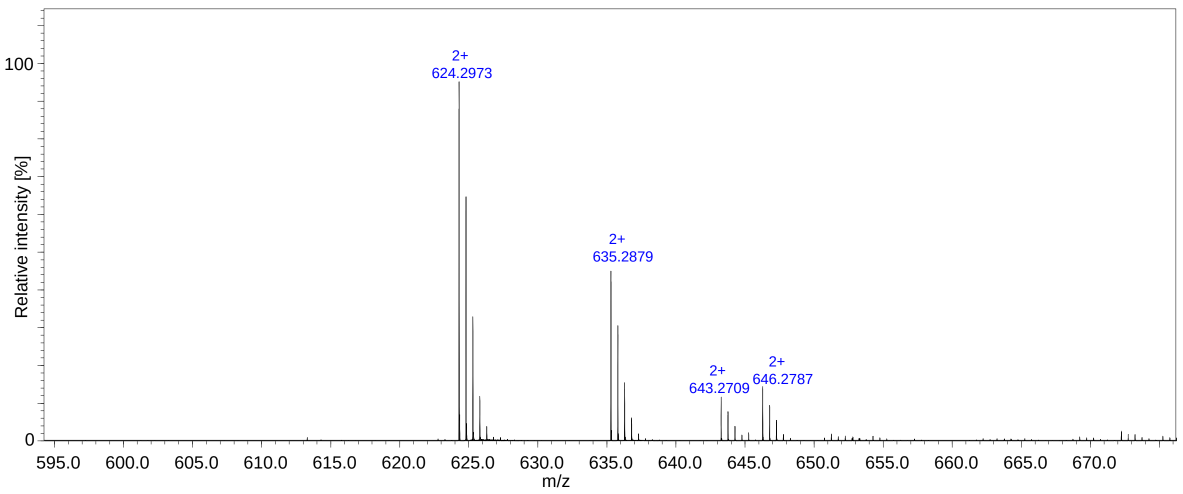

Fig S 17. LC-MS spectrum obtained for peptide H-Thr-Gly-Cys-Ala-Phe-Lys-NH_2 after desulfurization procedure. MS spectrum at 6.07 min (*m/z* 624.2973 $[M+H]^{2+}$; *m/z* 635.2879 $[M+Na]^{2+}$)

14.4 Optimization of desulfurization – addition of Rose Bengal

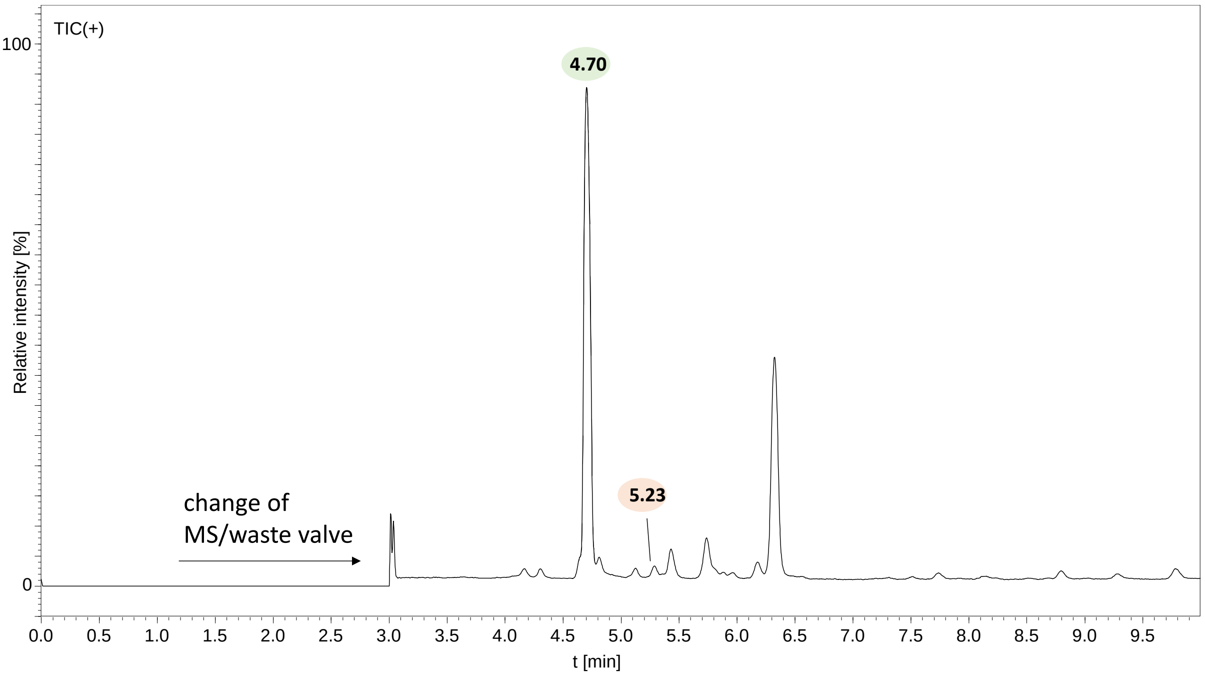

Fig S 18. LC-MS chromatogram (TIC) obtained after desulfurization of H-Thr-Gly-Cys-Ala-Phe-Lys-NH_2 (0.1 eq Rose Bengal, pH 5, 1 mg TCEP).

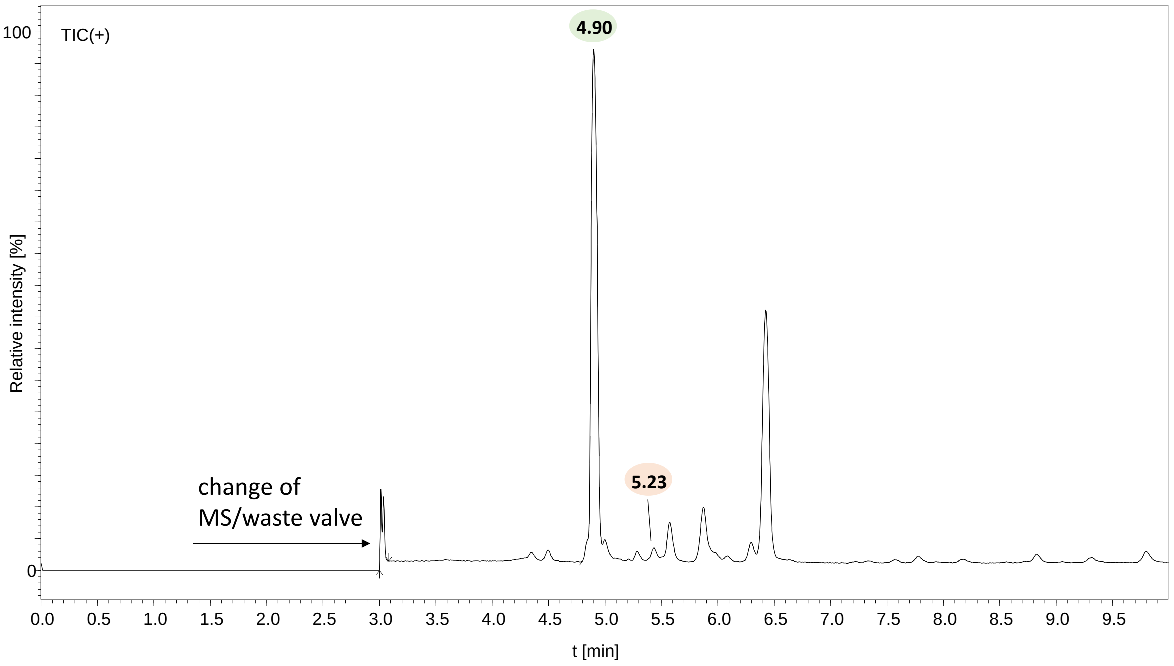

Fig S 19. LC-MS chromatogram (TIC) obtained after desulfurization of H-Thr-Gly-Cys-Ala-Phe-Lys-NH_2 (0.05 eq Rose Bengal, pH 5, 1 mg TCEP).

14.5 Optimization of desulfurization – change of pH

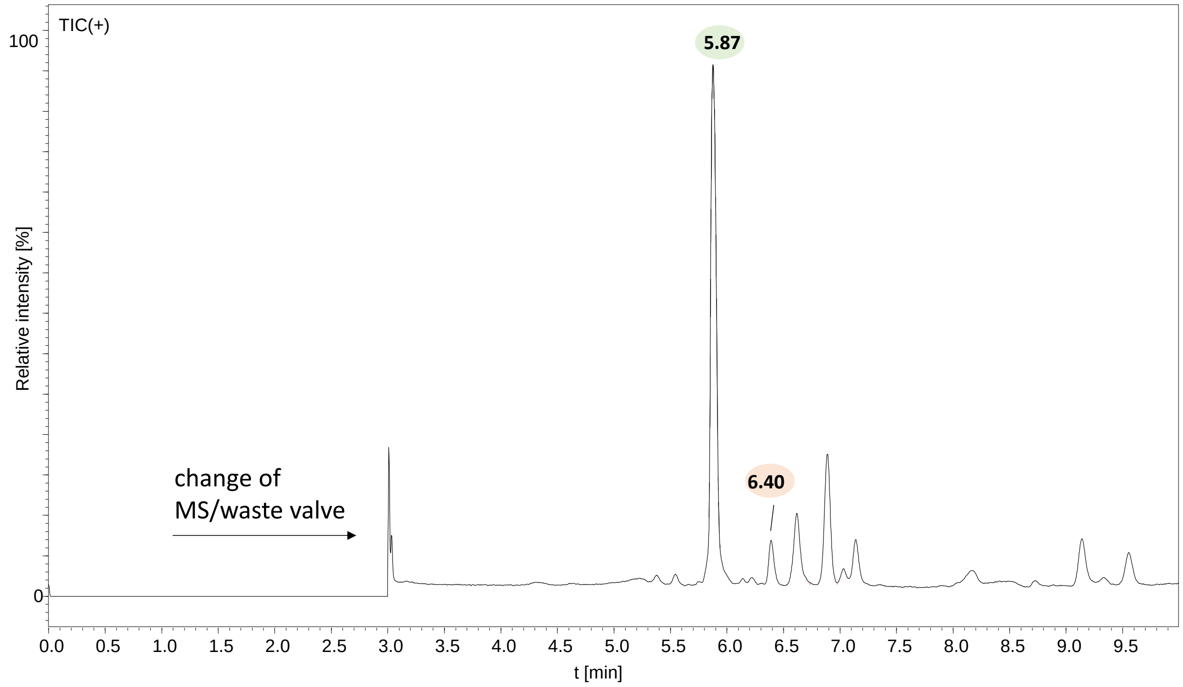

Fig S 20. LC-MS chromatogram (TIC) obtained after desulfurization of H-Thr-Gly-Cys-Ala-Phe-Lys-NH_2 (0.1 eq Rose Bengal, pH 5, 1 mg TCEP, pH 4).

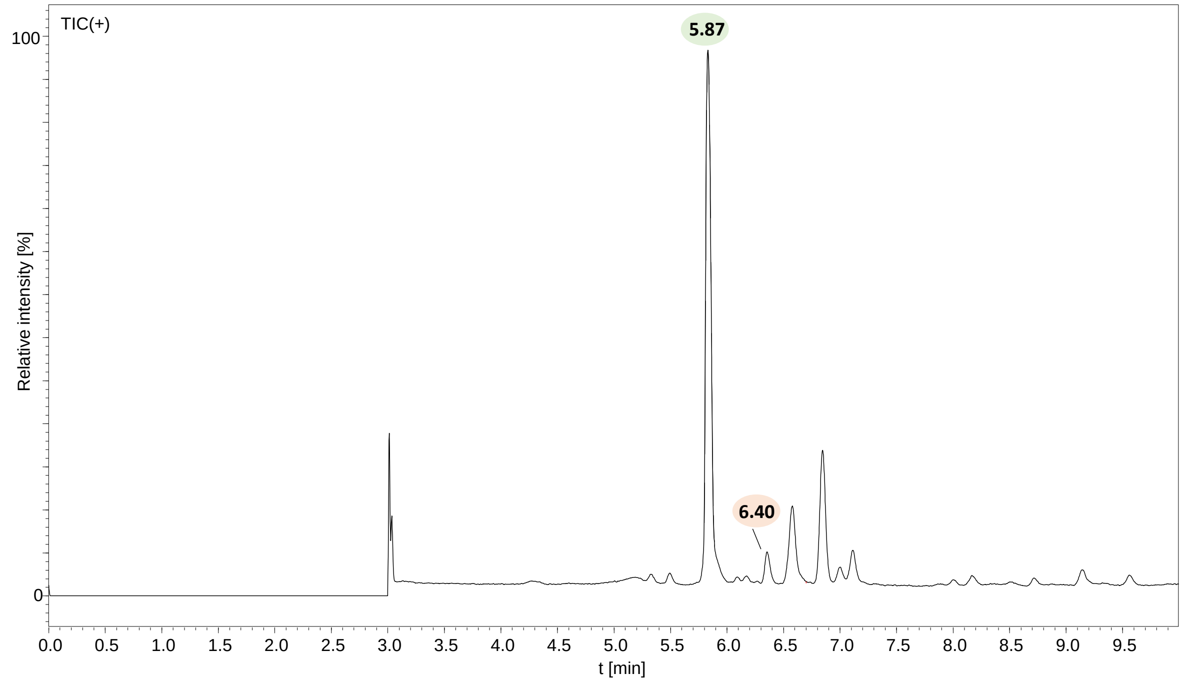

Fig S 21. LC-MS chromatogram (TIC) obtained after desulfurization of H-Thr-Gly-Cys-Ala-Phe-Lys-NH_2 (0.1 eq Rose Bengal, 1 mg TCEP, pH 5).

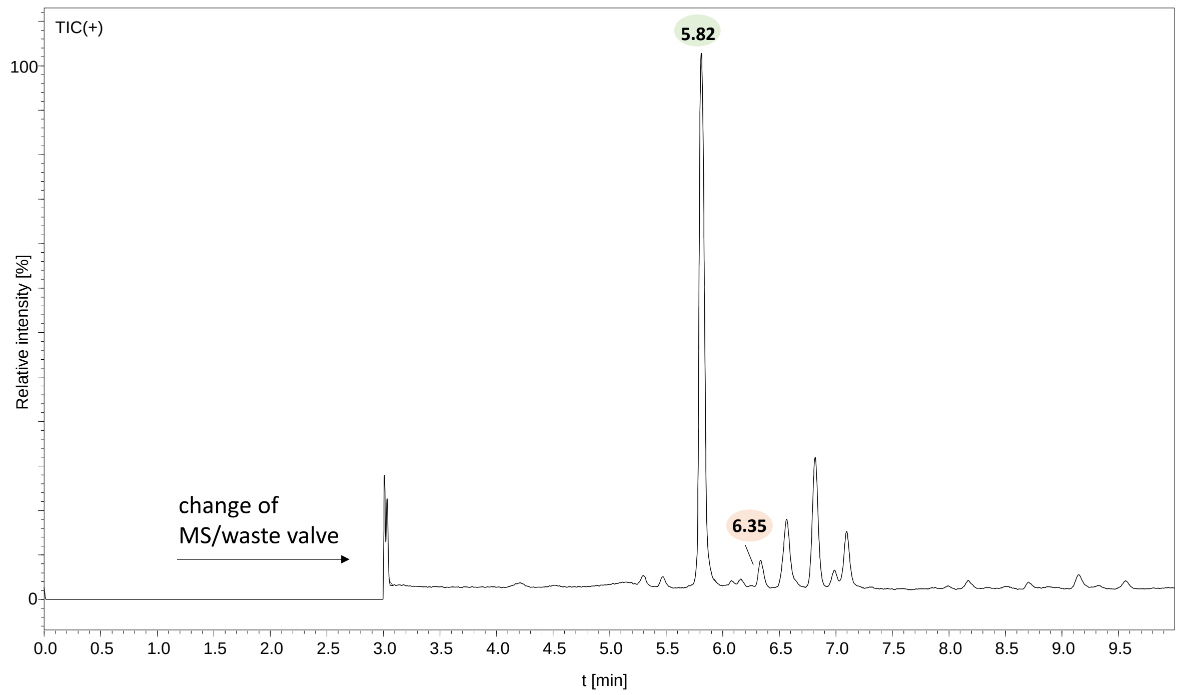

Fig S 22. LC-MS chromatogram (TIC) obtained after desulfurization of H-Thr-Gly-Cys-Ala-Phe-Lys-NH_2 (0.1 eq Rose Bengal, 1 mg TCEP, pH 6).

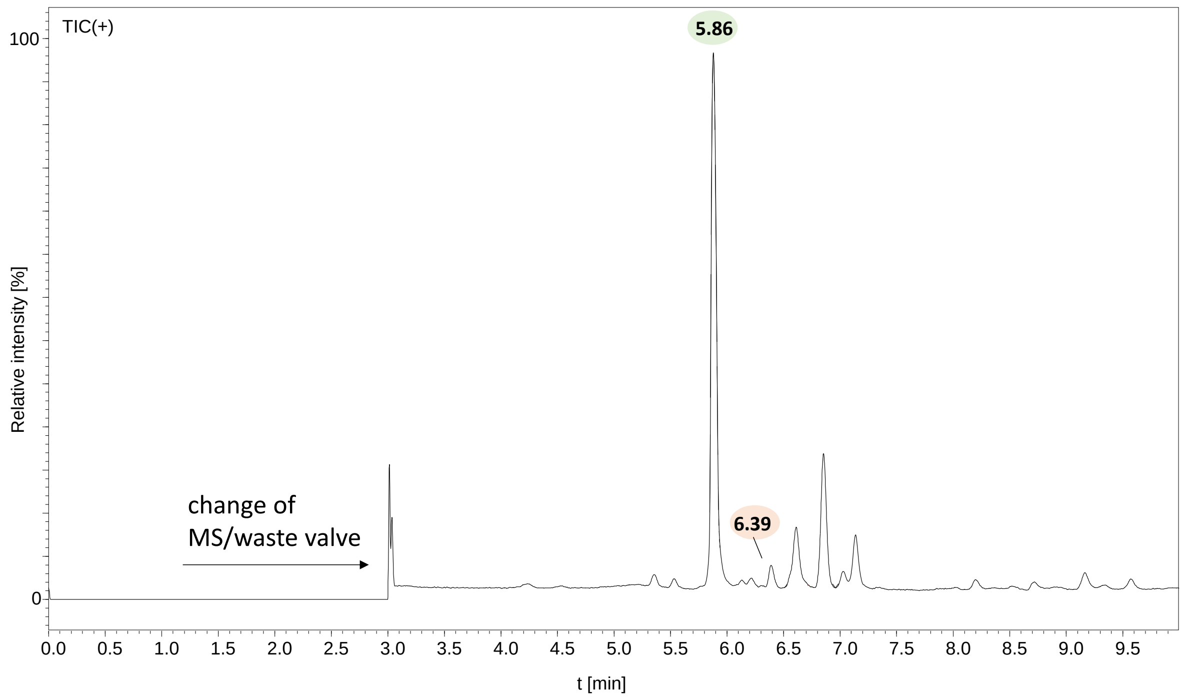

Fig S 23. LC-MS chromatogram (TIC) obtained after desulfurization of H-Thr-Gly-Cys-Ala-Phe-Lys-NH_2 (0.1 eq Rose Bengal, 1 mg TCEP, pH 7).

14.6 Optimization of desulfurization – time of exposure to visible light

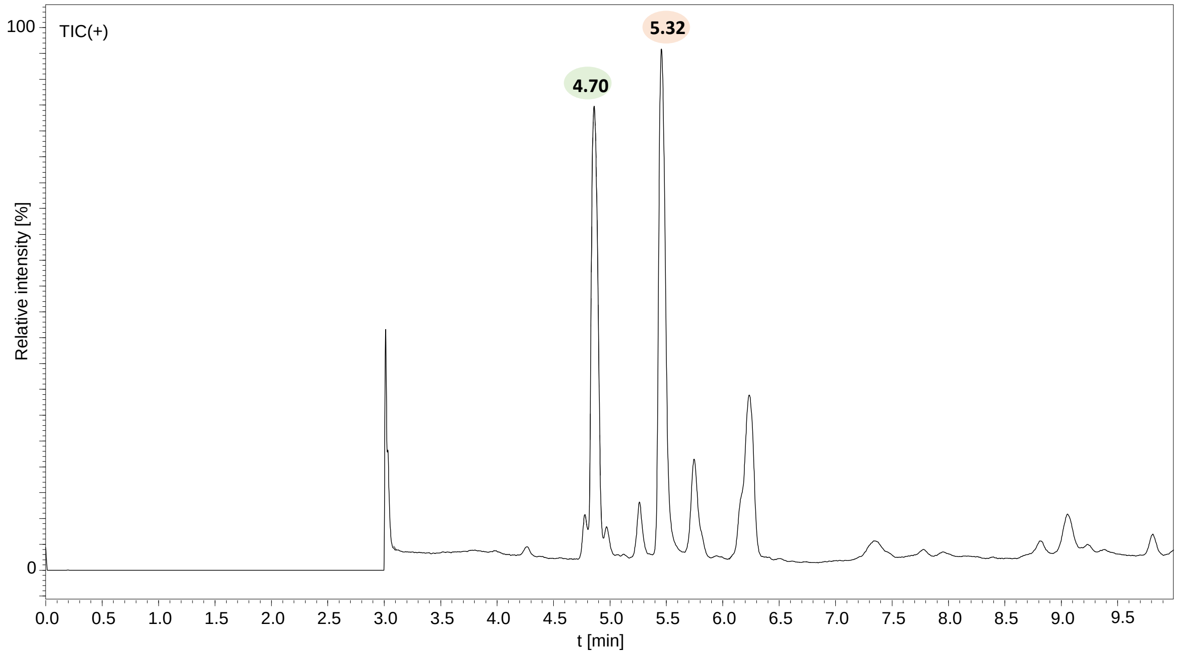

Fig S 24. LC-MS chromatogram (TIC) obtained after desulfurization of H-Thr-Gly-Cys-Ala-Phe-Lys-NH_2 (0.1 eq Rose Bengal, 1 mg TCEP, pH 5, 30 min)

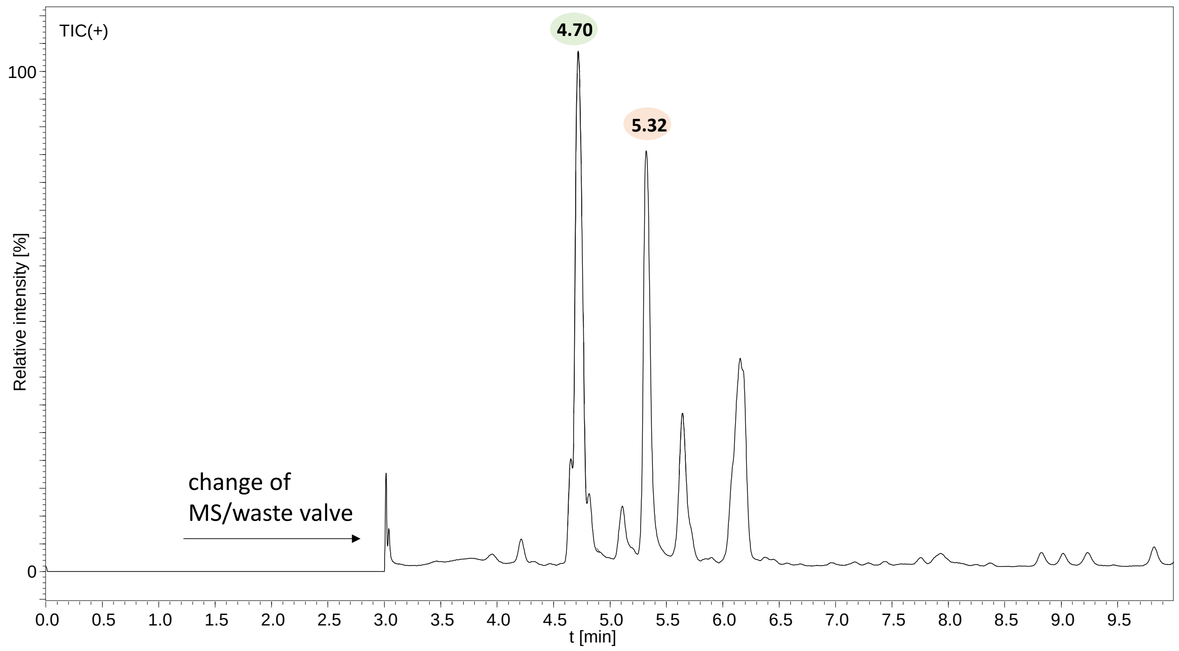

Fig S 25. LC-MS chromatogram (TIC) obtained after desulfurization of H-Thr-Gly-Cys-Ala-Phe-Lys-NH_2 (0.1 eq Rose Bengal, 1 mg TCEP, pH 5, 60 min)

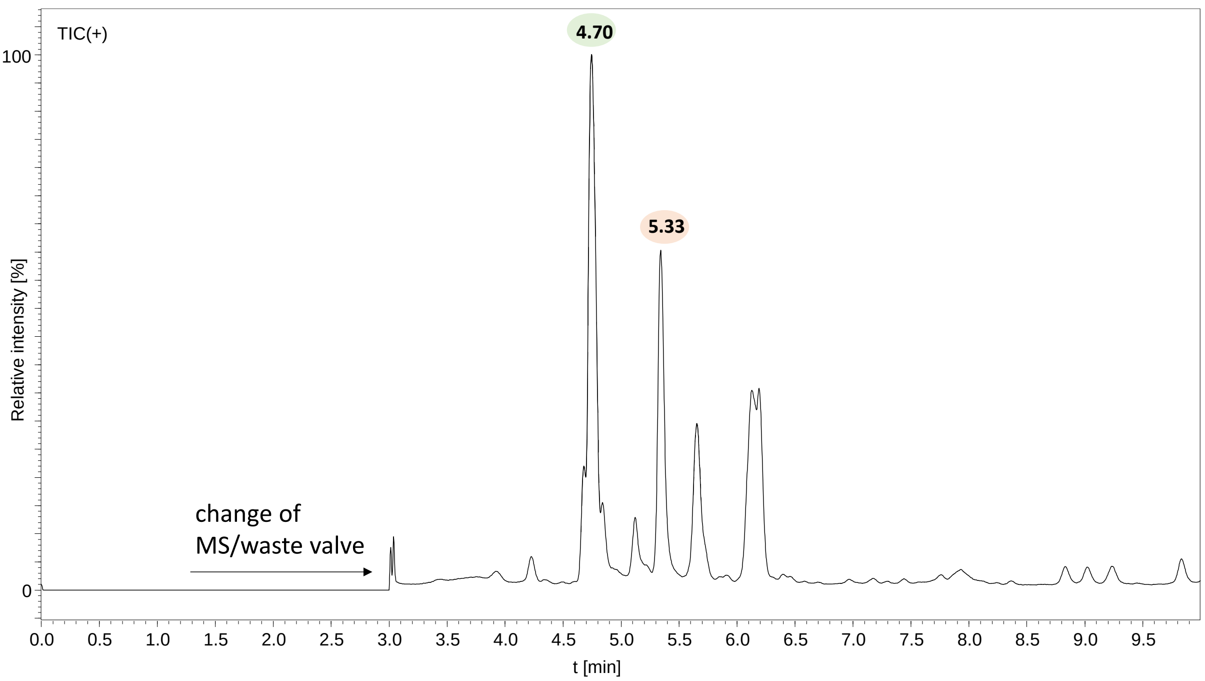

Fig S 26. LC-MS chromatogram (TIC) obtained after desulfurization of H-Thr-Gly-Cys-Ala-Phe-Lys-NH_2 (0.1 eq Rose Bengal, 1 mg TCEP, pH 5, 90 min)

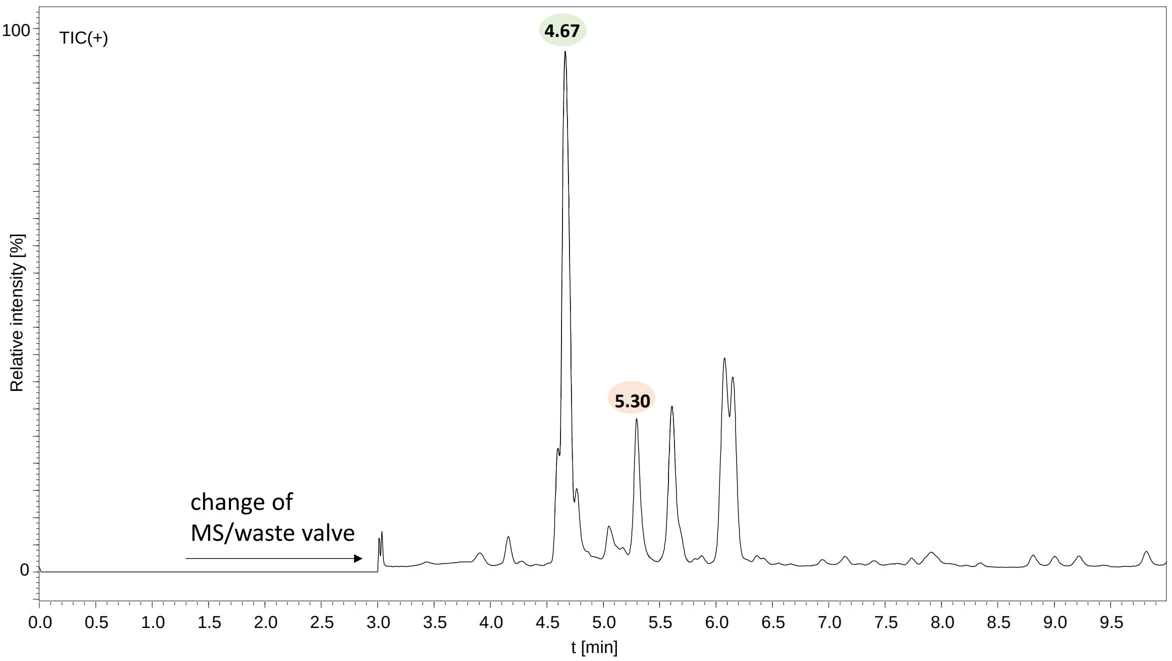

Fig S 27. LC-MS chromatogram (TIC) obtained after desulfurization of H-Thr-Gly-Cys-Ala-Phe-Lys-NH_2 (0.1 eq Rose Bengal, 1 mg TCEP, pH 5, 160 min)

14.7 Desulfurization of model peptide H-Thr-Gly-Cys-Ala-Phe-Lys-NH_2

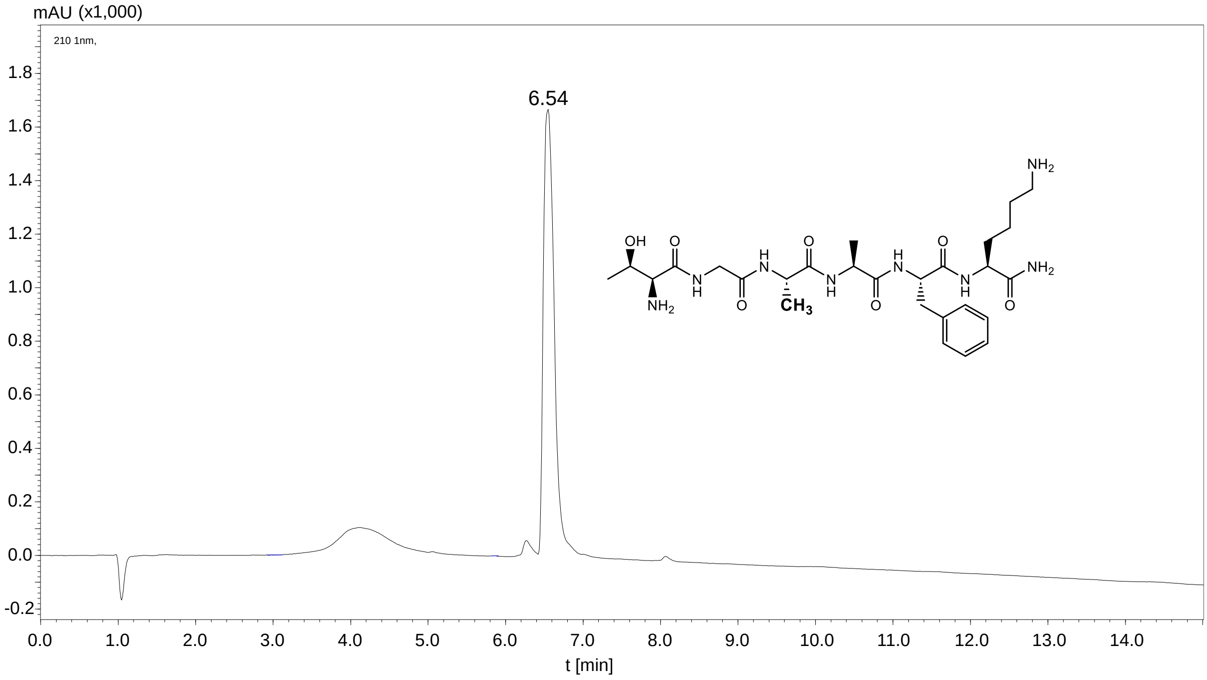

Fig S 28. HPLC chromatogram obtained for purified peptide H-Thr-Gly-Ala-Ala-Phe-Lys-NH_2 (desulfurization of peptide H-Thr-Gly-Cys-Ala-Phe-Lys-NH_2)

14.8 Desulfurization of peptide H-Ala-Phe-Cys-NH_2

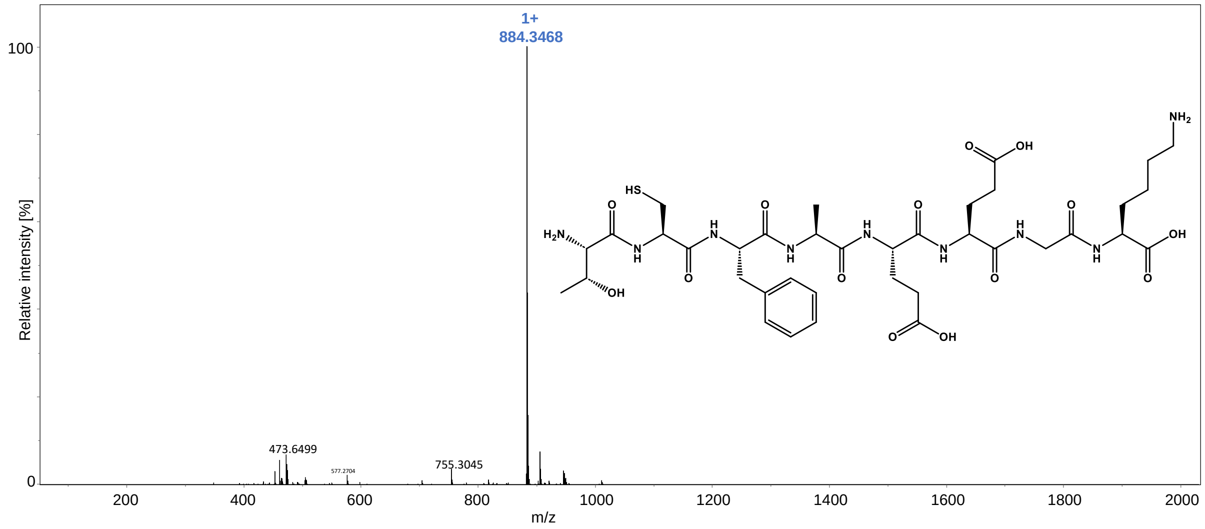

Fig S 29. ESI-MS spectrum obtained for purified peptide H-Ala-Phe-Cys-NH_2.

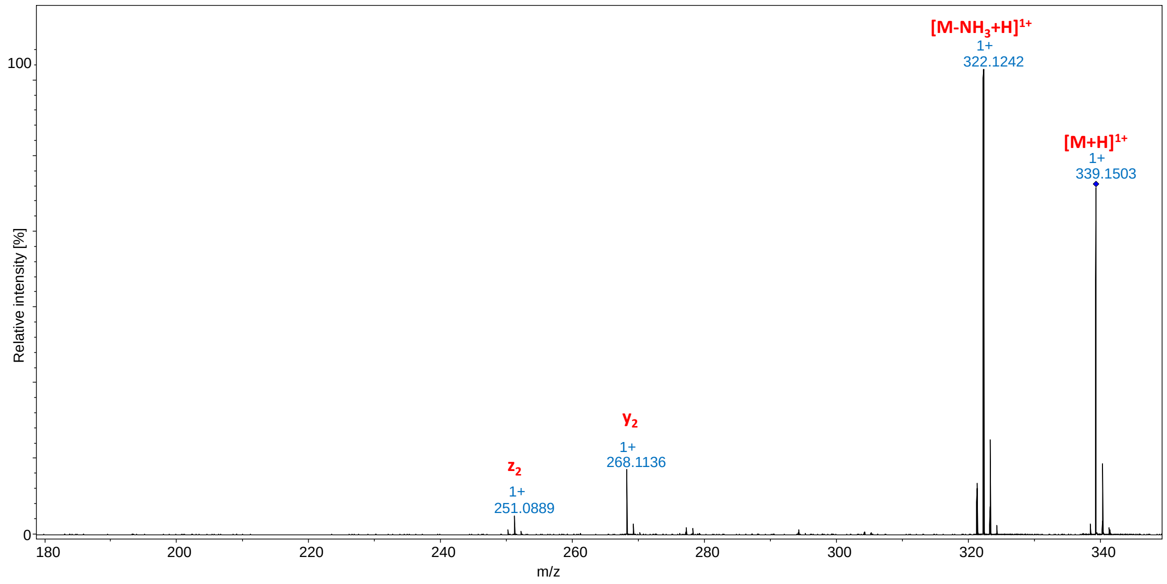

Fig S 30. ESI-MS/MS (10 eV) spectrum obtained for purified peptide H-Ala-Phe-Cys-NH$_2$.

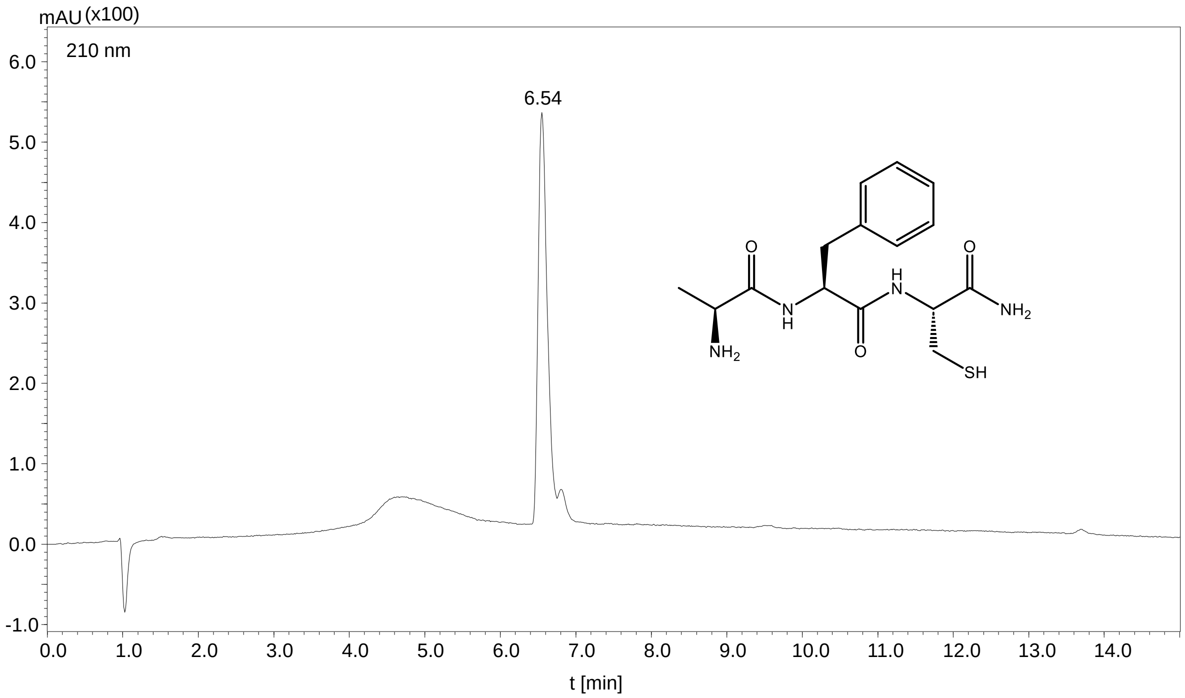

Fig S 31. HPLC chromatogram obtained for purified peptide H-Ala-Phe-Cys-NH$_2$.

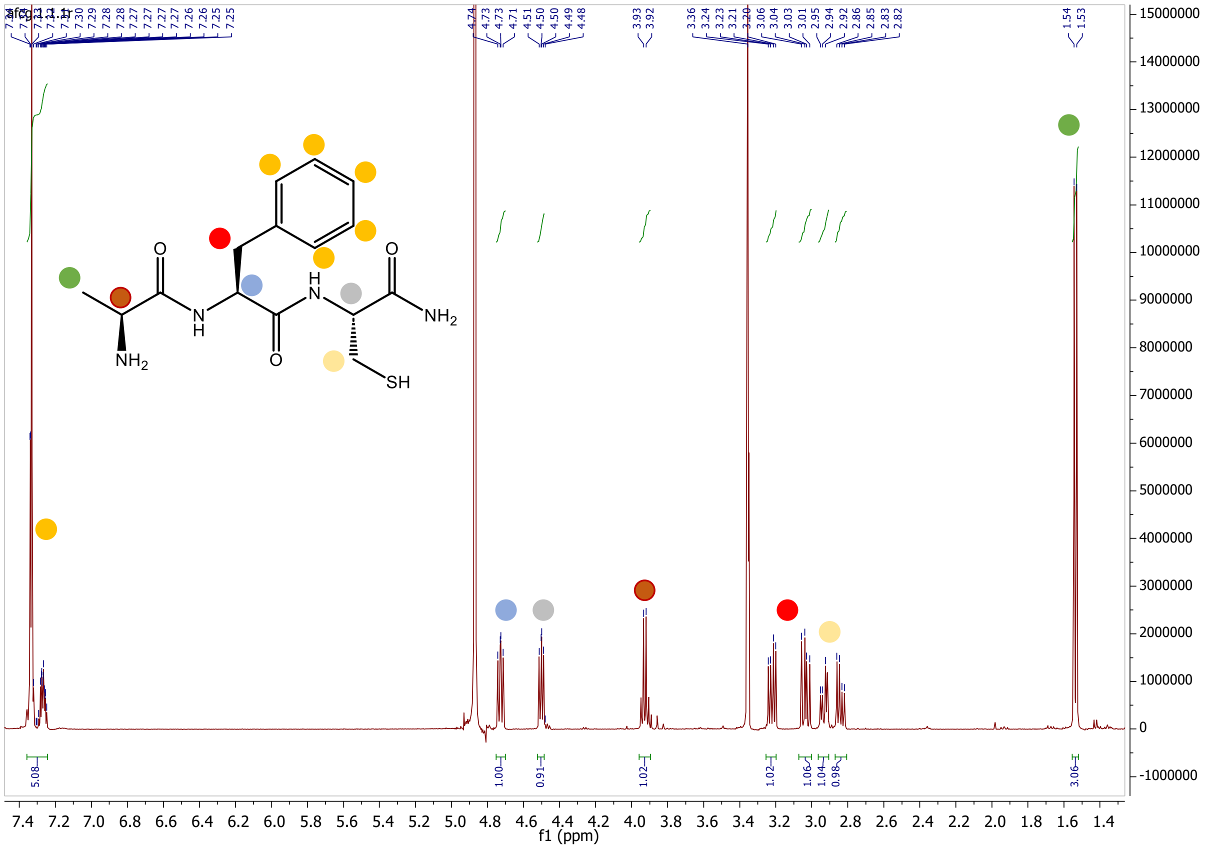

Fig S 32. ^{1}H NMR (MeOD, 500 MHz. 300K) spectrum obtained for peptide H-Ala-Phe-Cys-NH_2.

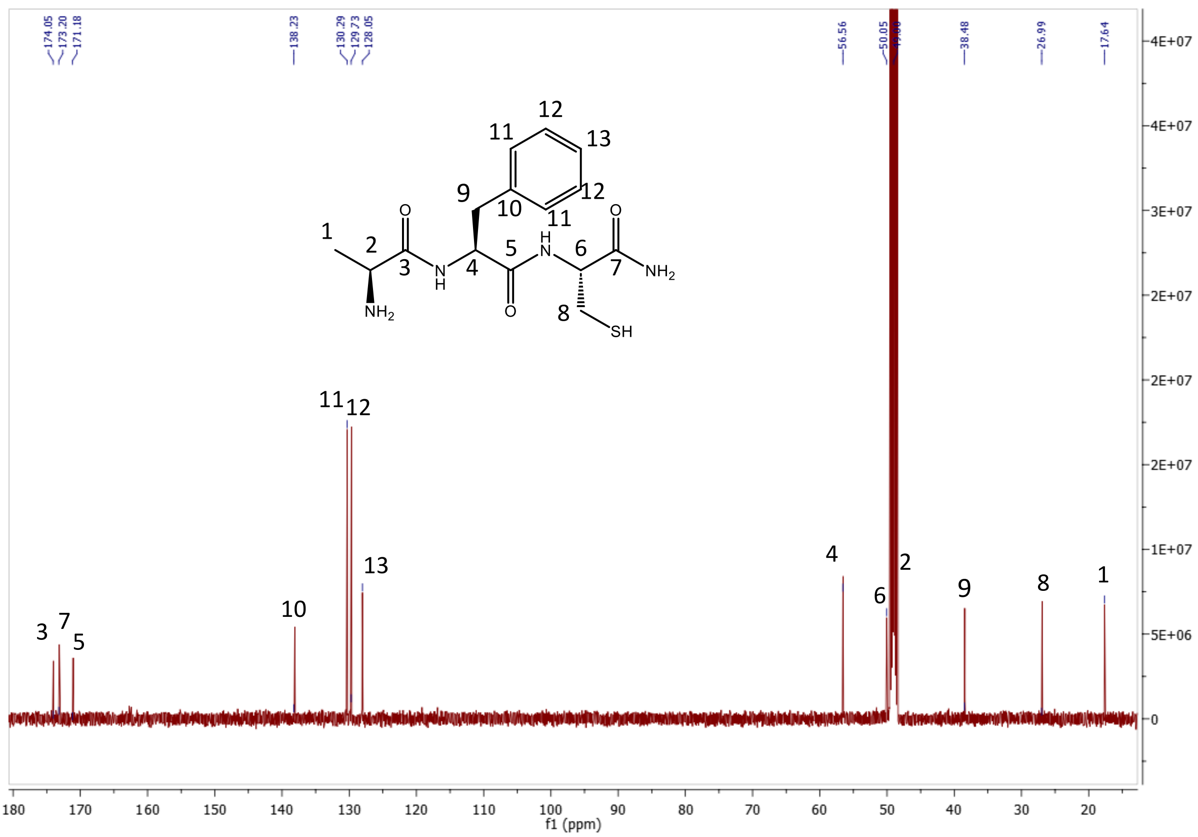

Fig S 33. ^{13}C NMR (126 MHz, methanol-d_4, 300K) spectrum obtained for peptide H-Ala-Phe-Cys-NH_2.

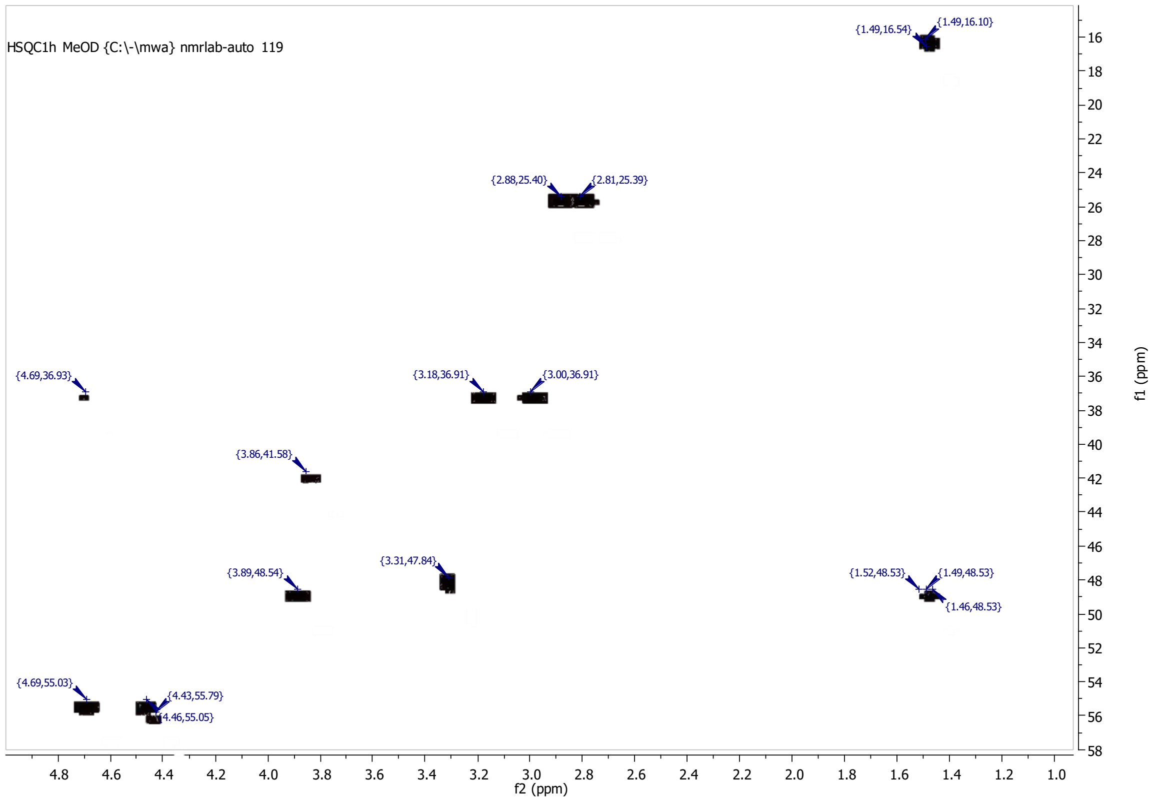

Fig S 34. 1H-13C HSQC NMR (500 MHz, methanol-d4 , 300 K) full spectrum of peptide H-Ala-Phe-Cys-NH_2 (expanded 4.9 – 1.0 ppm area).

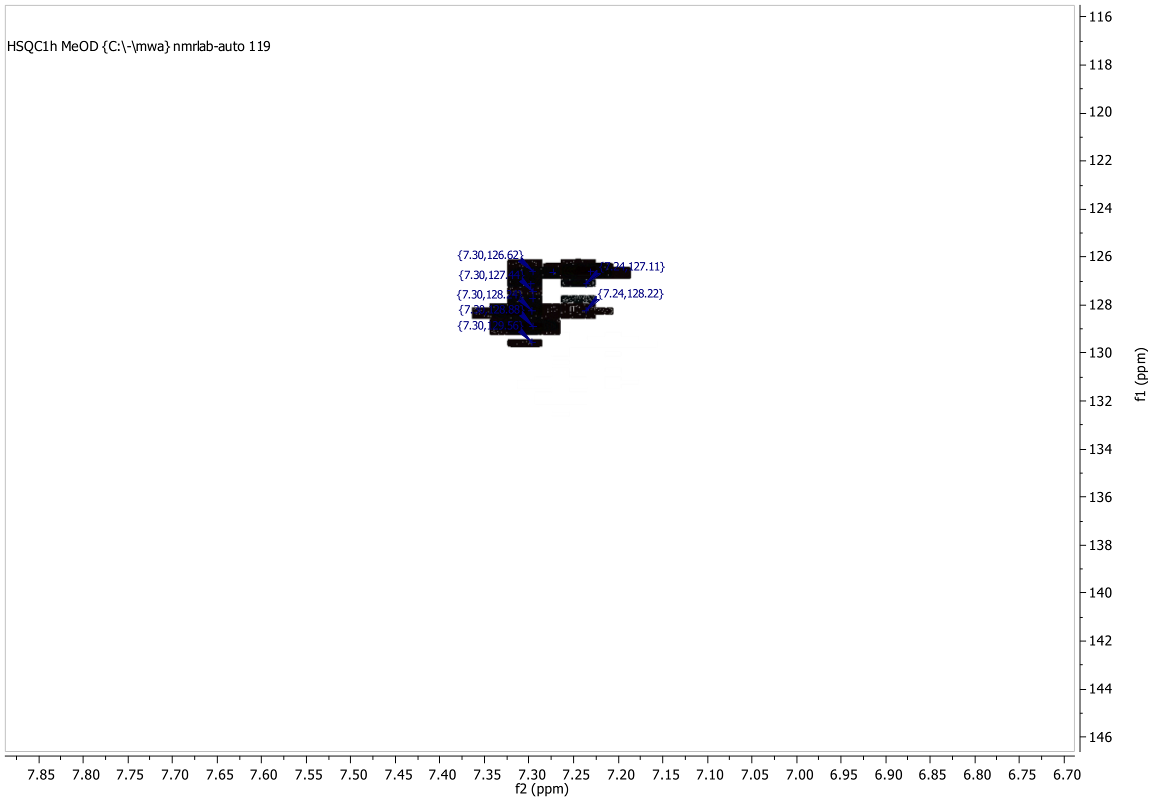

Fig S 35. 1H-13C HSQC NMR (500 MHz, methanol-d4 , 300 K) full spectrum of peptide H-Ala-Phe-Cys-NH_2 (expanded aromatic area).

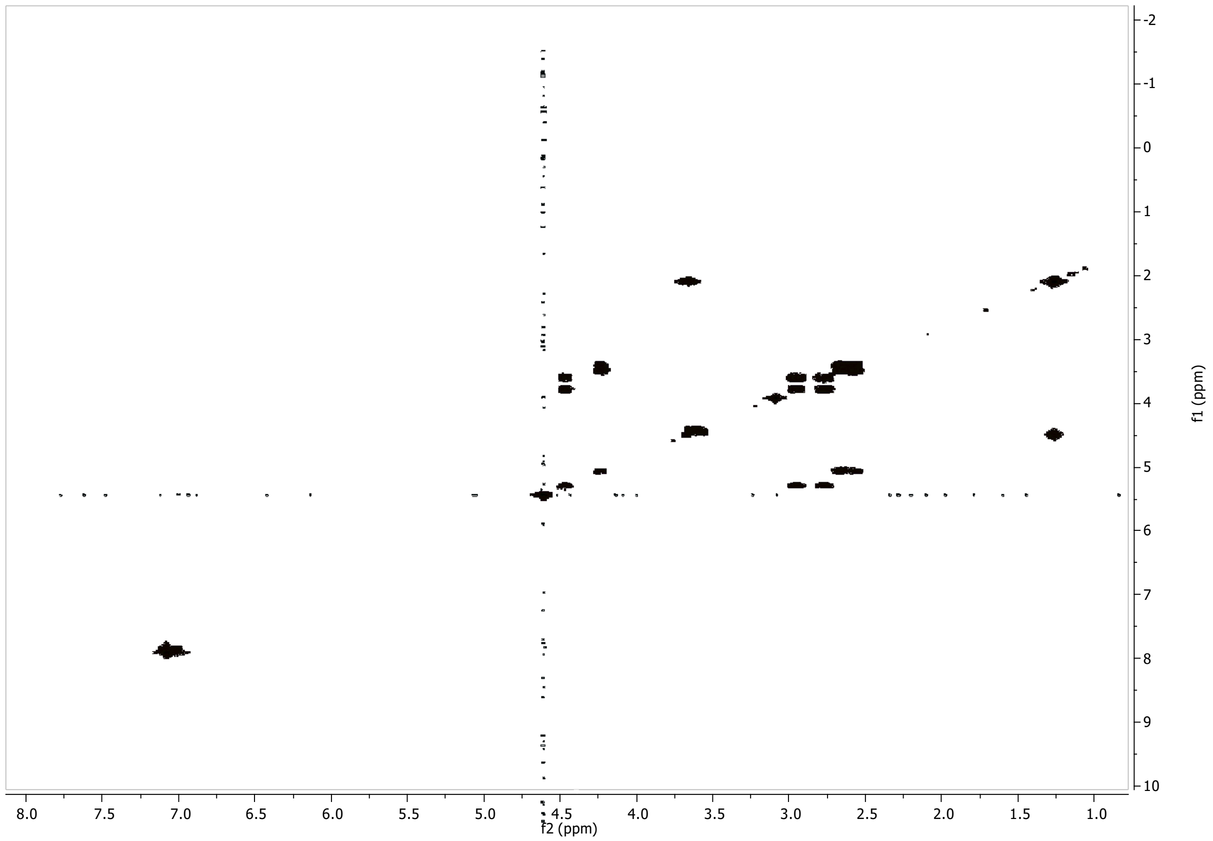

Fig S 36. 1H-1H COSY NMR (500 MHz, methanol-d4 , 300 K) full spectrum of peptide H-Ala-Phe-Cys-NH2)

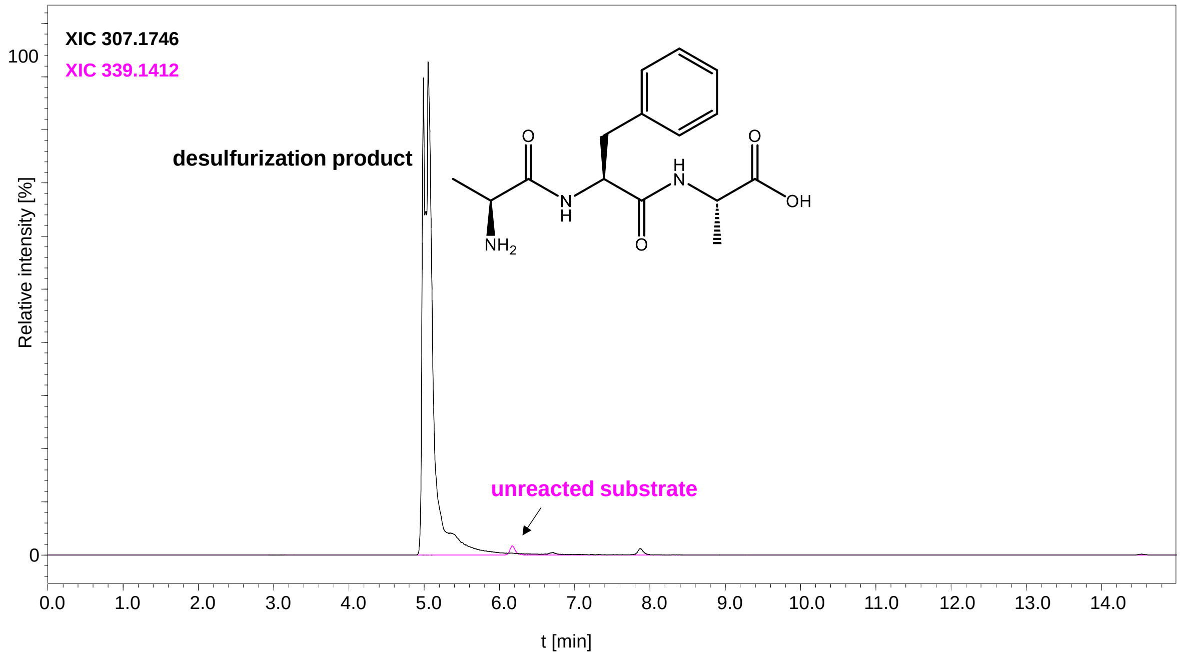

Fig S 37. LC-MS chromatogram (XIC) obtained for the mixture after desulfurization of peptide H-Ala-Phe-Cys-NH2.

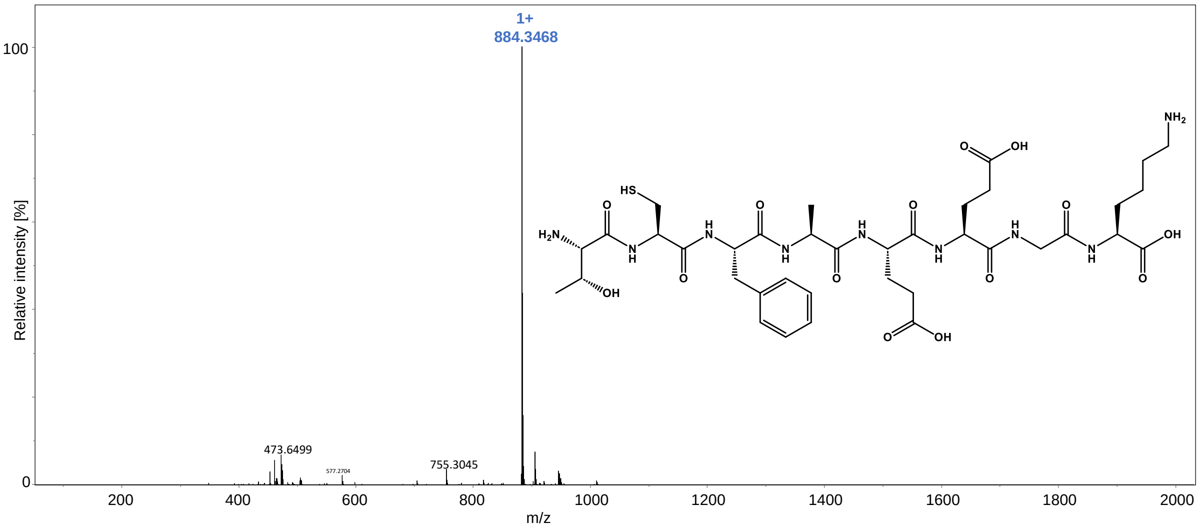

Fig S 38. ESI-MS spectrum obtained for purified peptide H-Ala-Phe-Ala-NH_2 (after desulfurization of peptide H-Ala-Phe-Cys-NH_2)

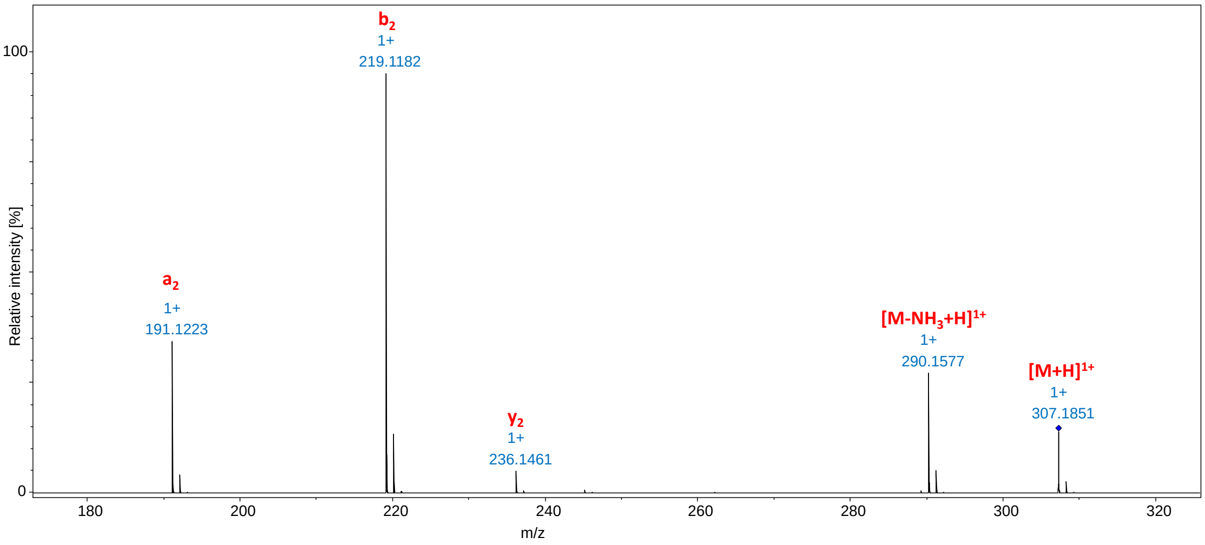

Fig S 39. ESI-MS/MS (10 eV) spectrum obtained for purified peptide H-Ala-Phe-Ala-NH_2 (after desulfurization of peptide H-Ala-Phe-Cys-NH_2)

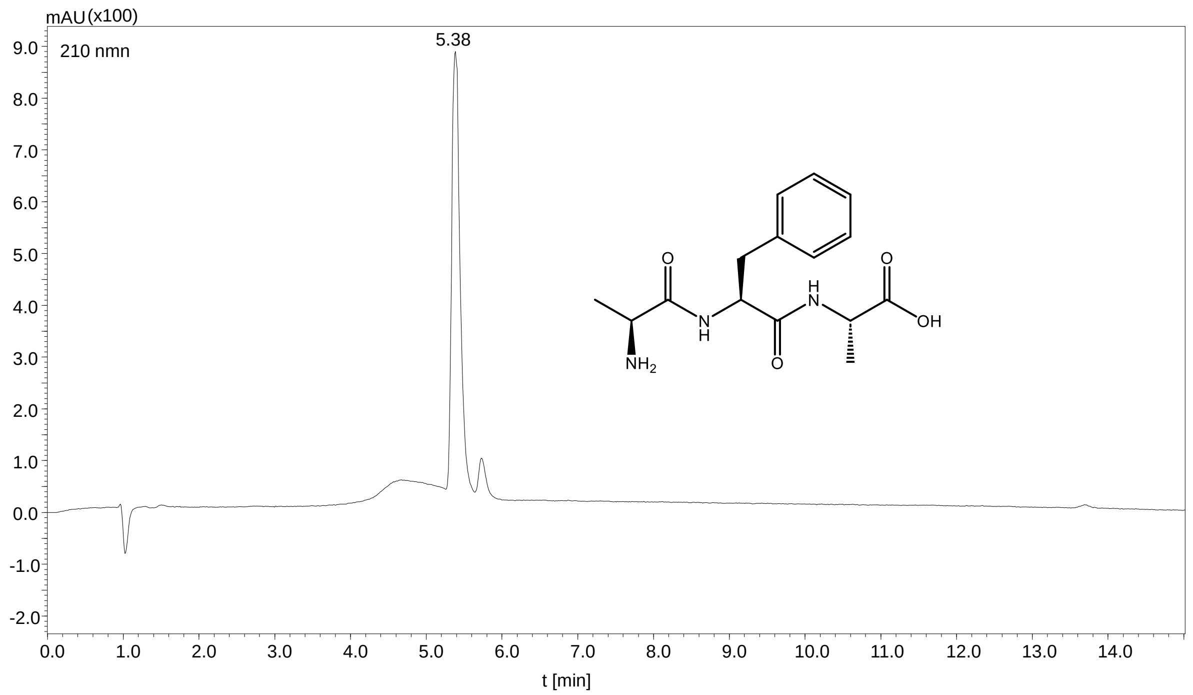

Fig S 40. HPLC chromatogram obtained for purified peptide H-Ala-Phe-Ala-NH_2 (after desulfurization of peptide H-Ala-Phe-Cys-NH_2)

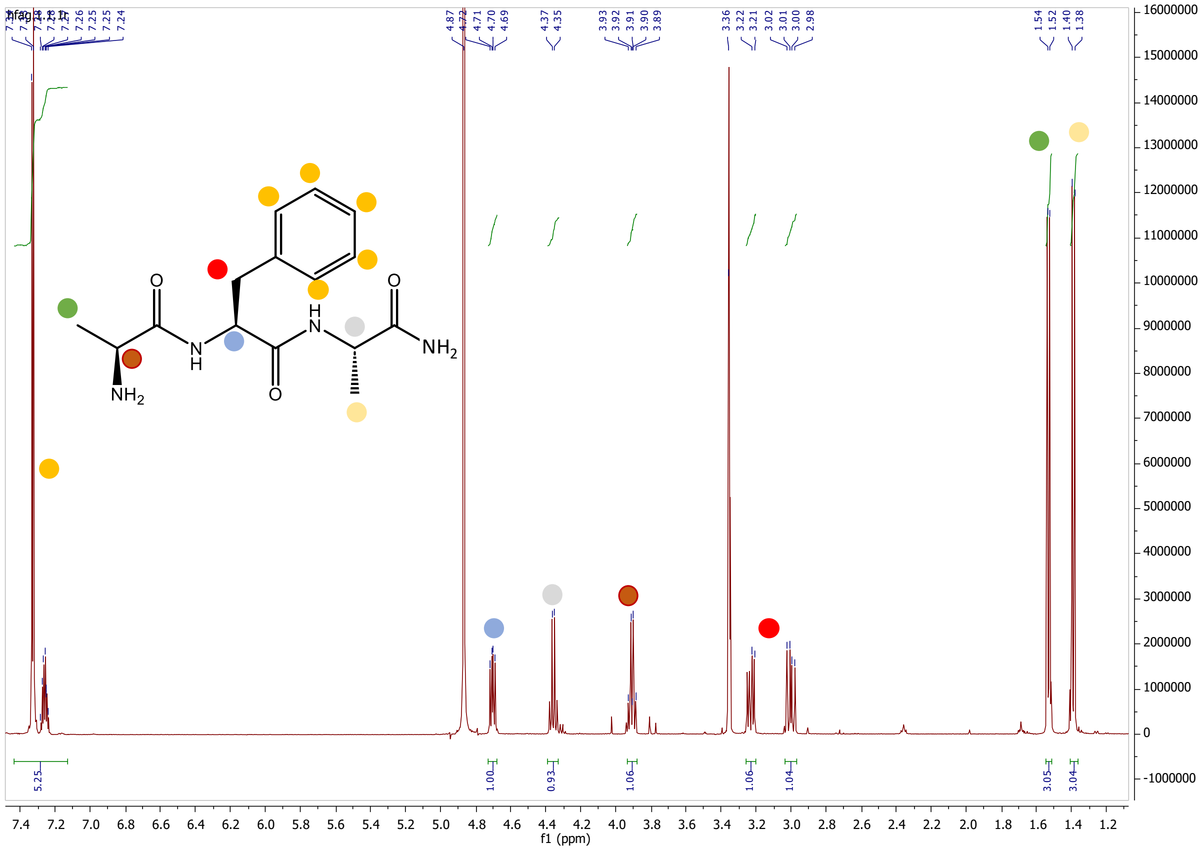

Fig S 41. ^1H NMR (MeOD, 500 MHz, 300K) spectrum obtained for peptide H-Ala-Phe-Ala-NH_2.

^{1}H NMR (500 MHz, MeOD) δ 7.35 – 7.24 (m, 5H), 4.70 (dd, J = 9.1, 5.9 Hz, 1H), 4.36 (q, J = 7.1 Hz, 1H), 3.93 – 3.88 (m, 1H), 3.23 (dd, J = 14.0, 5.9 Hz, 1H), 3.00 (dd, J = 14.0, 9.1 Hz, 1H), 1.53 (dd, J = 6.7, 3.5 Hz, 3H), 1.39 (d, J = 7.1 Hz, 3H).

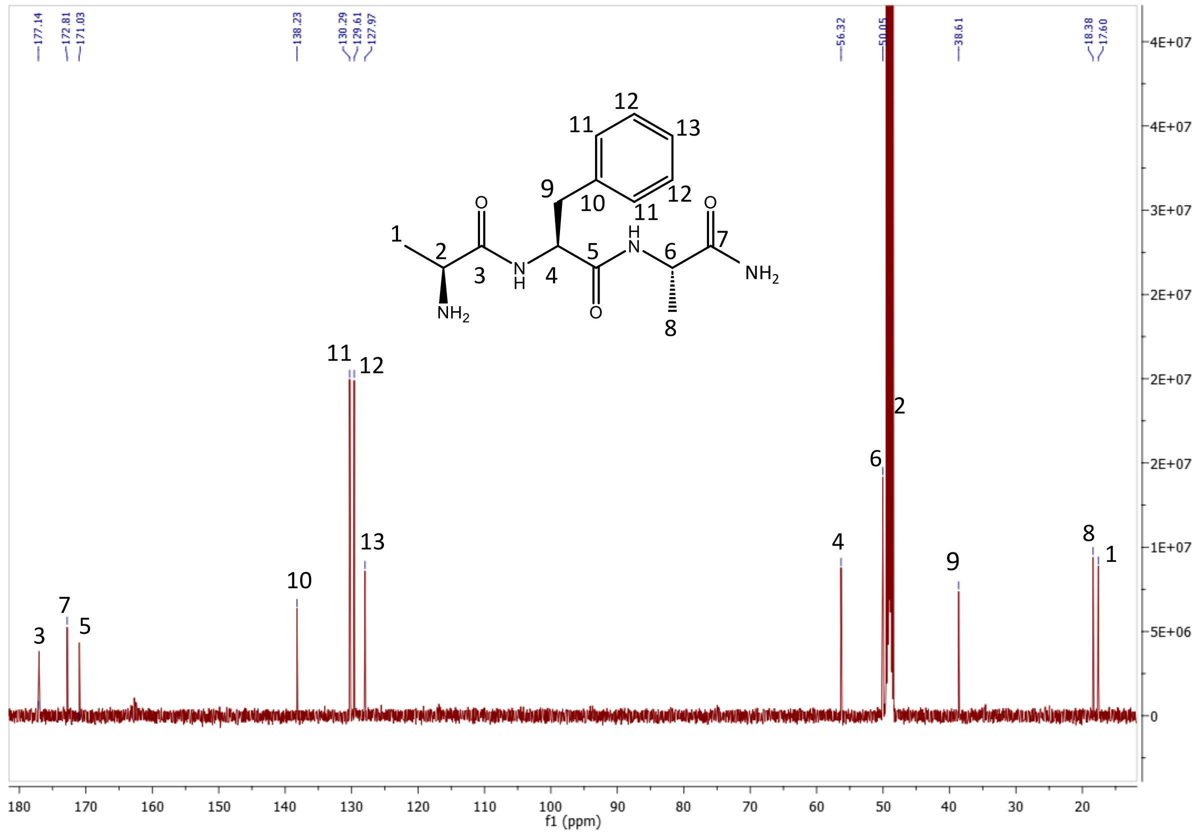

Fig S 42. ^{13}C NMR (126 MHz, methanol-d_4, 300K) spectrum obtained for peptide H-Ala-Phe-Ala-NH_2.

^{13}C NMR (126 MHz, methanol-d_4) δ 177.1, 172.8, 171.0, 138.2, 130.3, 129.6, 128.0, 56.3, 50.1, 38.6, 18.4, 17.6.

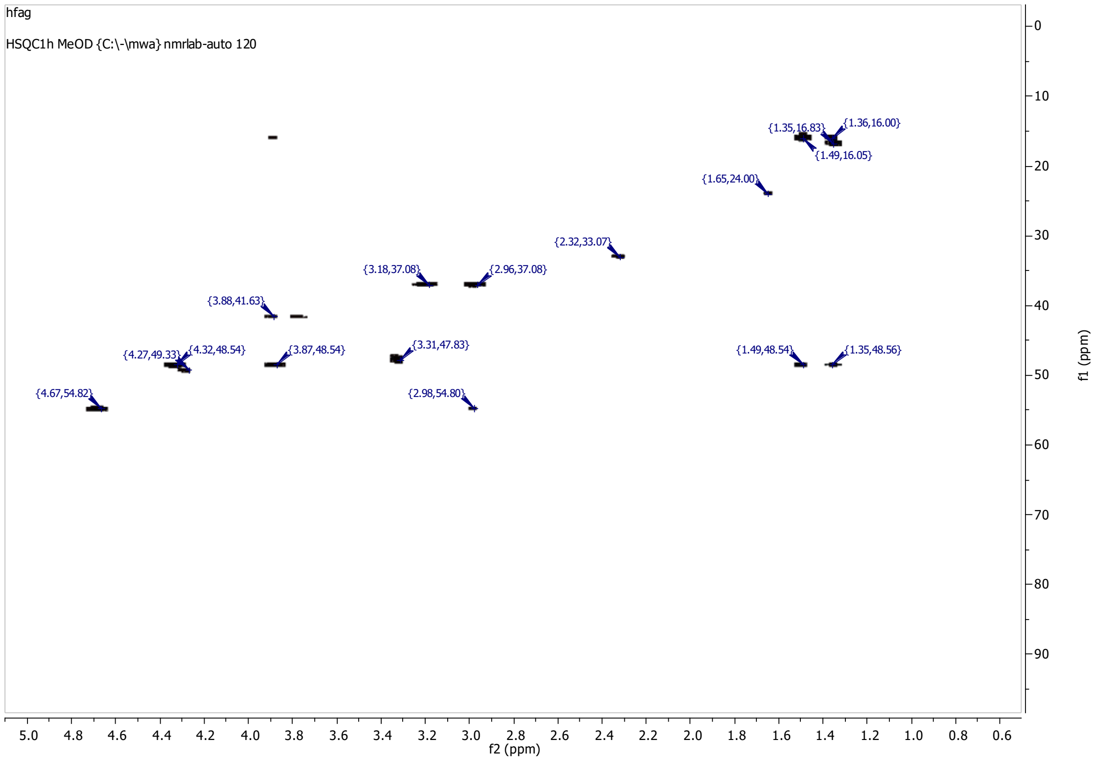

Fig S 43. 1H-13C HSQC NMR (500 MHz, methanol-d4 , 300 K) full spectrum of peptide H-Ala-Phe-Ala-NH_2 (expanded 5 – 0.6 ppm area).

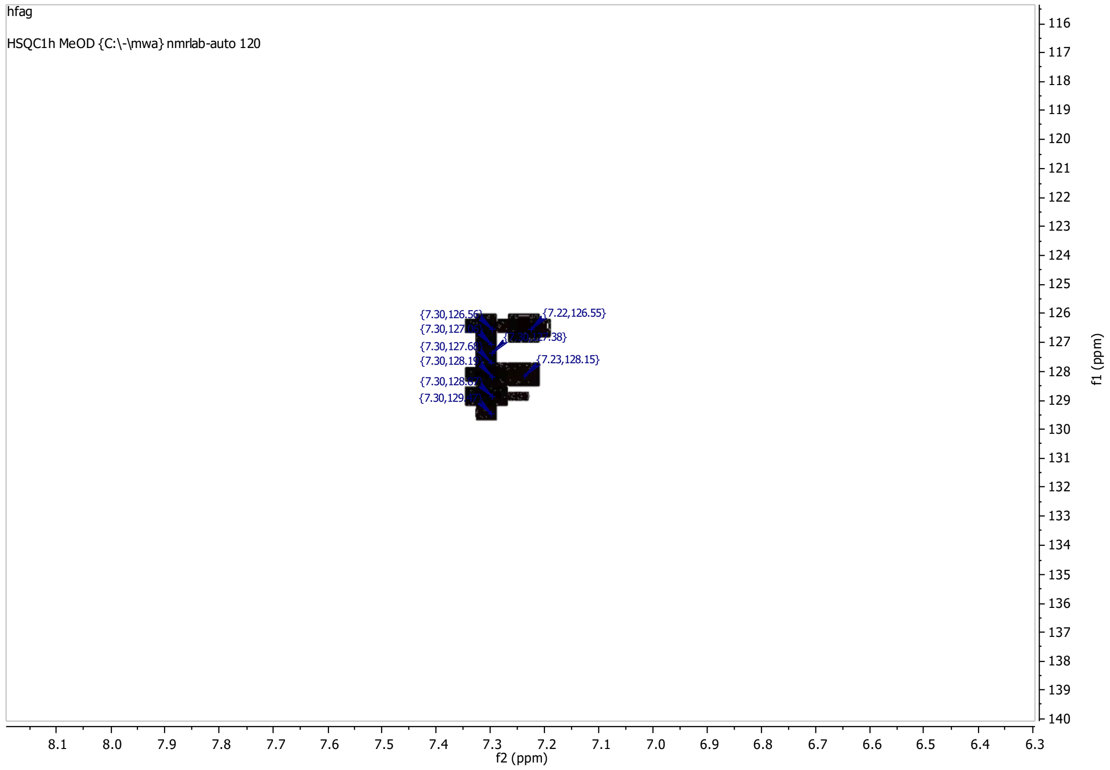

Fig S 44. 1H-13C HSQC NMR (500 MHz, methanol-d4, 300 K) full spectrum of peptide H-Ala-Phe-Ala-NH_2 (expanded aromatic area).

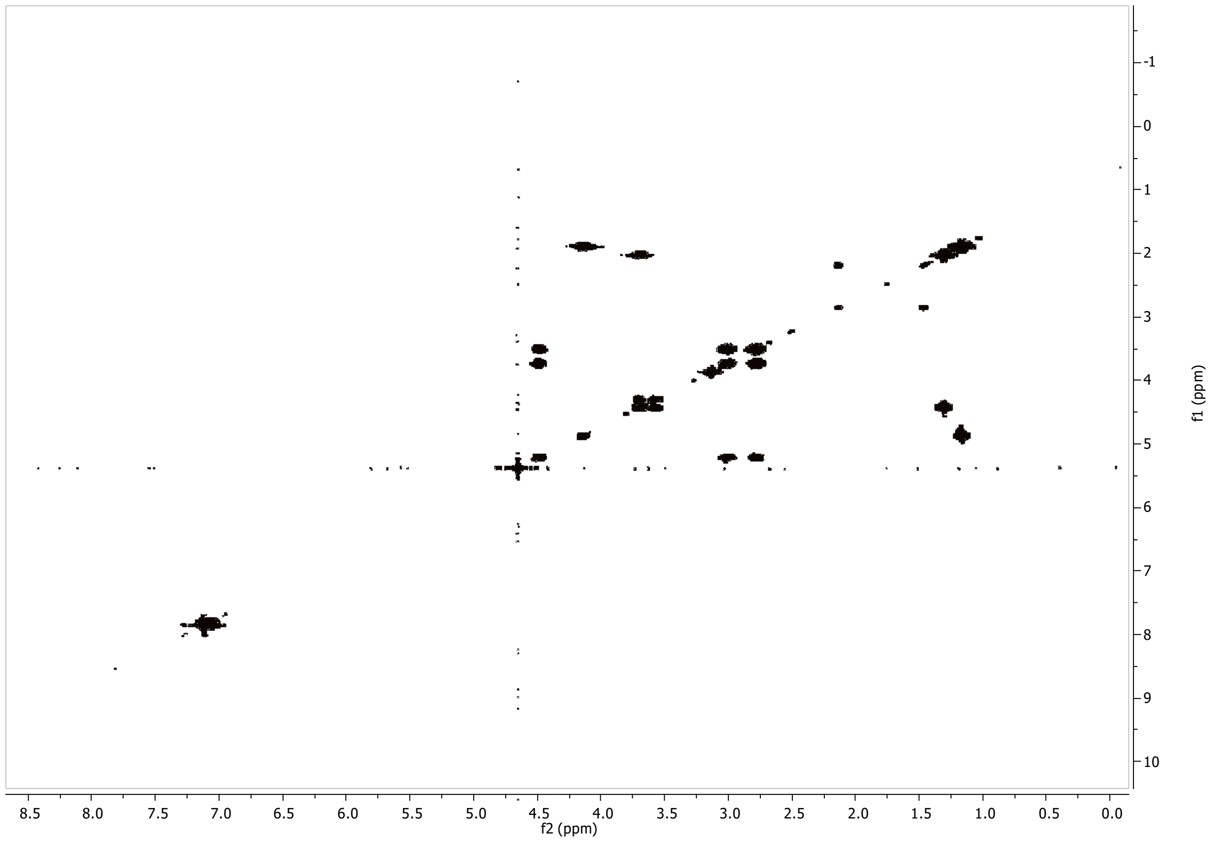

Fig S 45. 1H-1H COSY NMR (500 MHz, methanol-d4 , 300 K) full spectrum of peptide H-Ala-Phe-Ala-NH_2

14.9 Desulfurization of peptide H-Thr-Cys-Phe-Ala-Glu-Glu-Gly-Lys-OH

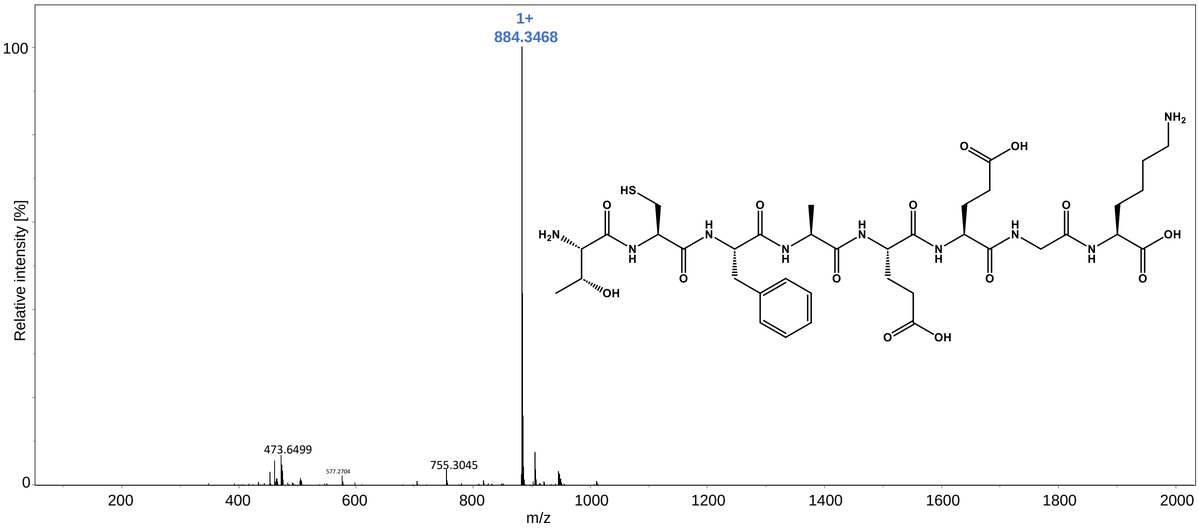

Fig S 46. ESI-MS spectrum obtained for purified peptide H-Thr-Ala-Phe-Ala-Glu-Glu-Gly-Lys-OH

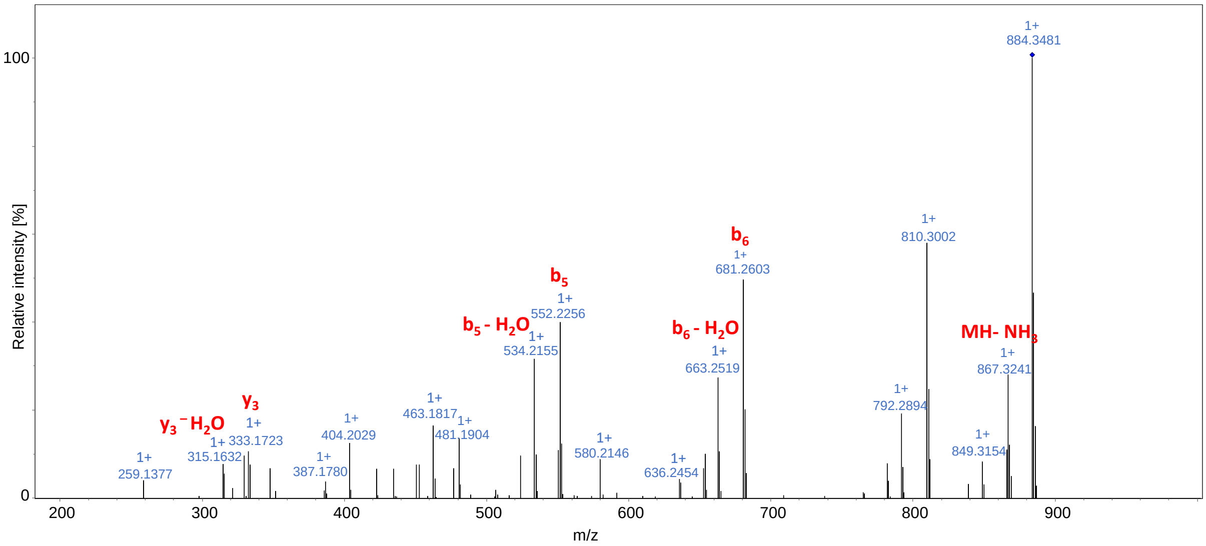

Fig S 47. ESI-MS/MS (CE 35eV) spectrum obtained for purified peptide H-Thr-Cys-Phe-Ala-Glu-Glu-Gly-Lys-OH

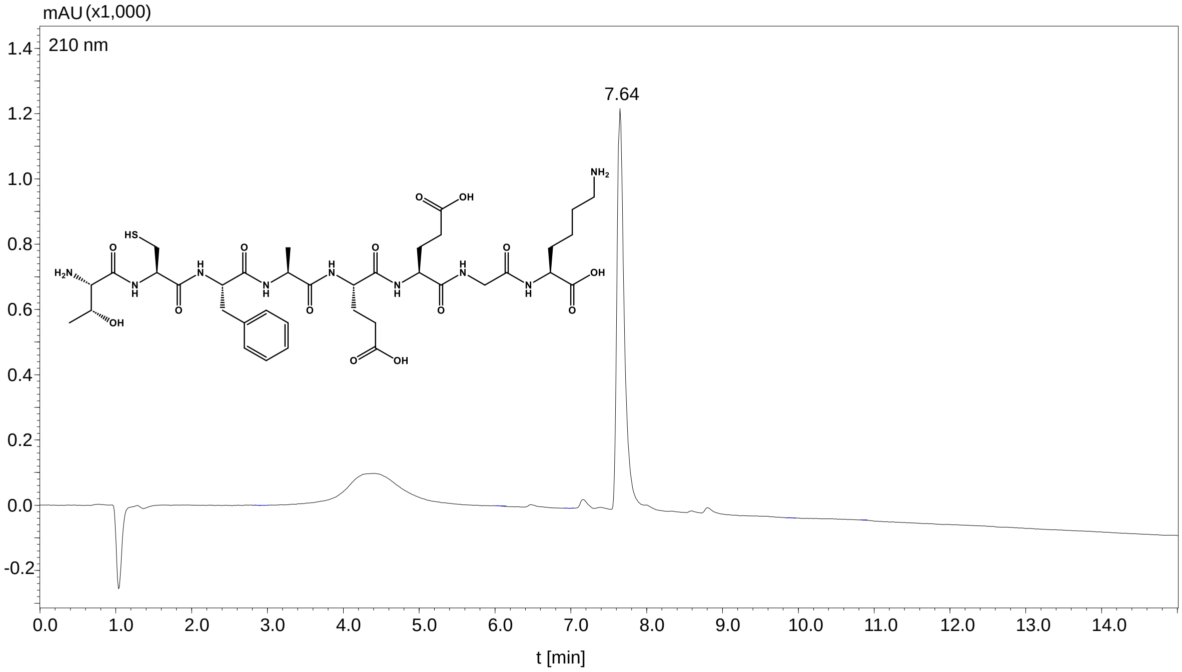

Fig S 48. HPLC chromatogram obtained for peptide H-Thr-Cys-Phe-Ala-Glu-Glu-Gly-Lys-OH.

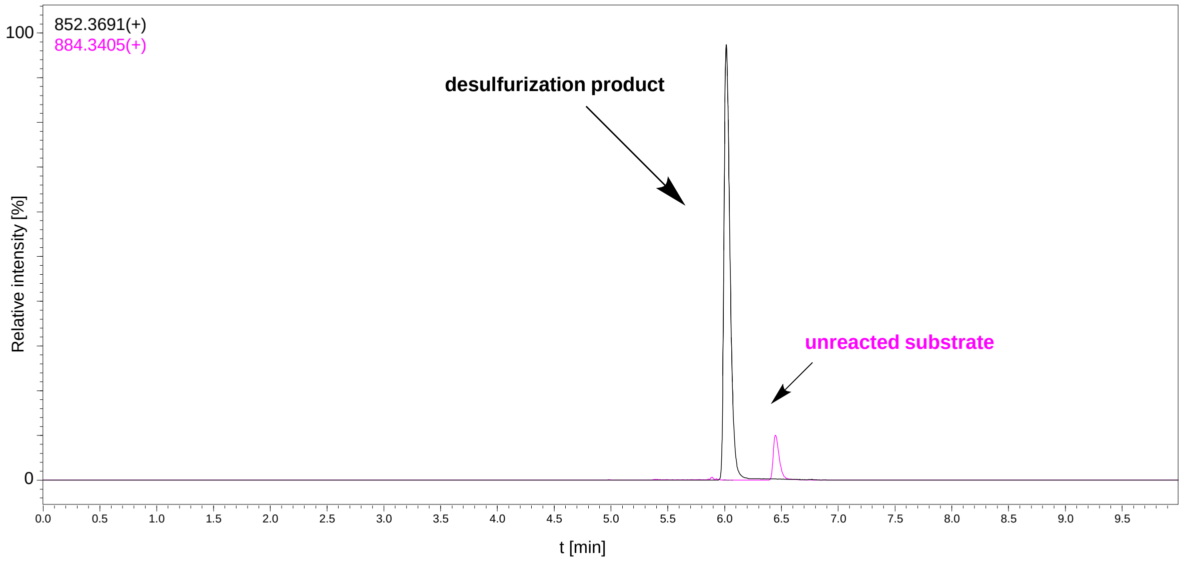

Fig S 49. LC-MS (XIC) spectrum obtained for the mixture after desulfurization of H-Thr-Cys-Phe-Ala-Glu-Glu-Gly-Lys-OH.

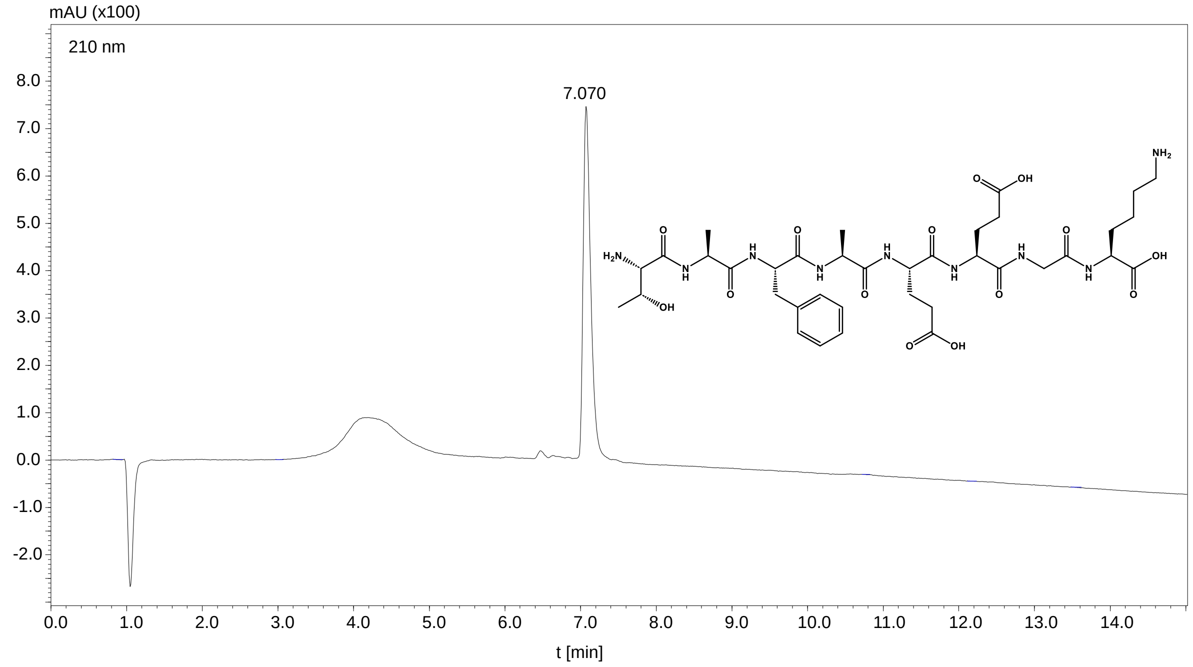

Fig S 50. HPLC chromatogram obtained for purified peptide H-Thr-Ala-Phe-Ala-Glu-Glu-Gly-Lys-OHafter desulfurization

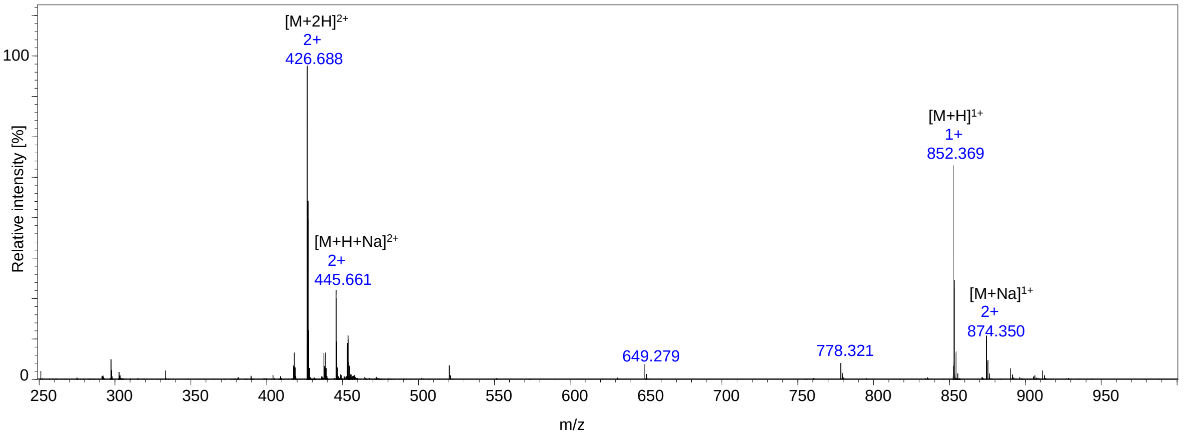

Fig S 51. ESI-MS spectrum obtained for purified peptide H-Thr-Ala-Phe-Ala-Glu-Glu-Gly-Lys-OH after desulfurization

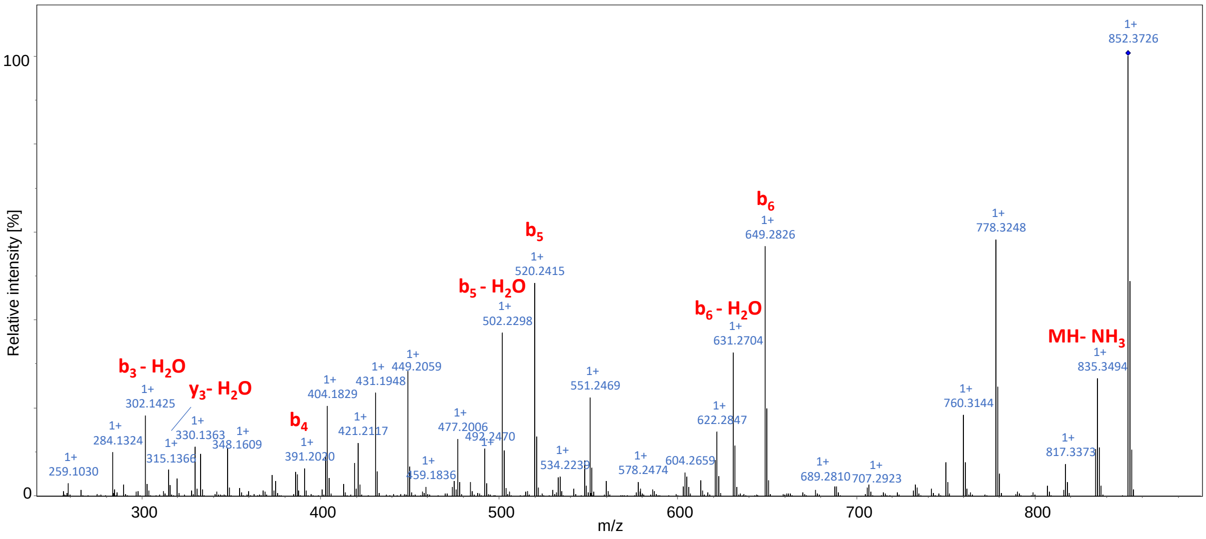

Fig S 52. ESI-MS/MS (CE 35eV) spectrum obtained for purified peptide H-Thr-Ala-Phe-Ala-Glu-Glu-Gly-Lys-OH after desulfurization

14.10 Desulfurization of peptide H-Cys-Glu-Leu-Phe-Glu-Gln-Leu-Gly-Glu-Tyr-Lys-OH

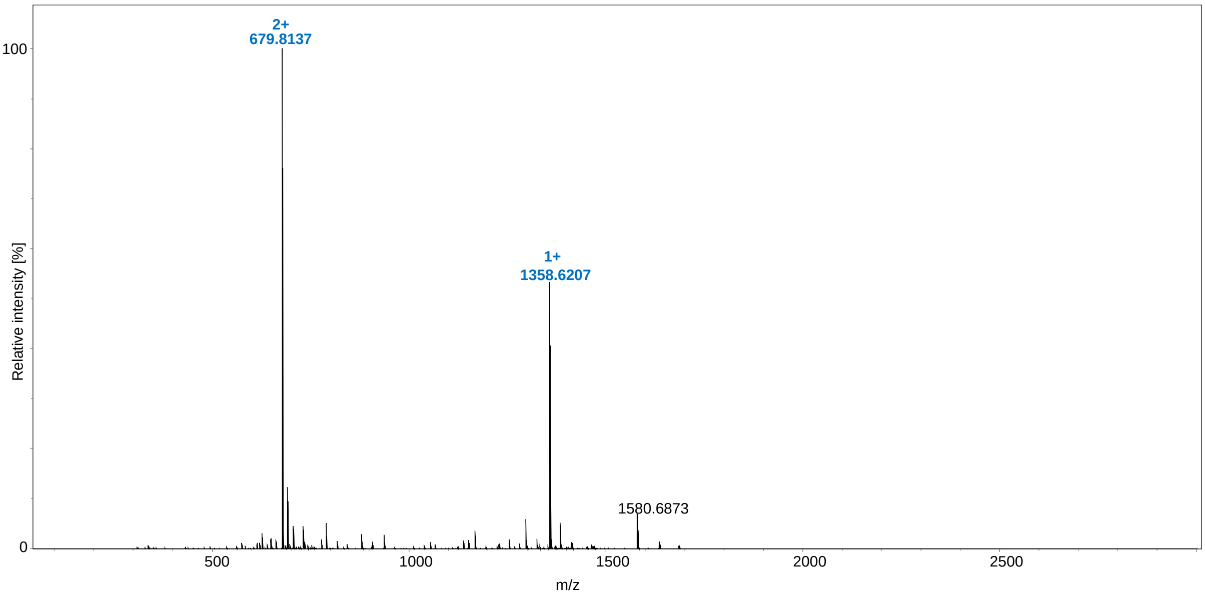

Fig S 53. ESI-MS spectrum obtained for purified peptide H-Cys-Glu-Leu-Phe-Glu-Gln-Leu-Gly-Glu-Tyr-Lys-OH

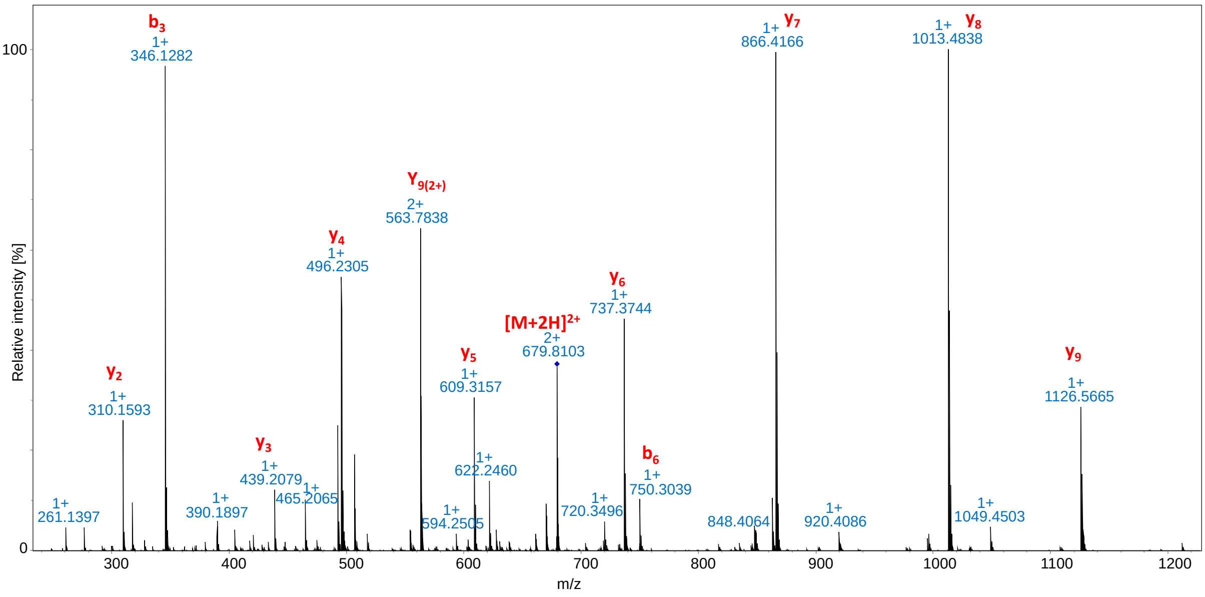

Fig S 54. ESI-MS/MS (25 eV) spectrum obtained for purified peptide H-Cys-Glu-Leu-Phe-Glu-Gln-Leu-Gly-Glu-Tyr-Lys-OH

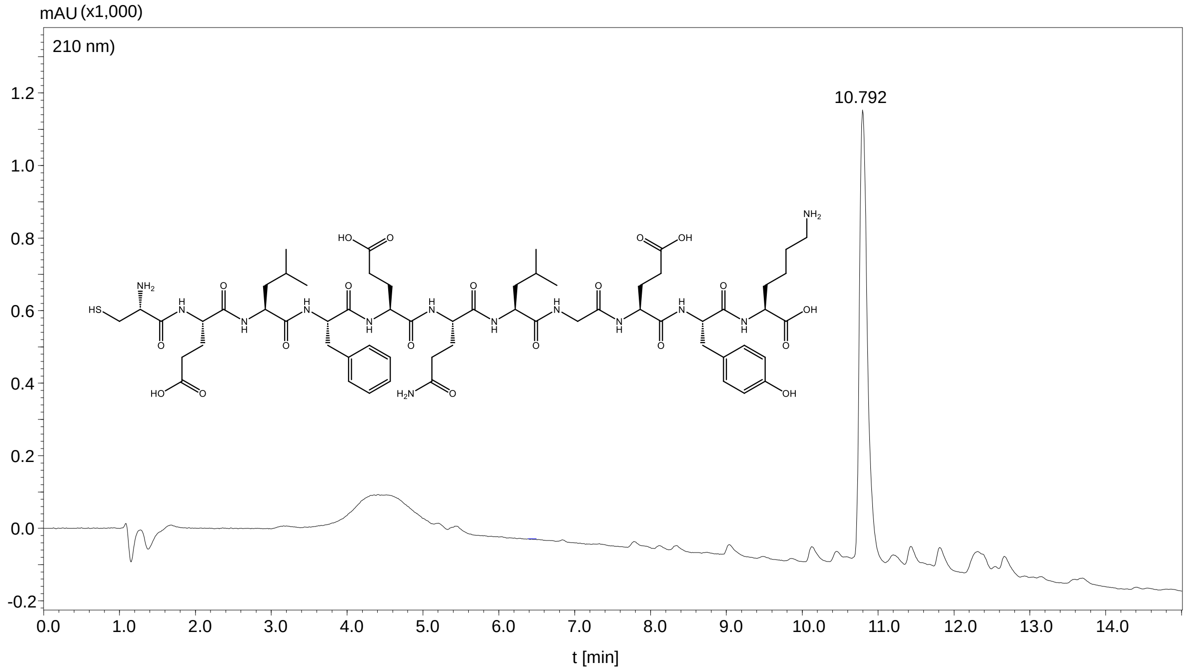

Fig S 55. HPLC chromatogram obtained for purified peptide H-Cys-Glu-Leu-Phe-Glu-Gln-Leu-Gly-Glu-Tyr-Lys-OH

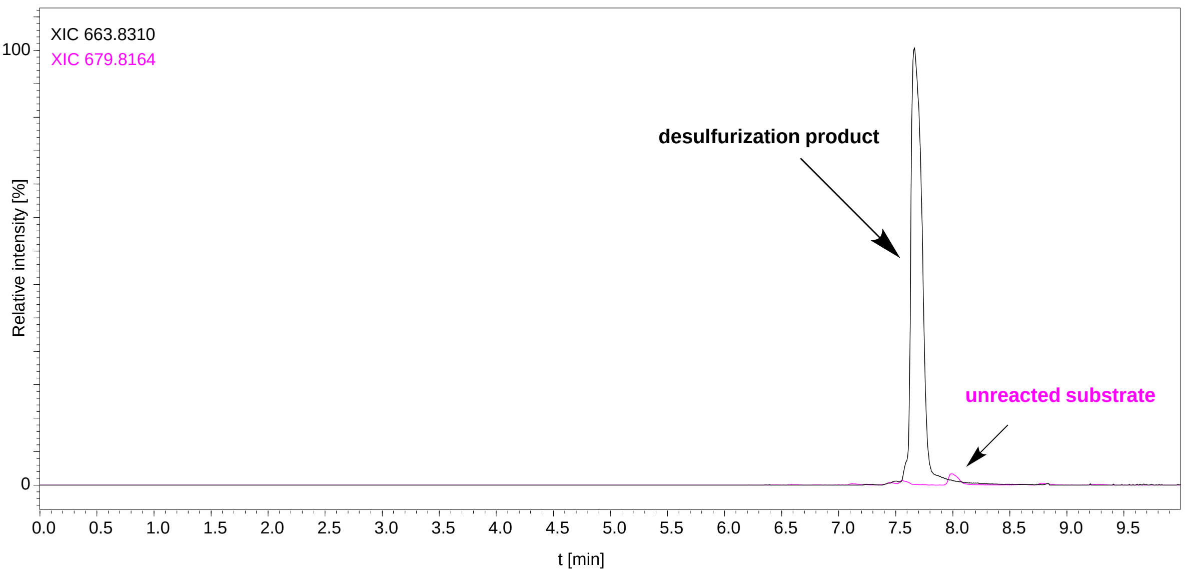

Fig S 56. LC-MS (XIC) chromatogram obtained for the mixture after desulfurization of peptide H-Cys-Glu-Leu-Phe-Glu-Gln-Leu-Gly-Glu-Tyr-Lys-OH

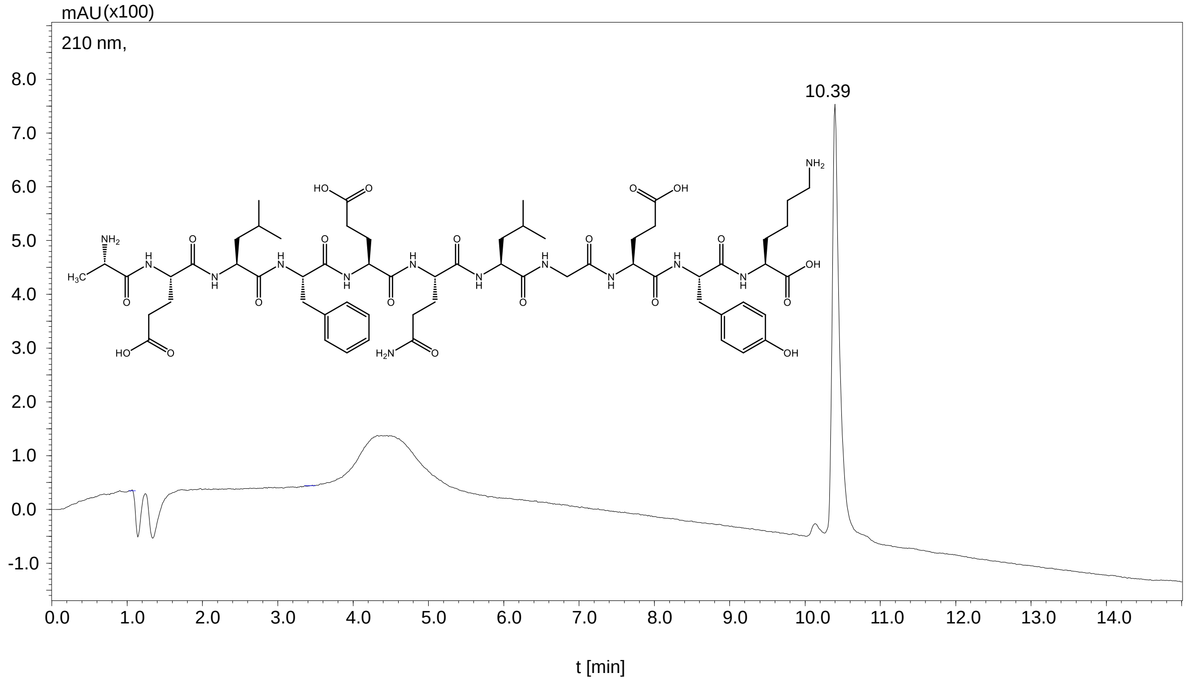

Fig S 57. HPLC chromatogram obtained for purified peptide H-Ala-Glu-Leu-Phe-Glu-Gln-Leu-Gly-Glu-Tyr-Lys-OH after desulfurization

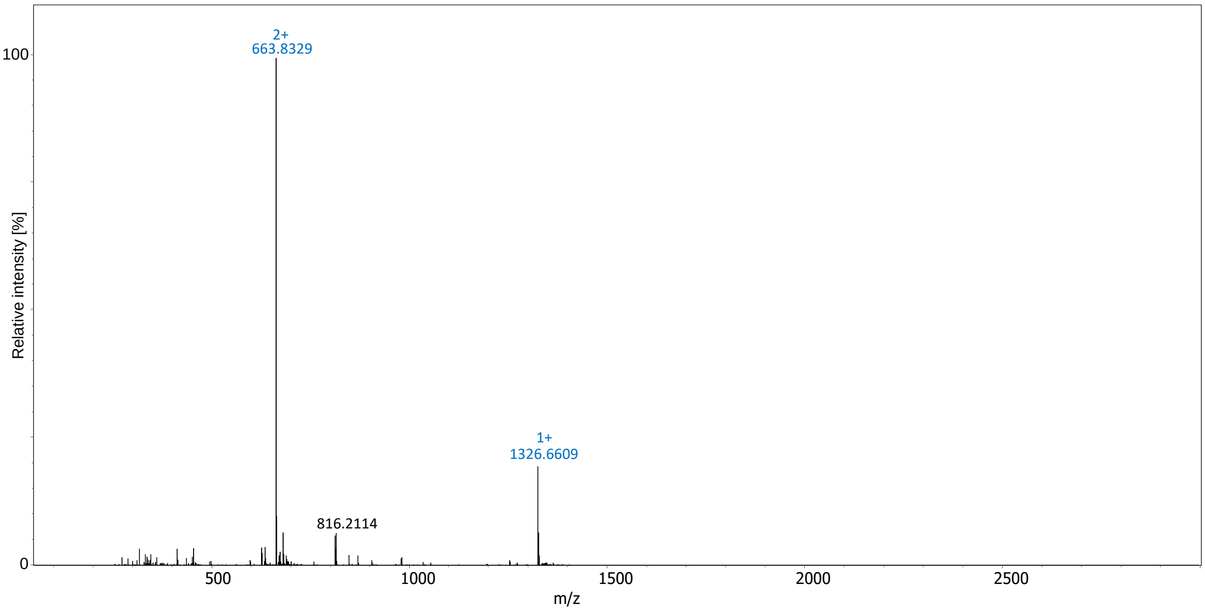

Fig S 58. ESI-MS spectrum obtained for purified peptide H-Ala-Glu-Leu-Phe-Glu-Gln-Leu-Gly-Glu-Tyr-Lys-OH after desulfurization

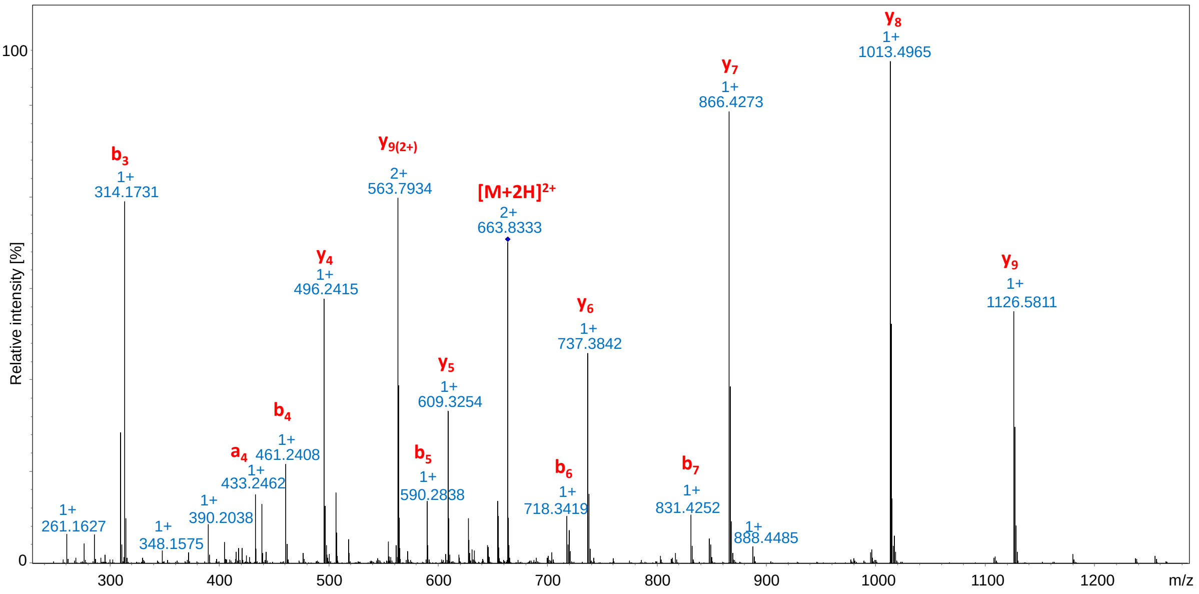

Fig S 59. ESI-MS/MS (CE 25eV) spectrum obtained for purified peptide H-Ala-Glu-Leu-Phe-Glu-Gln-Leu-Gly-Glu-Tyr-Lys-OH after desulfurization

14.11 Desulfurization of peptide H-Cys-Ile-Leu-Lys-Glu-Pro-Val-His-Gly-Val-NH_2

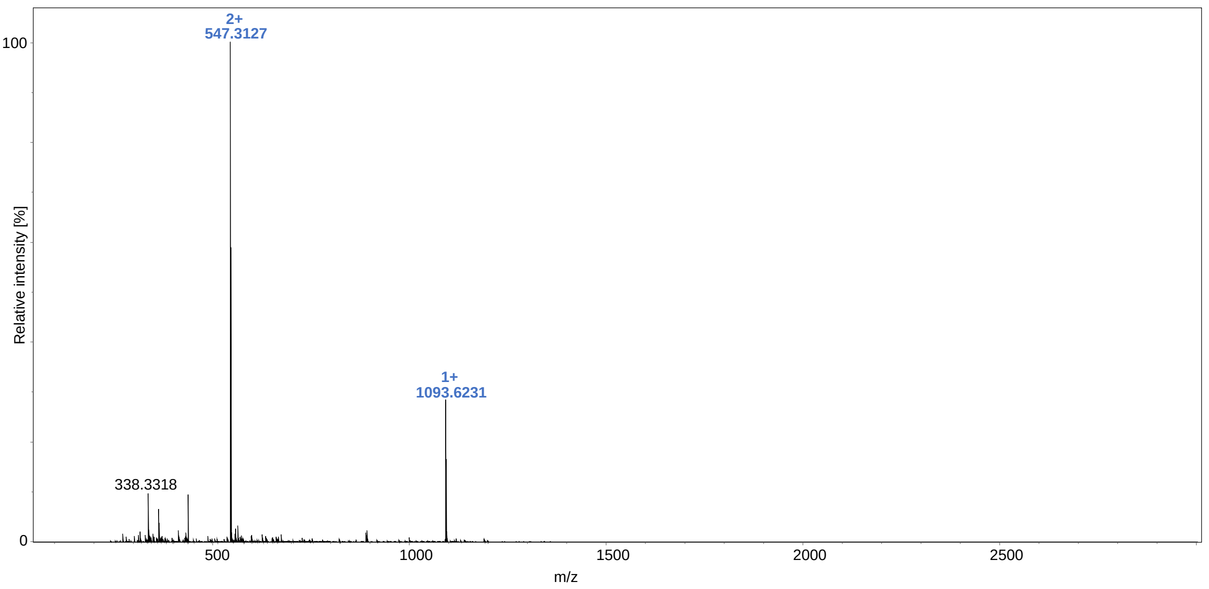

Fig S 60. ESI-MS spectrum obtained for purified peptide H-Cys-Ile-Leu-Lys-Glu-Pro-Val-His-Gly-Val-NH_2

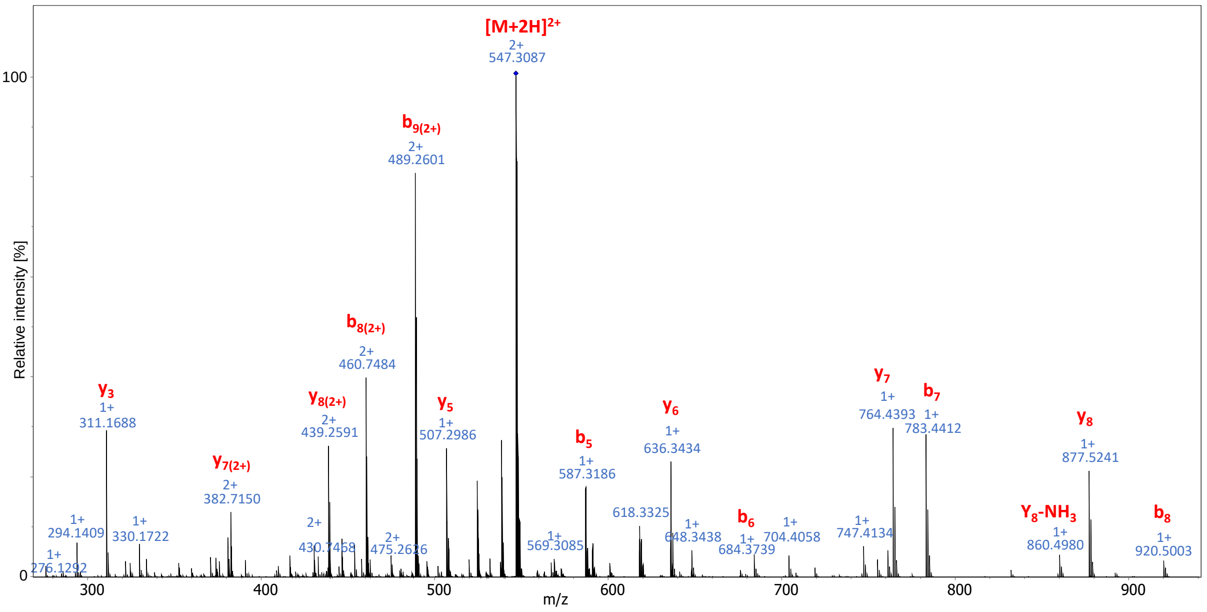

Fig S 61. ESI-MS/MS (CE 25eV) spectrum obtained for purified peptide H-Cys-Ile-Leu-Lys-Glu-Pro-Val-His-Gly-Val-NH_2

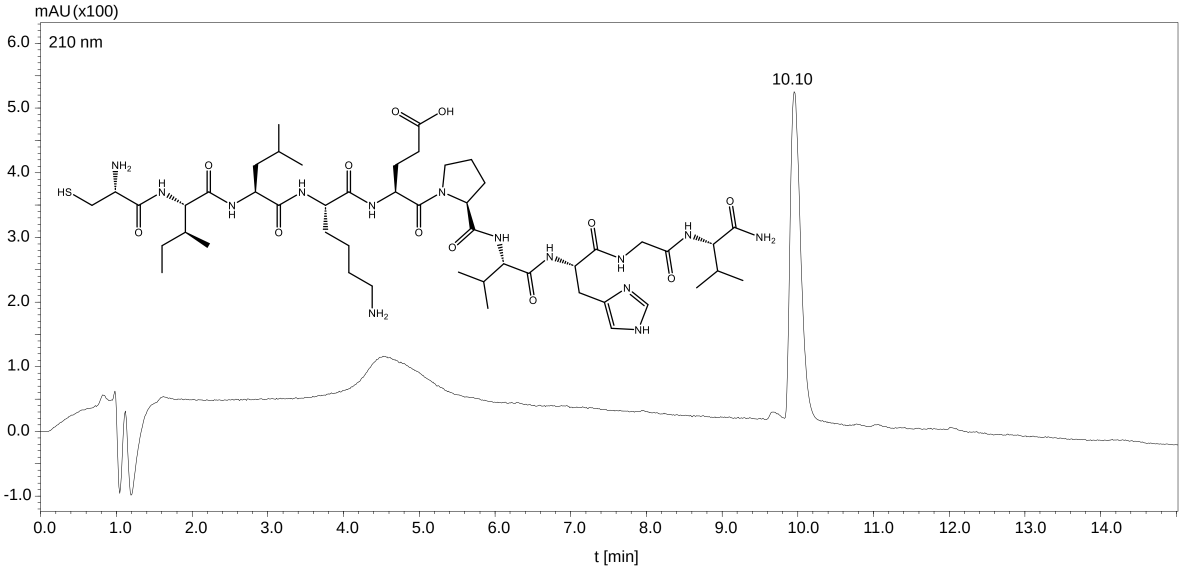

Fig S 62. ESI-MS spectrum obtained for purified peptide H-Cys-Ile-Leu-Lys-Glu-Pro-Val-His-Gly-Val-NH_2

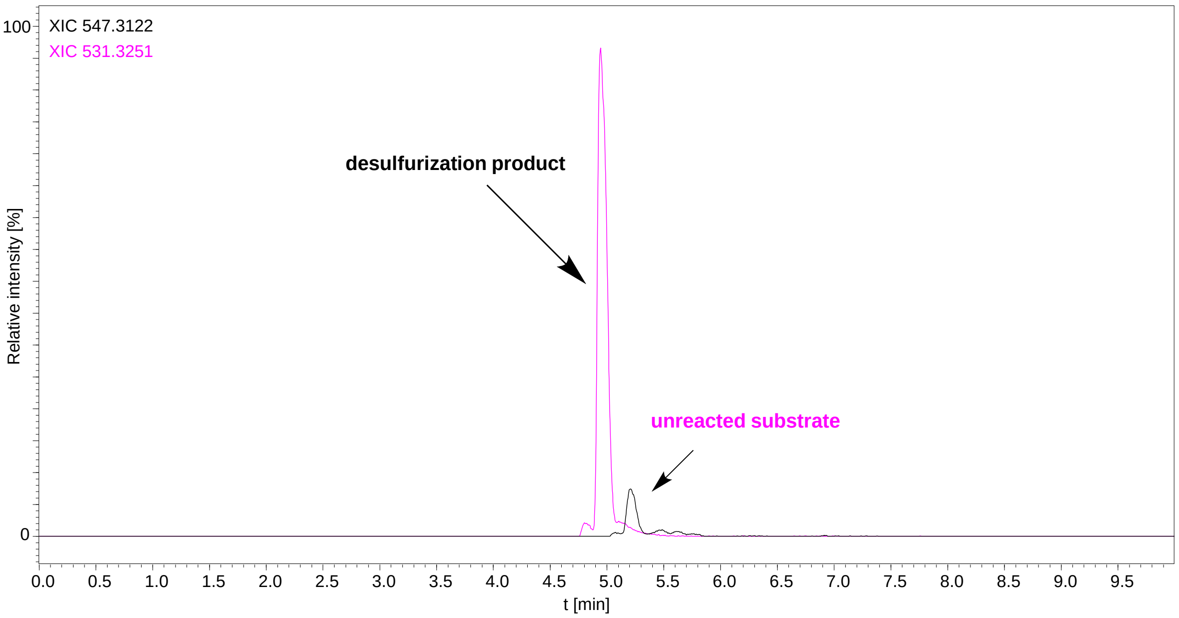

Fig S 63. LC-MS (XIC) chromatogram obtained for the mixture after desulfurization of peptide H-Cys-Ile-Leu-Lys-Glu-Pro-Val-His-Gly-Val-NH_2

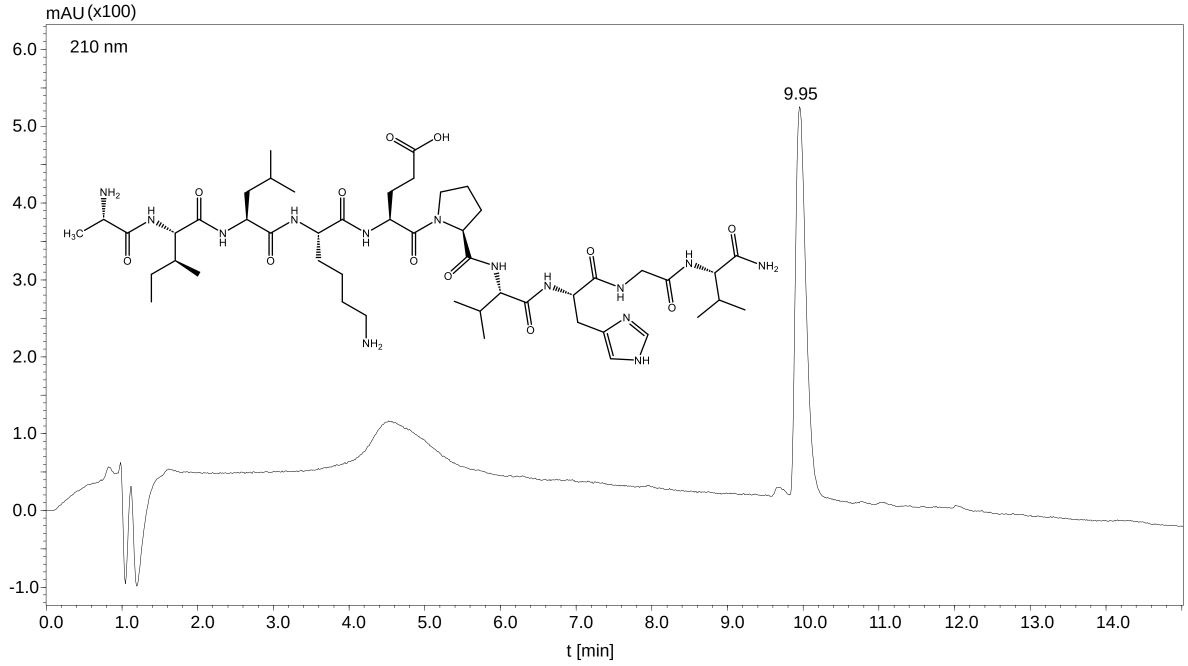

Fig S 64. HPLC chromatogram obtained for the purified peptide H-Ala-Ile-Leu-Lys-Glu-Pro-Val-His-Gly-Val-NH_2 after desulfurization

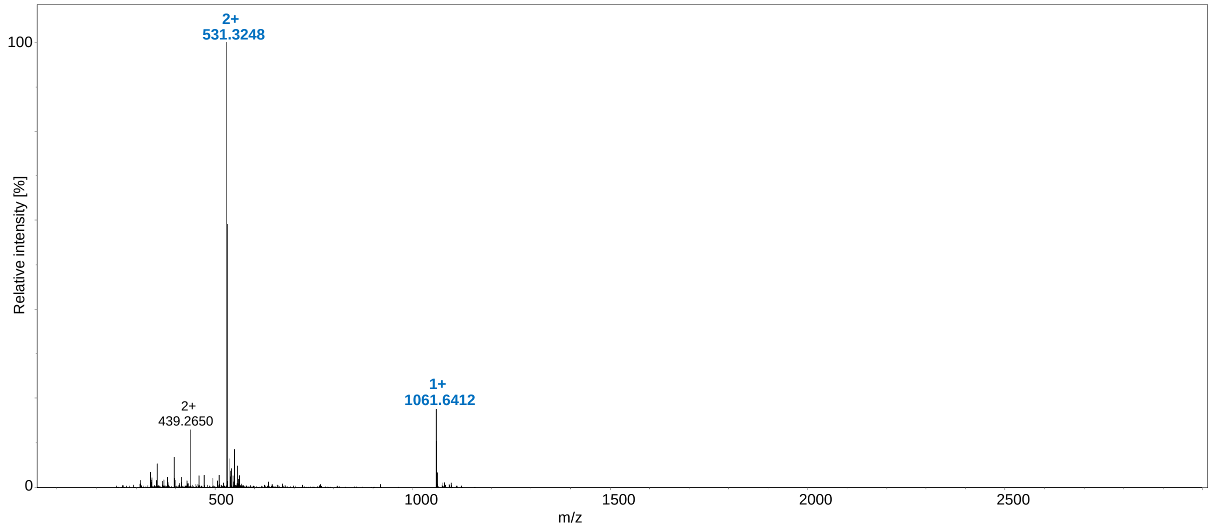

Fig S 65. ESI-MS spectrum obtained for purified peptide H-Ala-Ile-Leu-Lys-Glu-Pro-Val-His-Gly-Val-NH_2 after desulfurization

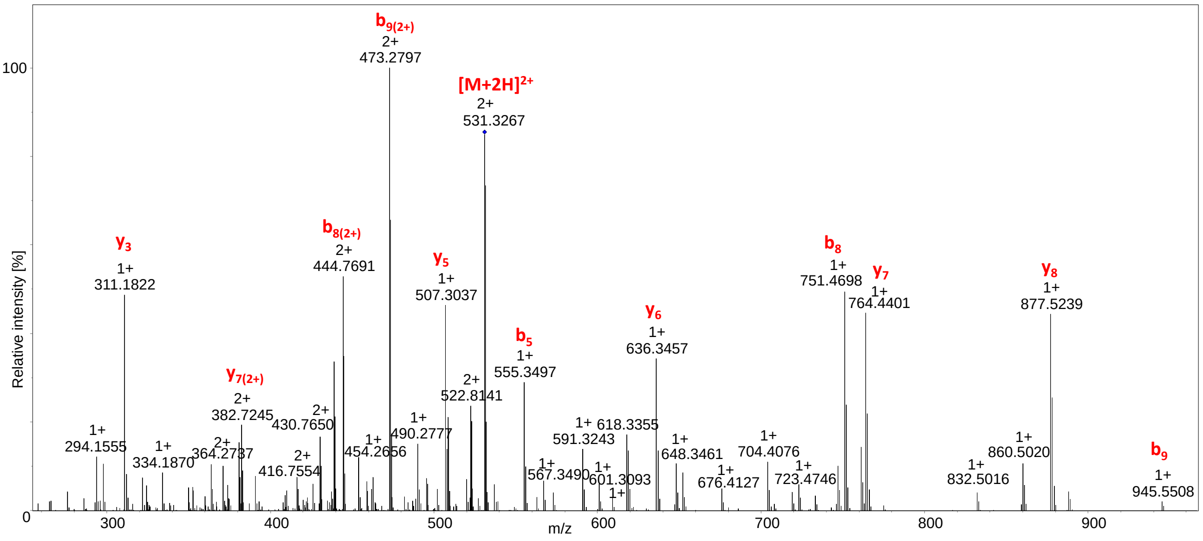

Fig S 66. ESI-MS/MS (CE 25 eV) spectrum obtained for purified peptide H-Ala-Ile-Leu-Lys-Glu-Pro-Val-His-Gly-Val-NH_2 after desulfurization

14.12 One-pot native chemical ligation (NCL)-desulfurization

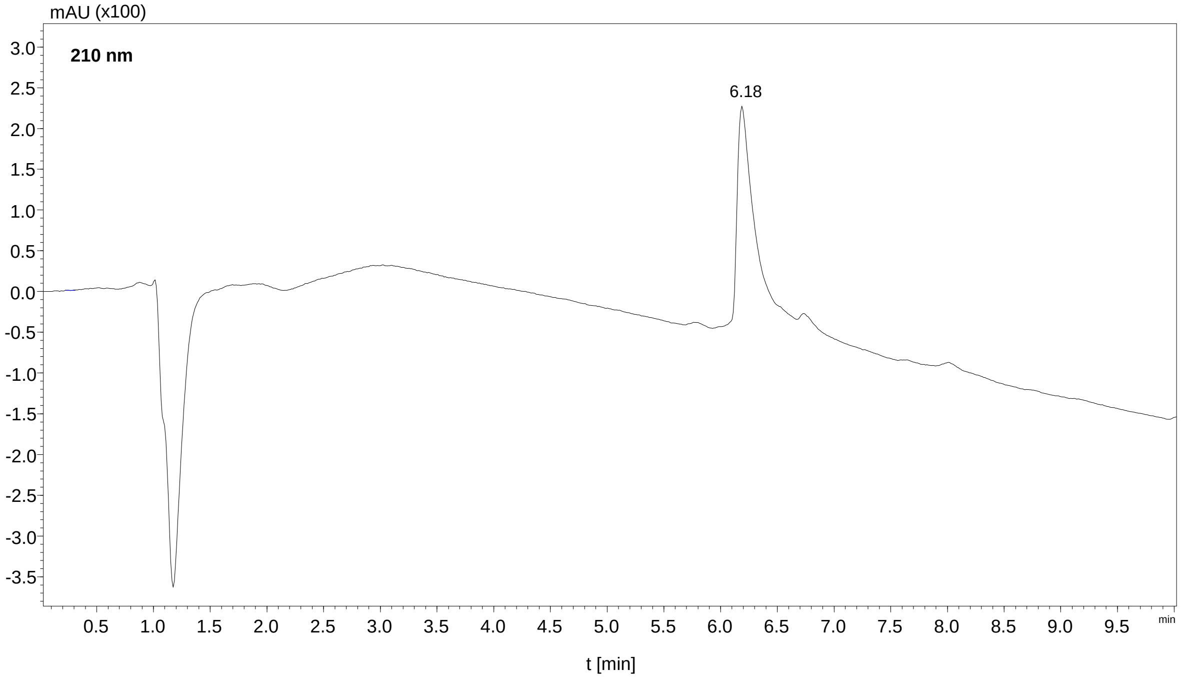

Fig S 67. HPLC chromatogram of purified peptide H-Gly-Thr-Phe-Thr-Ser-Asp-Val-Ser-Ser-Tyr-Leu-Glu-Gly-Gln-Ala-$NHNH_2$

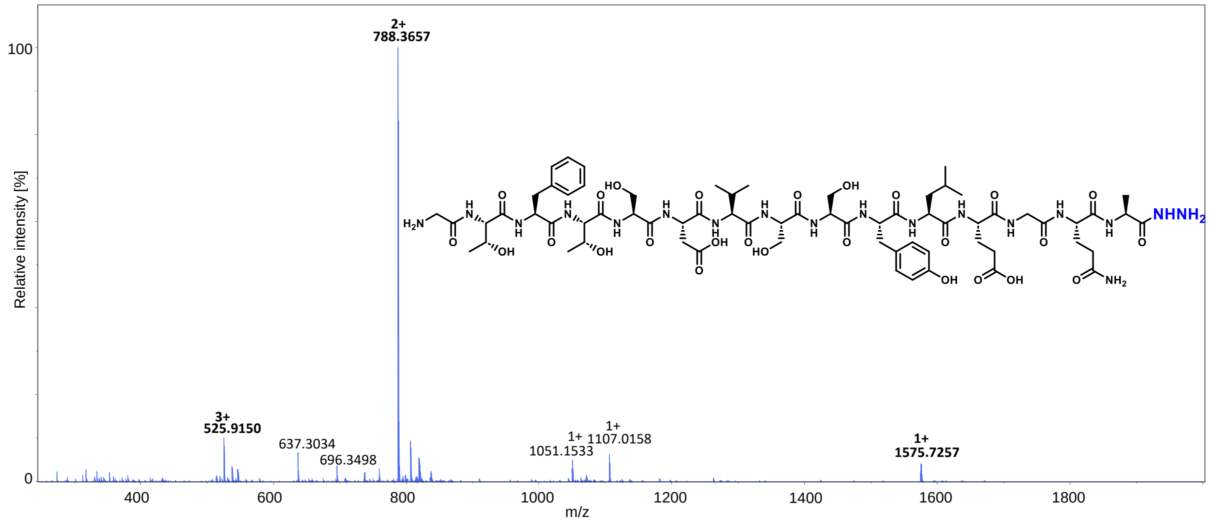

Fig S 68. ESI-MS spectrum of purified peptide H-Gly-Thr-Phe-Thr-Ser-Asp-Val-Ser-Ser-Tyr-Leu-Glu-Gly-Gln-Ala-NHNH$_2$

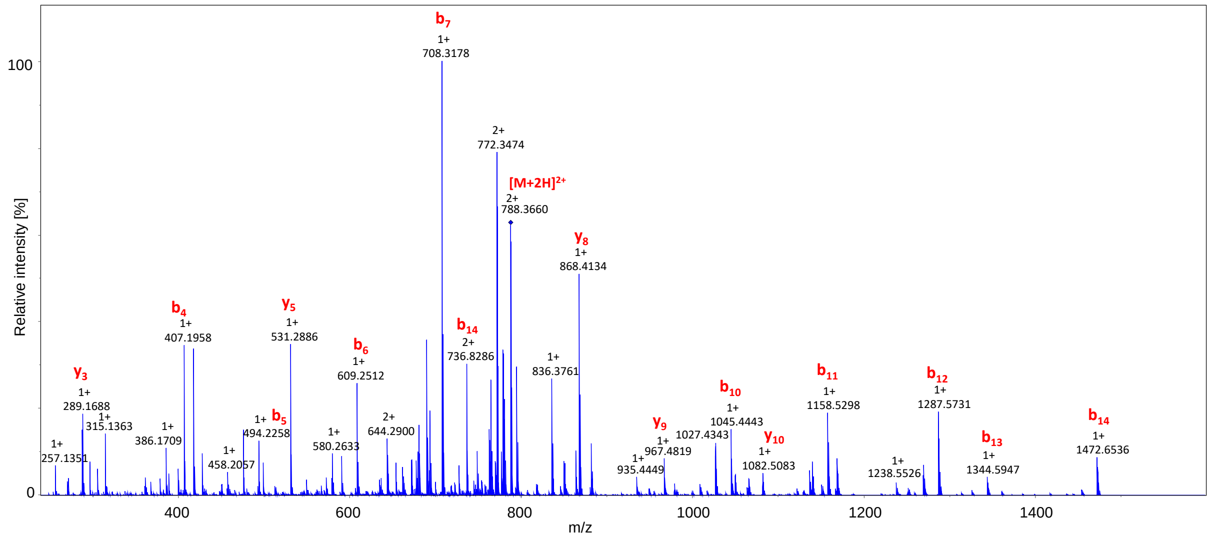

Fig S 69. ESI-MS/MS (CE 30 eV) spectrum of peptide H-Gly-Thr-Phe-Thr-Ser-Asp-Val-Ser-Ser-Tyr-Leu-Glu-Gly-Gln-Ala-NHNH$_2$

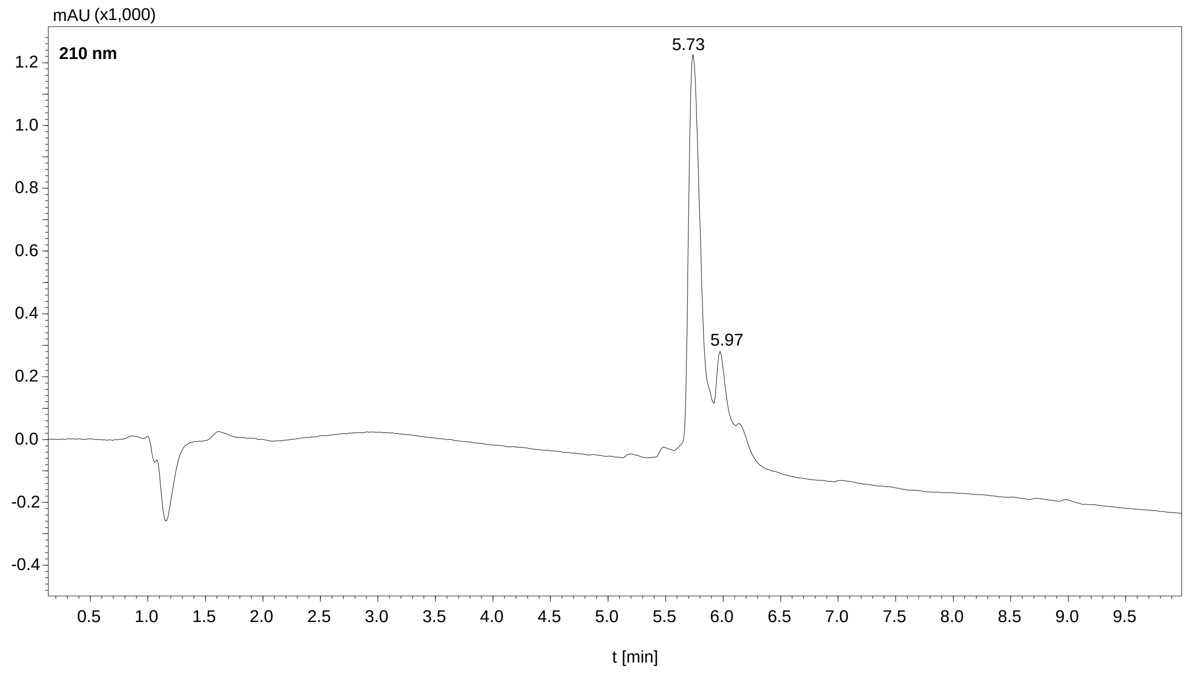

Fig S 70. HPLC chromatogram of peptide H-Cys-Lys-Glu-Phe-Ile-Ala-Trp-Leu-Val-Arg-Gly-Arg-Gly-OH.

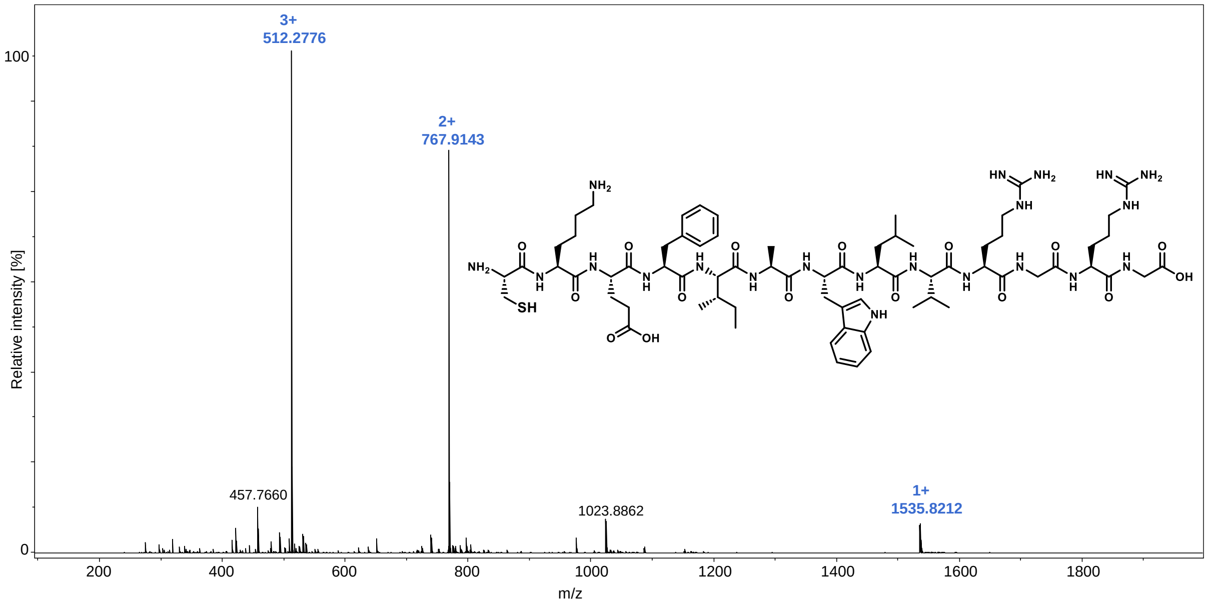

Fig S 71. ESI-MS spectrum of peptide H-Cys-Lys-Glu-Phe-Ile-Ala-Trp-Leu-Val-Arg-Gly-Arg-Gly-OH.

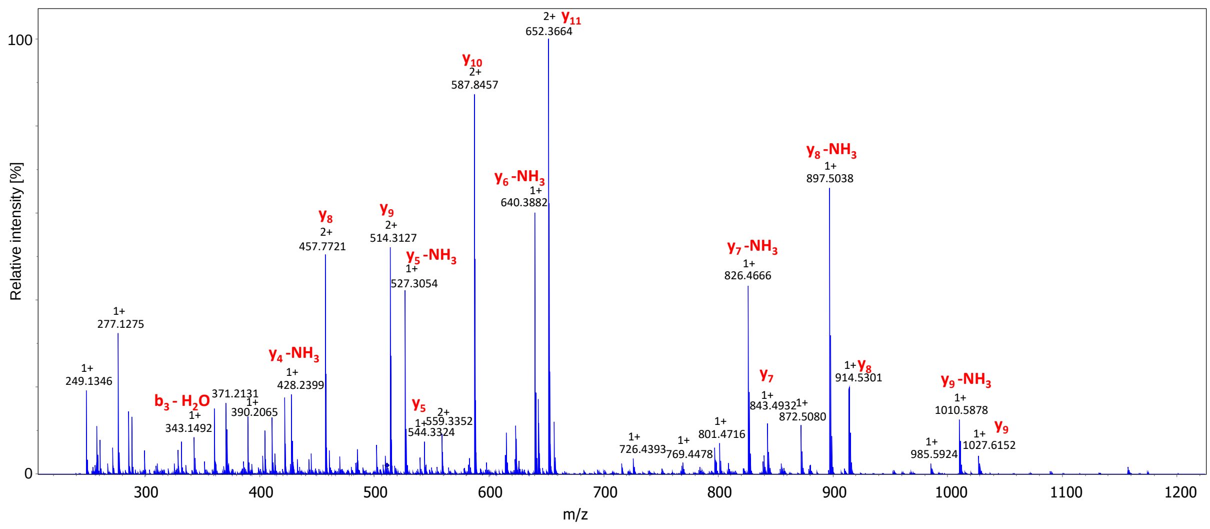

Fig S 72. ESI-MS/MS (CE 30 eV) spectrum of peptide H-Cys-Lys-Glu-Phe-Ile-Ala-Trp-Leu-Val-Arg-Gly-Arg-Gly-OH.

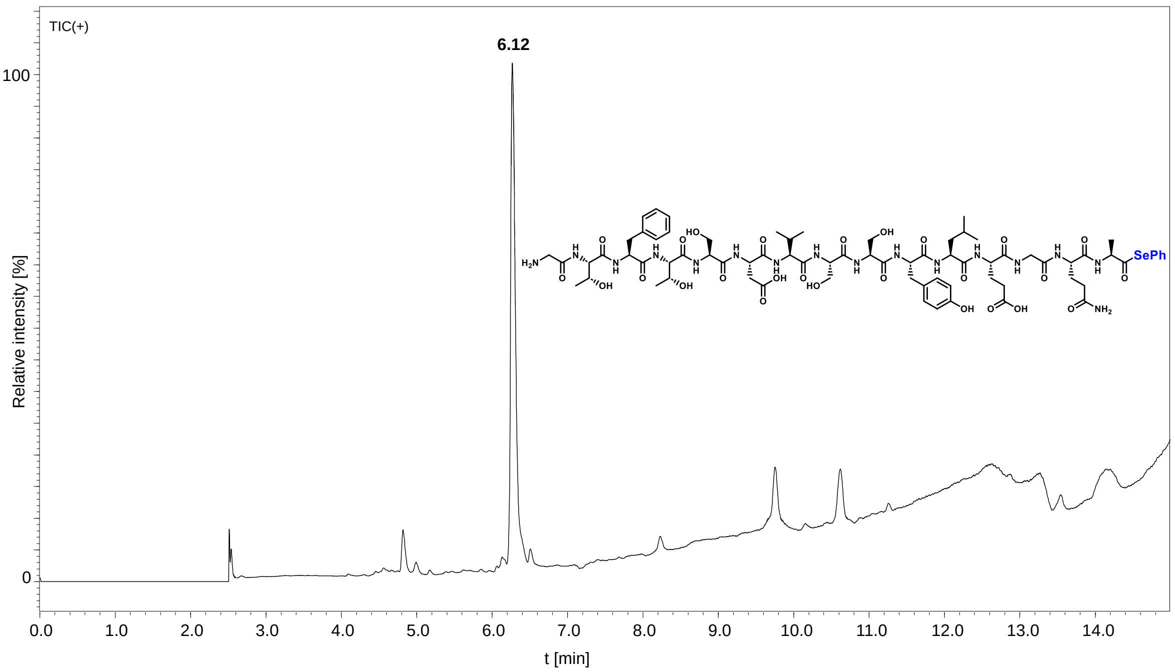

Fig S 73. LC-MS chromatogram obtained after converse of peptide H-Gly-Thr-Phe-Thr-Ser-Asp-Val-Ser-Ser-Tyr-Leu-Glu-Gly-Gln-Ala-NHNH$_2$ to C-terminal selenoester.

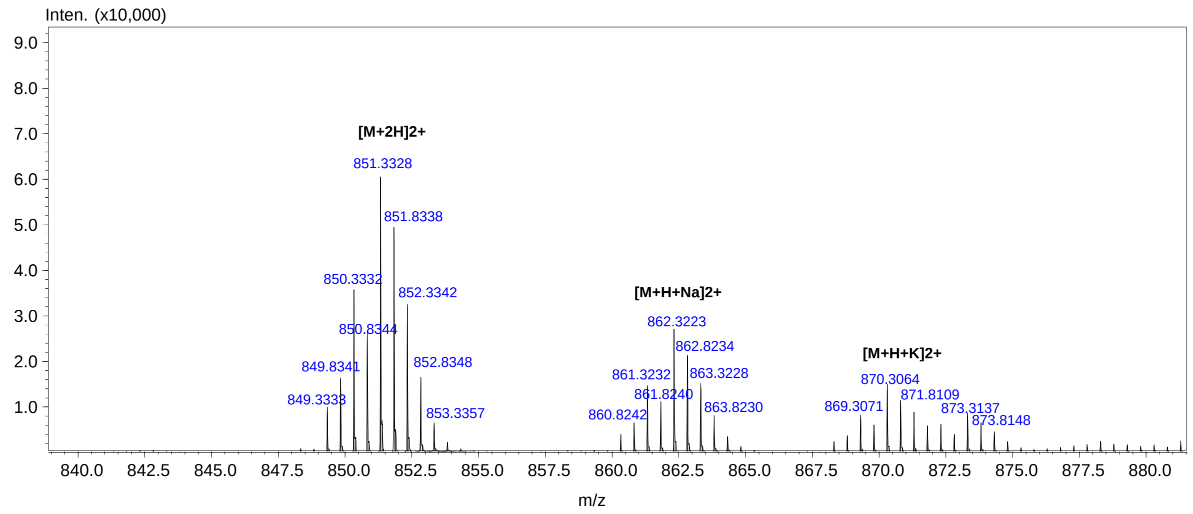

Fig S 74. LC-MS spectrum of H-Gly-Thr-Phe-Thr-Ser-Asp-Val-Ser-Ser-Tyr-Leu-Glu-Gly-Gln-Ala-SePh at 6.12 min (expanded area with characteristic isotopic pattern).

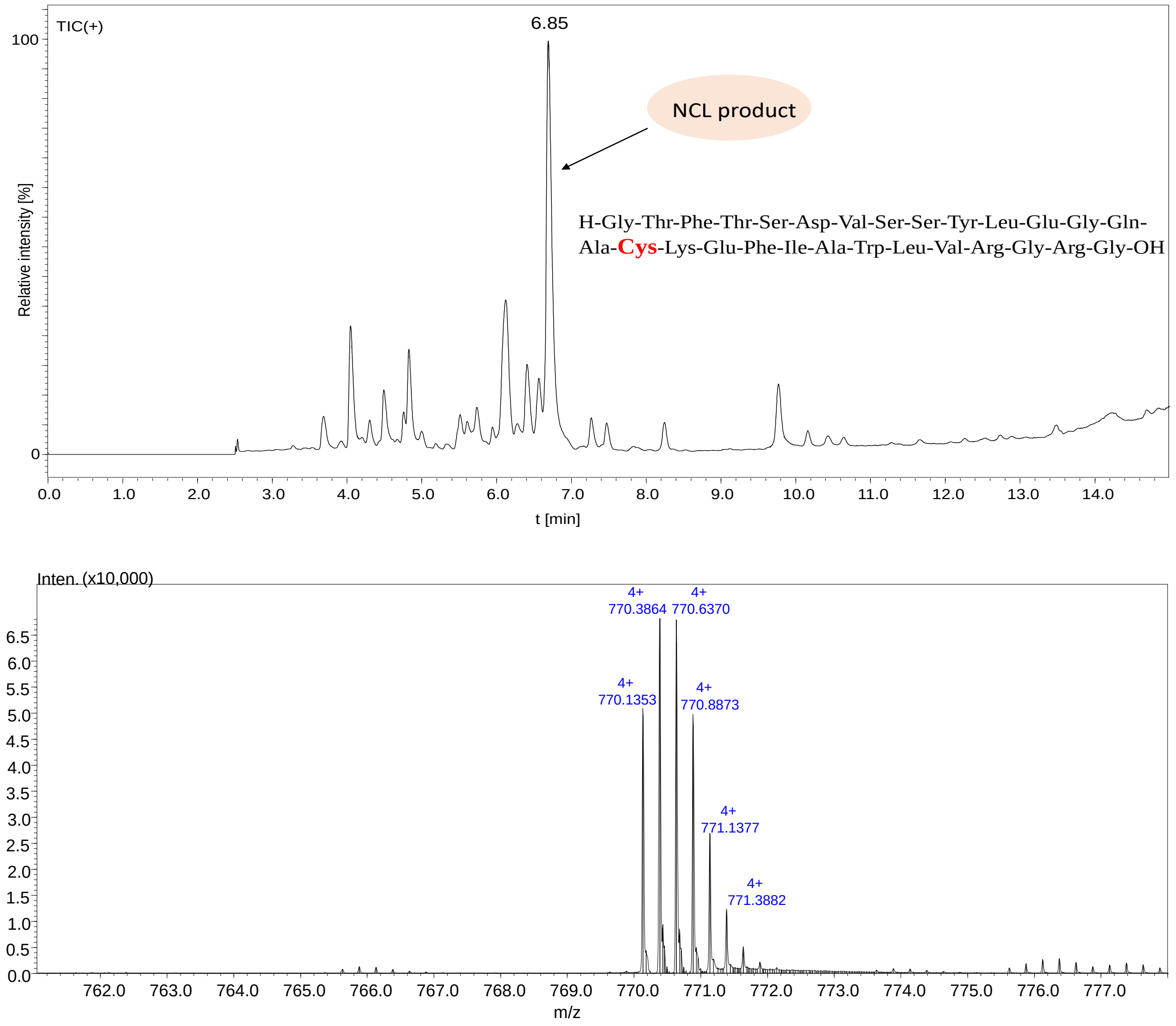

Fig S 75. LC-MS spectrum obtained for the mixture after native chemical ligation (NCL) and the corresponding ESI-MS spectrum for ligation product.

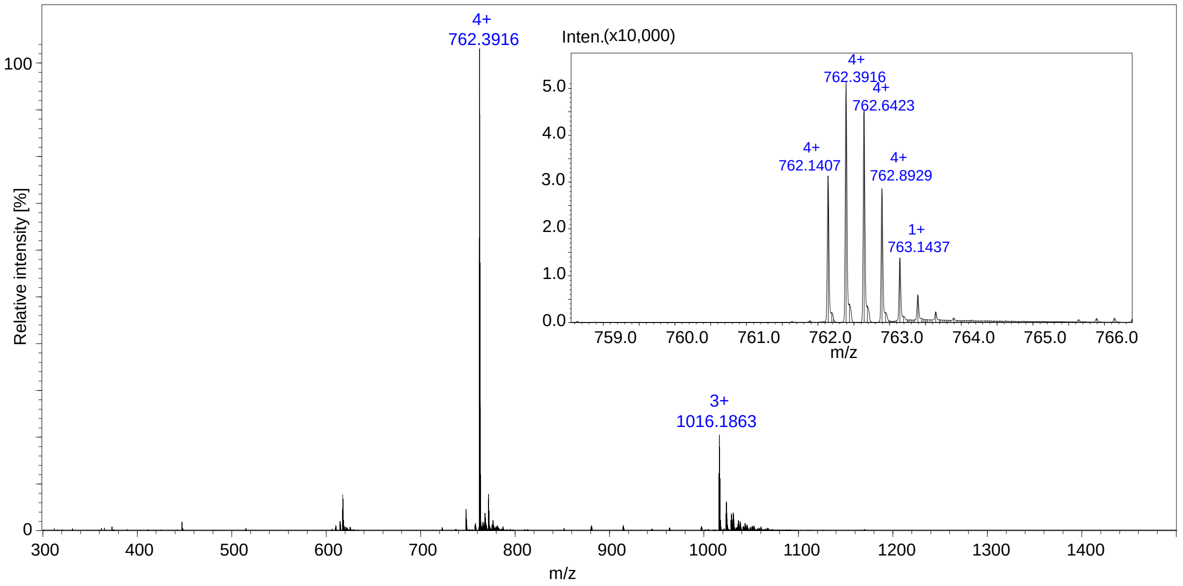

Fig S 76. LC-MS spectrum (6.8 min) of NCL-desulfurization product H-Gly-Thr-Phe-Thr-Ser-Asp-Val-Ser-Ser-Tyr-Leu-Glu-Gly-Gln-Ala-**Ala**-Lys-Glu-Phe-Ile-Ala-Trp-Leu-Val-Arg-Gly-Arg-Gly-OH.

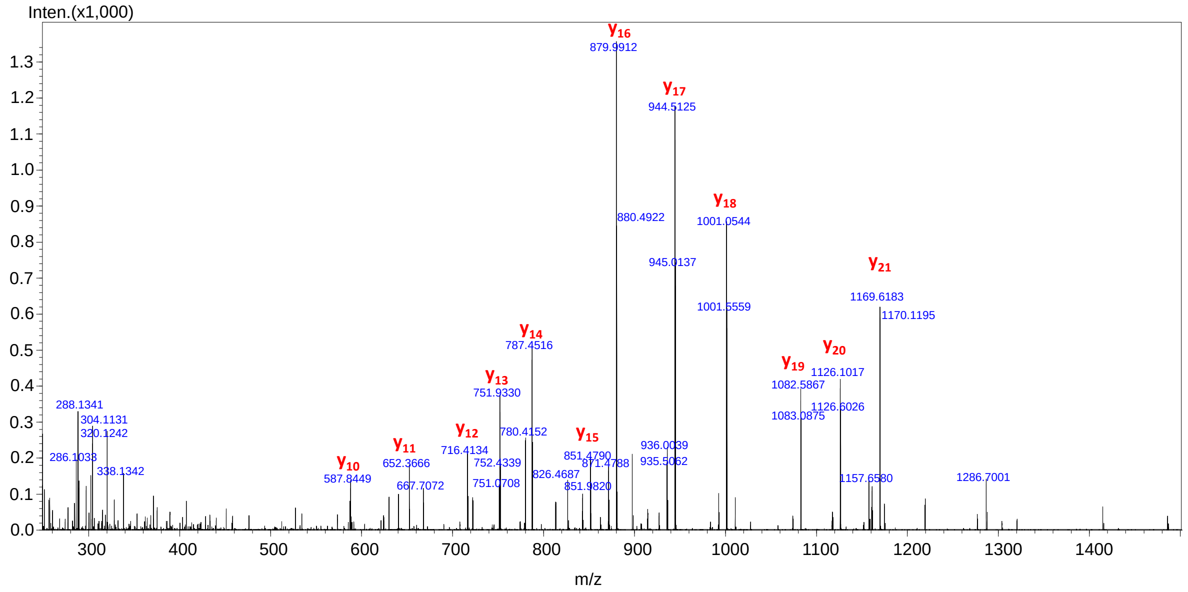

Fig S 77. LC-MS/MS (CE 35 eV) spectrum (6.8 min) of NCL-desulfurization product H-Gly-Thr-Phe-Thr-Ser-Asp-Val-Ser-Ser-Tyr-Leu-Glu-Gly-Gln-Ala-**Ala**-Lys-Glu-Phe-Ile-Ala-Trp-Leu-Val-Arg-Gly-Arg-Gly-OH.

15. Mechanistic study of desulfurization experiment

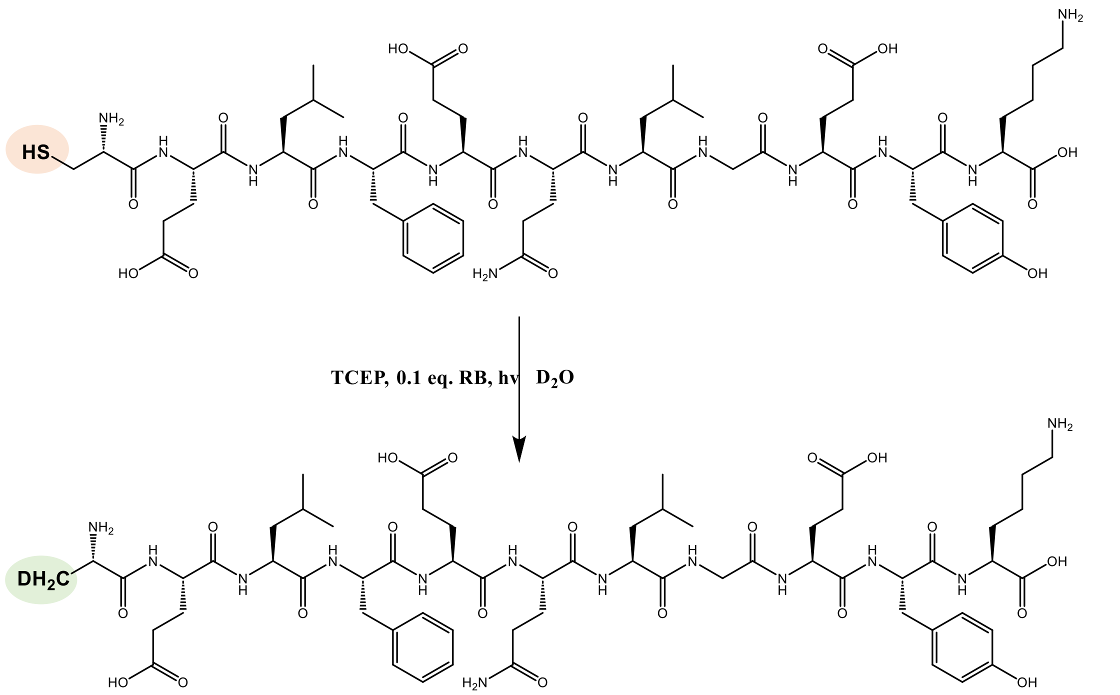

Fig S 78. Schematic representation of desulfurization of model peptide H-Cys-Glu-Leu-Phe-Glu-Gln-Leu-Gly-Glu-Tyr-Lys-OH in D_2O.

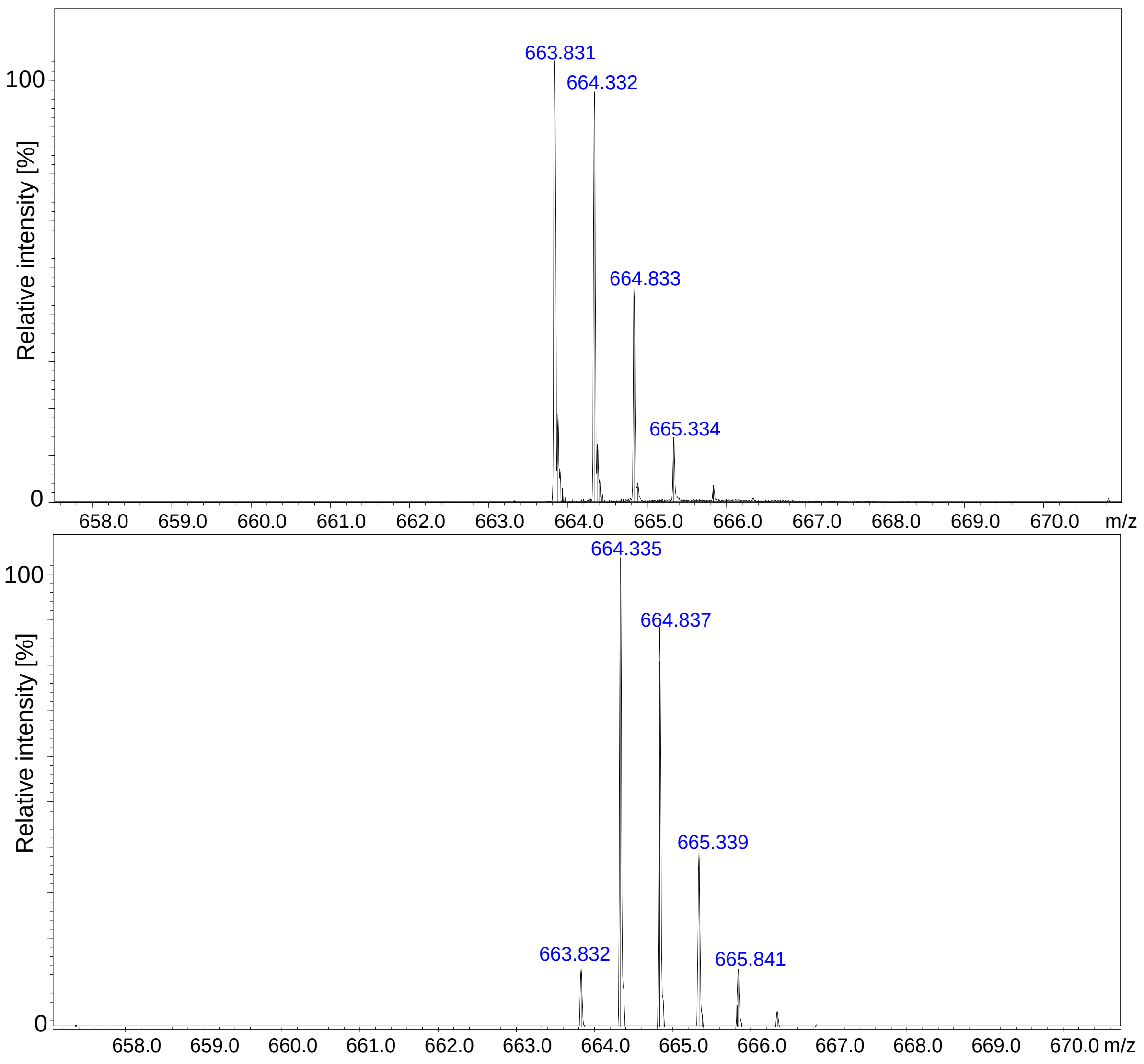

Fig S 79. MS spectrum obtained for desulfurization product of peptide H-Cys-Glu-Leu-Phe-Glu-Gln-Leu-Gly-Glu-Tyr-Lys-OH - Upper spectrum - reaction in H_2O buffer; Bottom spectrum - reaction in D_2O solution

16. Desulfurization of proteins

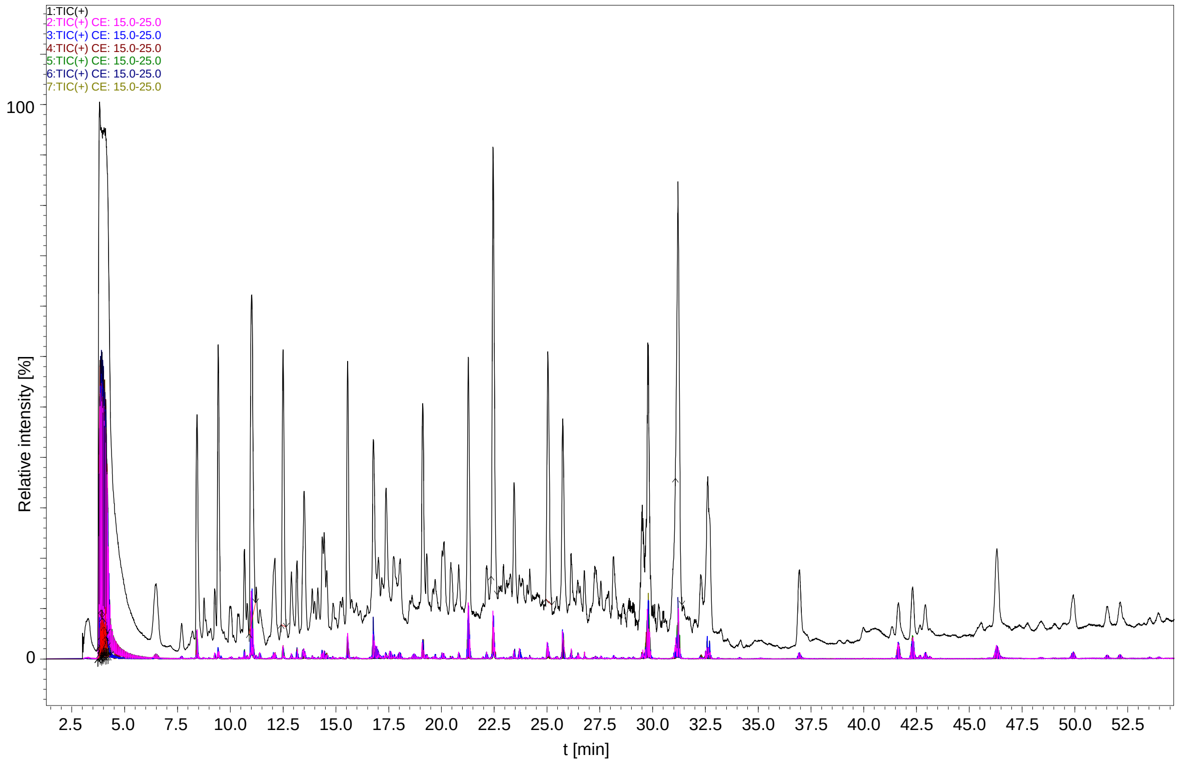

Fig S 80. LC-MS/MS (TIC) chromatogram obtained for tryptic digest of α-crystallin.

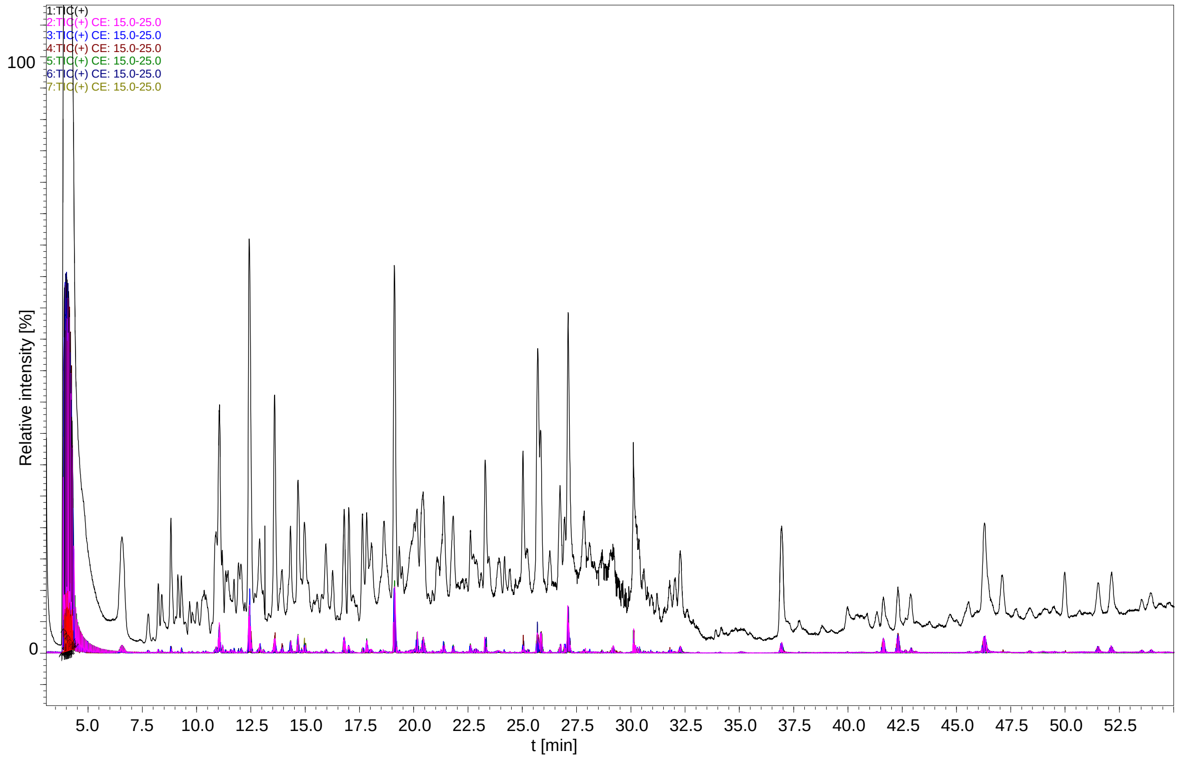

Fig S 81. LC-MS/MS (TIC) chromatogram obtained for tryptic digest of lysozyme C.

1 MDIAIQHPWF KRTLGPFYPS RLFDQFFGEG LFEYDLLPFL SSTISPYYRQ SLFRTVLDSG ISEVRSDRDK FVIFLDVKHF   d Desulfurization (-31.97)

131 d

81 SPEDLTVKVQ EDFVEIHGKH NERQDDHGYI SREFHRRYRL PSNVDQSALS CSLSADGMLT FSGPKIPSGV DAGHSERAIP

161 VSREEKPSSA PSS

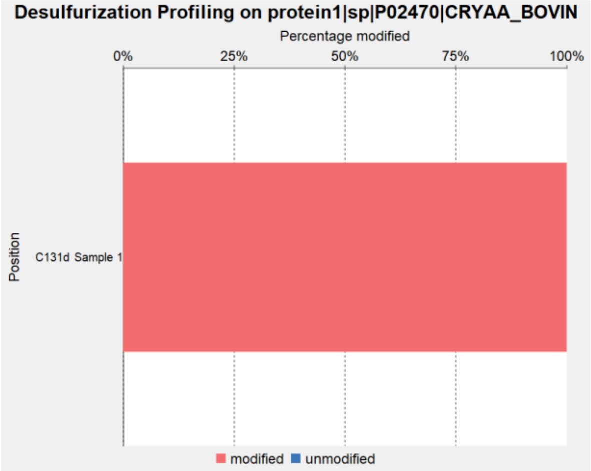

Fig S 82. Sequence coverage of α-crystallin tryptic digest with desulfurized cysteine residue highlighted and table showing the percentage efficiency of desulfurization of α-crystalline on the position 131.
